# Designing yeast as plant-like hyperaccumulators for heavy metals

**DOI:** 10.1038/s41467-019-13093-6

**Published:** 2019-11-08

**Authors:** George L. Sun, Erin. E. Reynolds, Angela M. Belcher

**Affiliations:** 1 Department of Biological Engineering, Massachusetts Institute of Technology, Cambridge, MA 02139 USA; 2 Koch Institute of Integrative Cancer Research, Massachusetts Institute of Technology, Cambridge, MA 02139 USA; 3 Department of Civil and Environmental Engineering, Massachusetts Institute of Technology, Cambridge, MA 02139 USA; 4 Department of Material Science, Massachusetts Institute of Technology, Cambridge, MA 02139 USA

**Keywords:** Environmental biotechnology, Bioremediation, Metabolic engineering

## Abstract

Hyperaccumulators typically refer to plants that absorb and tolerate elevated amounts of heavy metals. Due to their unique metal trafficking abilities, hyperaccumulators are promising candidates for bioremediation applications. However, compared to bacteria-based bioremediation systems, plant life cycle is long and growing conditions are difficult to maintain hindering their adoption. Herein, we combine the robust growth and engineerability of bacteria with the unique waste management mechanisms of plants by using a more tractable platform-the common baker’s yeast-to create plant-like hyperaccumulators. Through overexpression of metal transporters and engineering metal trafficking pathways, engineered yeast strains are able to sequester metals at concentrations 10–100 times more than established hyperaccumulator thresholds for chromium, arsenic, and cadmium. Strains are further engineered to be selective for either cadmium or strontium removal, specifically for radioactive Sr^90^. Overall, this work presents a systematic approach for transforming yeast into metal hyperaccumulators that are as effective as their plant counterparts.

## Abstract (web summary)

Existing heavy metal bioremediation systems are mainly based on plants, which require long growing time in specific conditions. Here, the authors mimic the characteristics of plant hyperaccumulators to engineer more tractable baker’s yeast and achieve 10–100-fold higher accumulation of chromium, arsenic, or cadmium.

## Introduction

Heavy metal contamination is a growing environmental concern as the world becomes increasingly industrialized. Mining, manufacturing, and disposal of electronic goods are the main sources of heavy metal waste; the United States alone adds 262 million tonnes (289 tons) of waste per year to the growing 850 and more landfills^1^. To illustrate the impact of waste generation this work specifically looked at two significant, yet often overlooked, contributors to heavy metal waste which are the textile industry and pollution from nuclear power plants and past fallout. Textile manufacturing employs a variety of heavy metal related processes, in particular dyeing, with many of the 100,00 types of dyes containing metal chelated centers for coloration^2^. Particular regions, such as India and Bangladesh where textile manufacturing is a dominant industrial practice, see high levels of cadmium, chromium, and lead in soils which can reach 10–100 times higher than WHO established safety limits^3^. Other metals such as cobalt, copper, zinc, and nickel are also pervasive and are incorporated at different levels in the textile process^2,3^. The result, leachate that contains an indiscriminate mixture of metals which are difficult to separate, therefore leaving burial or transport to remote areas as the only viable waste management option. On the same vein, the problem of nuclear waste and past nuclear fallout, such as previous catastrophic events of Chernobyl and Fukushima, have refocused attention on radioactive metal contamination, specifically radioactive strontium (Sr^90^) which is of particular interest for its biological implications in bone integration and cancer^4–6^. However, given the molecular similarity of calcium and strontium, and the relative abundance of calcium over strontium, removing just Sr^90^ without being overwhelmed by other species is challenging. Both waste scenarios expose a unique challenge, how to selectively capture and discriminate metals from one another. Removal of toxic elements such as cadmium and mercury should be prioritized, even if at lower concentrations than more abundant and less harmful elements such as calcium and magnesium. This is particularly true for radioactive elements such as Sr^90^, where strontium is typically masked by large amounts of similar divalent metals like calcium. Current industrial approaches such as absorption and ion-exchange are not particularly effective for precise removal of toxic yet low concentration of heavy metals as these processes are first saturated by more abundant background metals^7–9^.

Bioremediation strategies have the potential to address the challenge of heavy metal contamination. A promising subset of bioremediation is phytoremediation, the use of plants to sequester pollutants from soils and water^10,11^. Plants have developed mechanisms to uptake heavy metals without suffering major toxic effects, and their abundant and renewable biomass contribute to significant bioaccumulation of toxins from soils and waters^10,11^. Out of all plants, there are more than 400 hyperaccumulator species; the stricter definition being an accumulation of 100 mg/kg of dry weight (DW) (0.01% DW) of cadmium or arsenic, 1000 mg/kg (0.1% DW) of cobalt, copper, chromium, aluminum, nickel, or lead, and 10,000 mg/kg (1% DW) of manganese, iron, or zinc^12–14^. Not all hyperaccumulators have equal metal preferences. Even in a single family such as Brassicaceae, out of the 87 species 67 are nickel hyperacumulators, 15 are zinc, and 5 can do both^12^. Insights on the mechanism of hyperaccumulation have been attributed to hyperactive metal transporters and a variety of detoxification pathways which include glutathione synthesis and metal compartmentalization in vacuoles and other organelles^15,16^.

What limits wide-spread adoption of plant-based remediation solutions is their maintenance and engineering complexity. Plants are complex organisms, with different species requiring strict growing conditions where hyperaccumulators found in one location may not necessarily thrive in others due to surrounding biotic and abiotic factors. More so, current phytoremediation technology takes weeks to years to see signs of remediation, and in this current global waste crisis may be too long of a time scale^17–19^. There have been attempts to create transgenic plants which incorporate genes from hyperaccumulators which grow faster and are more resistant to environmental factors^20^. However, because plants are multi-cellular with more complex gene clusters, the current state of genetic tools have yet to realize the sophistication and ease of engineering compared to their single-celled counterparts such as bacteria and yeast^21^. Therefore, design of faster and easier waste management technologies need to be developed on other platforms that are more scalable and cost-effective. Single-cell organisms such as bacteria offer ease and scalability; however, they lack many hyperaccumulating features such as hyperactive metal transporters and useful organelles such as a vacuole. A biological platform at the intersection of these two is the common baker’s yeast, *S. cerevisiae*. Current genetic engineering technologies have made it possible to engineer yeast on all levels, from specific proteins to complex metabolic pathways. More so, the infrastructure and ability to scale and distribute yeast are already in place thanks to the beer and pharmaceutical indutries^22–24^. The results herein show that taking concepts from plant hyperaccumulators and engineering them into yeast can generate similar hyperaccumulating capabilities that are equal or better than their plant-based counterparts.

## Results

### Expressing metal transporters increase metal uptake

**Fig. 1:**
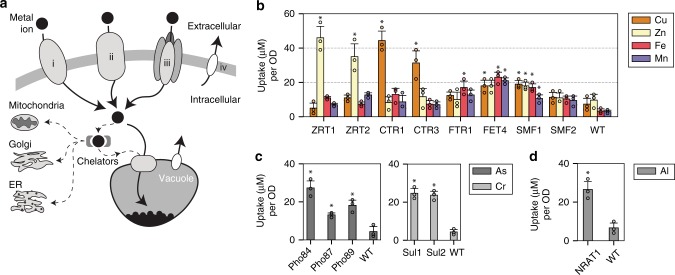
Metal transporters were used to selectively internalize heavy metals into yeast. **a** A simplified schematic of metal transport in a eukaryotic cell. Membrane transporters can be divalent metal transporters (i), permeases (ii), metal transporters that are modified or found to have auxillary metal transport function (iii), or exporters which are used to remove excess metals out of the cell (iv). **b** Bar coloring indicates metal measured, with over-expressed transporter labeled on the *x*-axis. Values are reported in μM of metal uptake normalized per yeast culture density (*μ*M/OD). Yeast metal transporters for zinc (ZRTs), copper (CTRs), iron (FTRs and FETs), and manganese (SMFs) were overexpressed and studied for metal hyperaccumulation. A WT strain was also tested in parallel as a control. **c** The same study was performed for phosphate and sulfate permeases (PHOs, and SULs) which showed transport of arsenate and chromate, respectively. **d** The Nrat1 transporter, previously shown to uptake trivalent metals in certain strains of rice, was expressed and showed aluminum(III) transport. Asterisk above bar charts represent significance increase in uptake compared to WT (*p* < 0.05) for strains mentioned in the text. For all data, the mean ± s.d. of three replicates are shown. The source data underlying Fig. 1b are provided as a Source Data file

Several fundamental metal trafficking components are essential for enhanced metal uptake in hyperaccumulating plants, including cell membrane transporters, organelles storage systems, and chelator molecules^15,16^ (Fig. 1a). Metal ions enter via highly active membrane transporters, and are compartmentalized into organelles such as the vacuole. To limit cellular toxicity, chelators such as glutathione, metallothionein, and phytochelatins bind and remove metals from sensitive metabolic functions^16^. To mimic these plant hyperaccumulating features, the first step was to identify and express a hyperactive membrane transporter. A set of membrane metal transporters for zinc, copper, iron, and manganese^13,15,25–27^ were overexpressed in yeast. For this study, native yeast metal transporters ZRT1 (accession number #P32804), ZRT2 (#Q12436), CTR1 (#P49573), CTR3 (#Q06686), FTR1 (#P40088), FET4 (#P40988), SMF1 (#P38925), and SMF2 (#P38778) (ZRT3, CTR2, and SMF3 are vacuole transporters, while FET3 is an oxidoreductase) were cloned and overexpressed using a GAL1 promoter on a 2μ plasmid. When overexpressed, some of the transporters, along with several more described below, did not show uniform expression but instead had punctate patterns when examined under fluorescence microscopy (Supplementary Fig. 1a). This suggested that over-expression led to poor localization, and this factor was considered when selecting a transporter candidate for future engineering.

To measure metal uptake, cells were incubated in 100 μM metal for 4 h. Supernatant was collected and measured for remaining metal content using inductive coupled plasma (ICP), and this value was used to calculate the amount of metal removed by the cells. Parallel to each experiment a sample of wild-type (WT) and a sample with no cells were measured as controls. In addition, samples were washed in both ddH_2_O and a 1 mM EDTA buffer and measured for freed metals to account for non-specific metal binding onto the cell wall. No major non-specific metal binding was observed in either ddH_2_O or EDTA wash steps (Supplementary Fig. 2). Taking these controls into consideration, enhanced uptake of zinc, copper, iron, and manganese was observed across several transporters (Fig. 1b). ZRT1,2 and CTR1,3 were highly selective for zinc and copper, respectively, increasing metal uptake by 10-fold compared to wild-type (WT) (*p* < 0.05). FET4 and SMF1 were less metal-specific and increased metal uptake by 3–5 fold across all four metals (*p* < 0.05; except for FET4 uptake of Zn compared to WT).

A similar study was performed for arsenic and chromium. These metals are typically found in oxy-polyatomic states such as arsenate and chromate. To achieve arsenate and chromate hyperaccumulation a different set of transporters were needed. Given the molecular and steric similarity between phosphate (PO_4_^3−^) and arsenate (AsO_4_^2−^), and sulfate (SO_4_^2−^) and chromate (CrO_4_^2−^), a hypothesis was that the overexpression of sulfate and phosphate permeases would allow passage of arsenate and chromate^28,29^. Overexpression of phosphate permeases Pho84 (#P25297), 87 (#P25360), and 89 (#P38361), and sulfate permease Sul1 (#P38359) and Sul2 (#Q12325) showed increased metal uptake of arsenate and chromate, respectively (Fig. 1c; Supplementary Fig. 1a). Overall, the Pho genes increased arsenate uptake by more than 3–5 fold (*p* < 0.05), and Sul genes increased chromate uptake by more than 5-fold (*p* < 0.05). These observations align with plant hyperaccumulation observations that arsenate and chromate trespass into the cell via the phosphate and sulfate assimilation pathways^30,31^.

Another common group of metal contaminants are trivalent metal ions such as aluminum and rare-earth metals. The most obvious approach would be to use a trivalent metal transport for their metal uptake; however, none exist in yeast, or generally at all. But research in a rice specie, *Oryza sativa*, uncovered a Nramp-like transporter known as Nrat1 (#Q6ZG85) which showed selective uptake of aluminum but not divalent metals^32^. Cloning and heterologously expressing Nrat1 in yeast did indeed promote selective uptake of aluminum with more than a 5-fold increase in aluminum uptake than compared to WT (*p* < 0.05) (Fig. 1d), and no significant uptake for divalent metals such as Cu, Zn, Fe, and Mn (*p* > 0.05) (Supplementary Fig. 3) which results align with previous published observations^32^. The use of Nrat1 for rare-earth metal uptake such as neodymium and ytterbium, precious metals used in magnets and electronics, were tested but gave unreliable results as they precipitated in culture before measurements could be performed. However, the preferential accumulation of aluminum using Nrat1 support the hypothesis that other trivalent metals such as lanthanides and actinides can be potentially hyperaccumulated.

To compare yeast hyperaccumulation results with established values, the amount of metal uptake was converted to milligram of metal removed per gram of culture dry weight (gDW) (Supplementary Fig. 4). Given these results, overexpression of CTR1,3 reached 7.5 ± 0.9 and 3.1 ± 0.7 mg/gDW for copper, and overexpression of FTR1 and FET4 reached 2.0 ± 0.4 and 2.5 ± 0.3 mg/gDW for iron, respectively (Supplementary Table 1). All phosphate (Pho84, 87, 89) and sulfate (Sul1, 2) permeases accumulated beyond the 1 mg/gDW threshold for arsenate and chromate hyperaccumulation. Nrat1 reached 1.25 ± 0.2 mg/gDW of aluminum which is above the 1 mg/gDW threshold^14^. Overall, these results show that hyperaccumulation is not a plant-specific trait but a generalizable feature that can be engineered in yeast by selecting and expressing the appropriate metal transporters.

### Increasing expression levels of SMF1 enhance metal uptake

SMF1 from the Nramp family was selected for further optimization and engineering because of its broad metal specificity (Fig. 1b), and the existing body of research on the Nramp family^33–36^. Another selection criterion was SMF1’s relatively consistent membrane-localized expression as observed under fluorescent microscopy (Supplementary Figs. 1a, 5). SMF1 was also favored because of its promiscuous activity with several metals such as manganese, iron, nickel, and cobalt^27,36,37^. Thus, SMF1 was a more appealing candidate to engineer for selective heavy metal uptake rather than converting a highly specific metal transporter which may be less malleable to change. More so, past work by Bozzi et. al. and Ehrnstorfer et. al. have elucidated crystal structures of multiple Nramps and have shed light on their structure-to-function relationship with respect to metal uptake^33,38^. These insights were leveraged to semi-rationally alter the metal preference of SMF1, which is shown in later results.

**Fig. 2:**
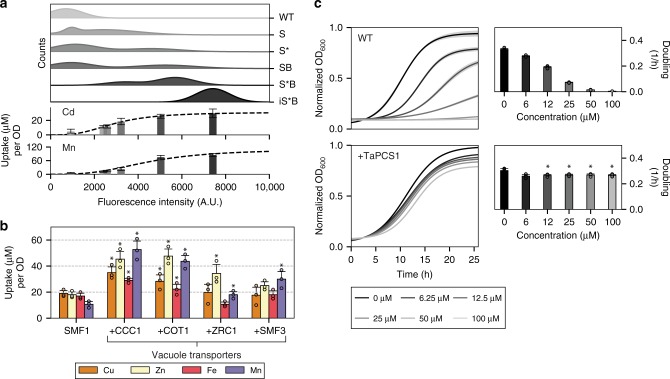
Modifying yeast metal trafficking pathways improved metal uptake and tolerance. **a** Top subpanel shows the population distribution of SMF1 variants measured with fluorescently labeled V5-tag using flow cytometry. The weighted average of the fluorescent intensity corresponds to the placement of the lower subpanel bar charts which represent the level of metal uptake for that strain. Increasing expression levels of SMF1 correlated to increased metal uptake of cadmium or manganese; however, up to a certain point indicated by the plateau in uptake. **b** Expression of vacuole transporters CCC1, COT1, ZRC1, and SMF3 in addition to SMF1 enhanced metal uptake. Asterisk above bar charts represent significant increase in uptake compared to SMF1 (*p* < 0.05). **c** Constitutively expressing wheat phytochelatin synthase, TaPCS1, conferred heavy metal tolerance against cadmium. Asterisk above bar charts represent significant changes in growth rates compared to WT (*p* < 0.01). For all data, the mean ± s.d. of three replicates are shown. The source data underlying Fig. 2b are provided as a Source Data file

Enhancing metal uptake using SMF1 required increasing its expression lifetime by increasing protein yield and stability. SMF1 (denoted as S), like most nutrient transporters, is tightly regulated to control the flux of metals into the cell, while limiting excess uptake to protect against toxicity. SMF1 expression, for example, is controlled by manganese ions and is post-translationally downregulated by ubiquitination and endocytosis^39^. To create a hyperaccumulating yeast strain, these controls were removed so that the transporter could be highly expressed without degradation. Therefore, mutations of SMF1’s ubiquitination site K33,34 were altered to arginine (mutant denoted as S*) which helped reduce protein degradation^39^. In addition, BSD2 ubiquitin ligase (#P38356), which post-transcriptionally tags SMF1 for degradation, was deleted to further enhance SMF1 expression levels (deletion strain denoted as B)^37,40^. Finally, SMF1* was integrated (denoted as iS*) under a GAL promoter in BSD2 knockout strains. Expression was measured using both fluorescence microscopy (Supplementary Fig. 5), and quantified using flow cytometry by fluorescently labeling a V5 tag fused to the C’-terminus of the SMF1 variants. Populations of fluorescently labeled SMF1 were analyzed to measure the percent of positively expressing cells, and the mean fluorescent intensity was used to qualitatively correlate the expression level between cells to their metal uptake levels (Fig. 2a). Changes from S → S* → S*B → iS*B corresponded to increasing uptake of manganese and cadmium which resulted in uptake levels saturating to 85 ± 6.7 μM (8.2 ± 0.7 mg/gDW) for manganese and 22 ± 6.0 μM (4.3 ± 1.2 mg/gDW) for cadmium given the presence of 100 μM manganese or cadmium in culture (Fig. 2a).

### Adding vacuole transporters further enhance metal uptake

Metal uptake capacity was further enhanced by expressing vacuole transporters to compartmentalized metals internalized by SMF1. Native yeast vacuole transporters^26,27^ tested were CCC1 (#P47818), COT1 (#P32798), ZRC1 (#P20107), and SMF3 (#Q12078) which were individually expressed in S*B strains (Supplementary Fig. 1b and 6). All tested vacuole transporters showed elevated metal uptake for copper, zinc, iron, and manganese, with CCC1 and COT1 being the most significant across all metals (*p* < 0.05) **(**Fig. 2b). These results support the role that the vacuole broadly compartmentalizes metals from the cytosol. However, without the expression of SMF1, sole expression of vacuole transporters CCC1, COT1, ZRC1, and SMF3 in WT strains had negligible impact on copper, zinc, iron, and manganese uptake (*p* > 0.05) (Supplementary Fig. 7). These results suggest that the largest barrier to metal uptake is from the membrane transporter, in this case SMF1, which is responsible for initial metal internalization. It is only after metal enters a cell that the vacuole transporters are rendered useful.

### Phytochelatin synthase TaPCS1 enhances metal tolerance

The purpose of creating a metal hyperaccumulator becomes counterproductive if the cell dies and releases the internalized metals back into the media. Therefore, mechanisms for metal detoxification and tolerance are needed to increase cell viability, and in theory, give cells more time to endure and uptake metals. One of the main mechanisms found in plants for metal detoxification is the production of phytochelatins, oligomers of glutathione (GSH) with cysteine and carboxyl rich moieties that chelate metals such as copper and cadmium^13,15,20^. Yeast are able to produce glutathione via the GSH pathway, which naturally protects yeast from accumulation of toxic metals. However, there does not exist a phytochelatin synthase for robust metal detoxification like that in plants. Instead, yeast rely on GSH or cysteine-rich and low molecular weight CUP1 metallothionein to chelate metals. However, past work has shown that metal detoxification is effective only at high copy numbers of CUP1^41^, suggesting that protein production versus chemical synthesis of metal chelating compounds is less effective, possibly due to a slower rate of protein synthesis and/or abundance. Therefore, to create yeast tolerant to heavy metal environments would require a similar phytochelatin synthase mechanism. Past studies in plant hyperaccumulators have shown that a phytochelatin synthase, TaPCS1 (#Q9SWW5), from wheat improved heavy metal tolerance in both plants and yeast^42^.

Integrating TaPCS1 under constitutive expression using a GAP promoter showed cadmium tolerance beyond 100 μM, whereas WT growth rates were significantly hampered below 10 μM (*p* < 0.01) (Fig. 2c), results which support past observations^42^. TaPCS1 also improved copper, manganese, zinc, and cobalt tolerance by 2–10 fold than compared to WT (Supplementary Fig. 8). The subsequent results which combine SMF1, CCC1, and TaPCS1 show that these modules can act additivity to incrementally improve metal hyperaccumulation.

### Engineering a manganese and cadmium hyperaccumulator

**Fig. 3:**
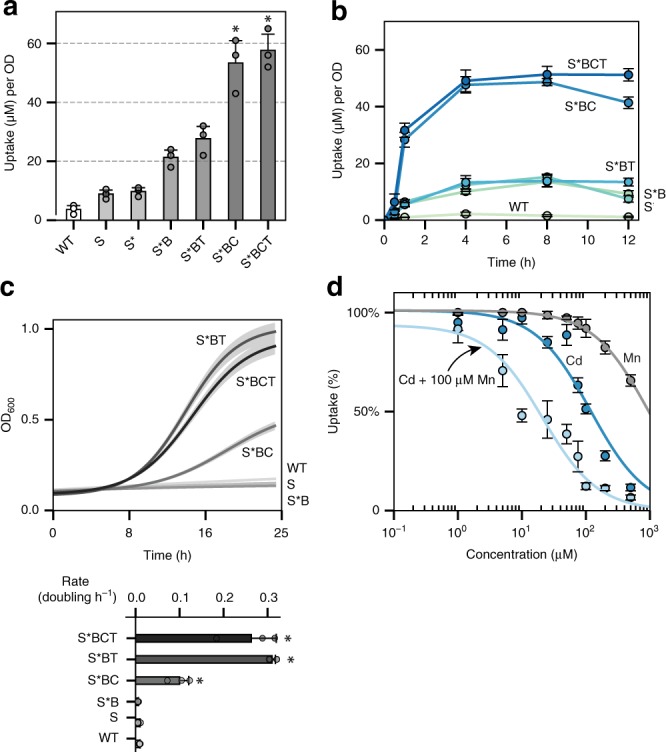
Combining SMF1, CCC1, and TaPSC1 improved metal uptake capacity and tolerance. **a** SMF1 (S) and its modifications (S* and ΔBSD2 as B) along with vacuole transporter CCC1 (C) and metal resistance enzyme TaPCS1 (T) incrementally enhanced cadmium uptake. Asterisk above bar charts represent significant increase in cadmium uptake when compared to WT (*p* < 0.01). **b** Combinations of S*, B, C, and T showed changes in uptake rate, capacity, and metal retention over 12 h of metal incubation. **c** In the presence of 100 μM cadmium, the growth rate is rescued with the addition of CCC1 and furthermore with TaPCS1. Subfigure below represents the doubling time of each strain. Asterisk to the side of bar charts represent significant increase in growth rate compared to WT (*p* < 0.01). **d** S*BCT strain was titrated against cadmium, manganese, or cadmium in the presence of 100 μM manganese (*x*-axis). Metal uptake experiments were performed at varying concentrations from 1 μM to 1 mM, metal content analyzed using ICP, and values reported as percent uptake. S*BCT showed a higher preference for manganese than cadmium, with cadmium uptake being dramatically reduced in the background presence of 100 μM manganese (light blue curve). For all data, the mean ± s.d. of three replicates are shown. The source data underlying Figs. 3a, 3b, and 3d are provided as a Source Data file

To mimic the characteristics of a plant hyperaccumulator, the final yeast-based system combined expression of the membrane transporter SMF1 (S, or K33,34R mutant S*), vacuole transporter CCC1 (C), metal detoxifying phytochelatin synthase TaPCS1 (T), and deletion of ubiquitin ligase BSD2 (B). All parts were integrated into the genome except for S* which was introduced on a 2μ plasmid under a GAL1 promotor. As each component was added to the system the amount of cadmium uptake increased incrementally. The effect of adding all components together (S*BCT) enhanced cadmium uptake by almost 16-fold than compared to WT (*p* < 0.01) (Fig. 3a). In addition, the rate of uptake increased dramatically with the combination S*BC reaching steady-states within 2–4 h compared to 10–12 h for strains lacking an overexpressed vacuole transporter (Fig. 3b). The rate of uptake increased by almost 30-fold for S*BCT compared to WT (*p* < 0.01). Adding T to S*B or S*BC did not significantly enhance metal uptake but instead stabilized metal internalization (Fig. 3a, b). After 12 h of growth in media containing 100 μM cadmium, strains without TaPCS1 began to leak back out cadmium, possibly due to cell death or activation of divalent exporters. In terms of viability, during active metal uptake in 100 μM cadmium, the expression of C slightly improved cell viability, whereas combined expression of C and T fully rescued yeast survival (*p* < 0.01) (Fig. 3c; Supplementary Fig. 9).

SMF1 and CCC1 have broad metal specificity primarily for row one transitions metals, thereby out-competing the uptake of cadmium if other transition metals such as manganese are present. To analyze the degree of manganese interference against cadmium, S*BCT was titrated at varying concentrations of cadmium with and without a constant background of 100 μM manganese. Metal uptake values were normalized to percent uptake with respects to the original metal concentration added, and the concentration at which metal uptake was half was termed K_U_. The K_U_ for cadmium with and without the presence of 100 μM went from 127 ± 12 μM to 21 ± 3.7 μM (*p* < 0.01). The K_U_ for manganese was almost 8 times higher at 945 ± 84 μM (*p* < 0.01) (Fig. 3d). Therefore, the main mechanism of transport for SMF1 preferred manganese and the uptake of cadmium was inferred to be due to transport leakiness.

### Screening pipeline to engineer metal specific transporters

**Fig. 4:**
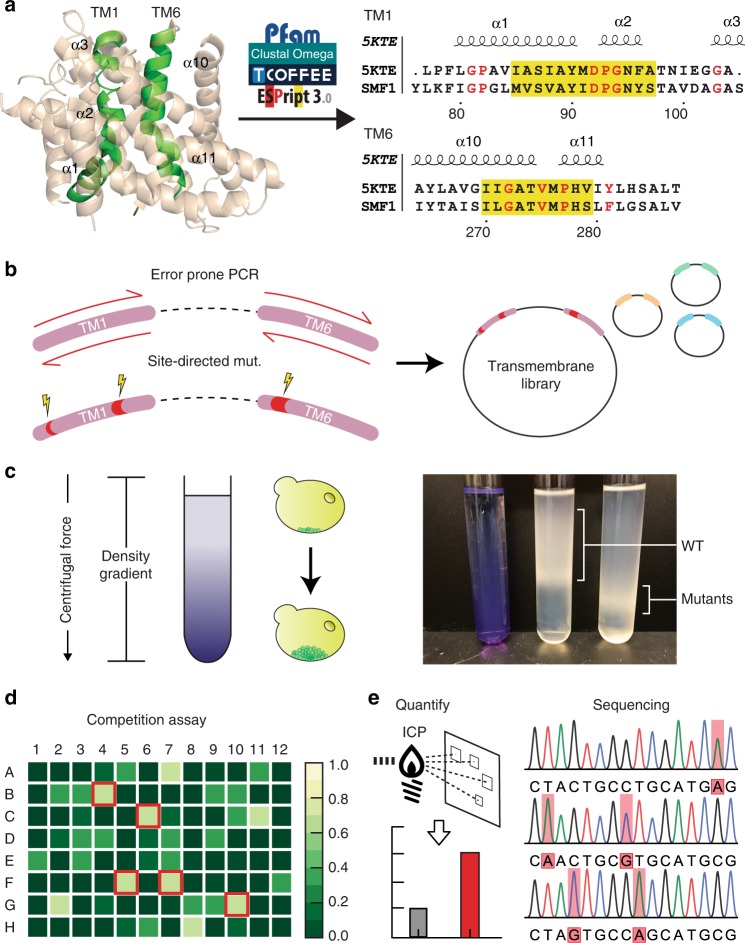
A developed high throughput screen to systematically engineer selective metal transporters. **a** Pfam protein database and clustering services such as ClustalΩ, TCoffee, and ESpript3 were used to align SMF1 with referenced protein crystal structure 5KTE^33^. Through literature searches and multi-alignments, transmembrane 1 and 6 (TM1, 6) were found to be the most significant regions for mutagenesis. The alignment comparing 5KTE with SMF1 shows the TM1 and TM6 region, where yellow highlights indicate conserved regions, and red text indicate highly conserved residues (similarity score > 0.7). **b** Mutations cited to enhance or decrease metal transport were selectively mutated using site-directed mutagenesis. Libraries were then generated on top of these mutations through error-prone PCR. **c** An initial screen was performed through rate-zonal density gradient centrifugation. **d**, **e** Fractionated layers were plated, picked, and assayed for metal uptake. A competition assay of the desired metal versus the native metal (e.g., manganese) was performed calorimetrically (**d**). Wells with the least amount of native metal uptake (highest signal) were selected and (**e**) quantitatively measured for metal uptake using ICP. Mutations were sequenced and reintroduced in the pipeline to generate better performing mutants

Crystal structures and literature on Nramp structure-to-function was used to semi-rationally build libraries to create two variants of SMF1. The first variant was a more specific cadmium transporter, and the other was a strontium transporter for potential application in radioactive Sr^90^ remediation. The crystal structures of SMF1 homologs *D. radiodurans* (DraNramp) and *S. capitis* (ScaDMT) were used to narrow down transmembrane domains (TM) fundamental for metal recognition and transport^33,34,36,38,43^. Specifically, TM regions 1, 4, and 6 in the Nramp family were identified to confer metal selectivity and movement^33,38^. Without a crystal structure for SMF1, the specific TM regions had to be inferred from known structures or through multi-alignments of conserved regions. Multi-alignment of SMF1 protein sequence against a Pfam database of homologous Nramps including DraNramp and ScaDMT revealed region 76–105, 180–200, and 264–287 to represent TM1, 4, and 6, respectively, based on the highest degree of conservation when compared to TM regions in the aligned homologs (Fig. 4a; Supplementary Fig. 10).

More so, previous work in Nramp mechanistic function showed that mutation M276 in SMF1 (discovered as M230 in DraNramp) conferred metal selectivity^34^. Outside crystallographic observations, it was empirically shown that mutating TM4 region G189 (discovered as G153 in DraNramp, or G185 in DMT1)^33,43^ into an arginine exposes a calcium entryway, which was hypothesized to also transport similar group II elements like strontium (Supplementary Fig. 10).

Mutating M276C and separately G189R and M276A were performed on SMF1 prior to generating libraries for cadmium and strontium screening, respectively. Given these base mutations, error-prone PCR was done sequentially on TM1 and TM6 to generate libraries (Fig. 4b) which were then transformed into BCT strains. Creating the cadmium and strontium mutant were performed in parallel, where separate libraries were screened for cadmium or strontium uptake. During screening, libraries were subjected to either 100 *μ*M cadmium or strontium similar to previous metal uptake experiments. Libraries were then screened based on an increase in mass as an indirect measurement for metal uptake. Mutants with higher metal content were fractionated using rate-zonal density gradient centrifugation (Fig. 4c; Supplementary Fig. 11). Rate-zonal, rather than isopynic density gradient centrifugation was used to fractionate cells based on changes in mass, rather than equilibrium density, as previous studies have shown that yeast maintain a relatively constant density despite external influences^44^. More so, our results showed the greatest segregation using rate-zonal density gradient centrifugation. Cells migrating the furthest were isolated, plated, and picked for colonies for a more focused metal assay. Cells were subjected to a competition assay with cadmium or strontium with 100 μM manganese in a 96 well format. A colorimetric assay specific to manganese was performed on the supernatant, where wells with the highest intensity (high manganese content; low manganese uptake) corresponded with mutants with low manganese preference (Fig. 4d). A select number of mutants were then chosen for quantitative metal uptake measurement using ICP, then sequenced, and later re-introduced into the mutagenesis/screening pipeline (Fig. 4e). 4–5 rounds of screening were performed to generate a cadmium and strontium mutant.

### Creating a SMF1 transporter specific to cadmium or strontium

**Fig. 5:**
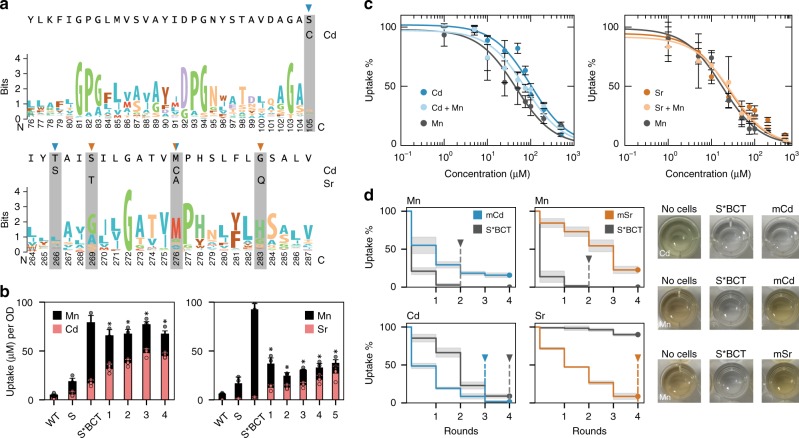
Creation of a cadmium and strontium metal transporter after 4–5 rounds of screens. **a** Weblogos of SMF1 TM1,6 from Nramp multi-alignment are displayed, with cadmium and strontium mutations highlighted. Cadmium mutants had S105C, T266S, and M276C. Strontium mutants had G189R, S269T, M276A, and G283Q. **b** Total metal uptake of 100 μM cadmium and manganese were measured to assess manganese interference. Cadmium mutant labeling corresponds to 1 = M276C, 2 = M276C + S105C, 3 = M276C + T266S, and 4 = M276C + S105C + T266S. Strontium mutant labeling corresponds to 1 = M276A, 2 = M276A + G189R, 3 = M276A + G189R + S269T, 4 = M276A + G189R + G283Q, and 5 = M276A + G189R + S269T + G283Q. Strain background for all mutants were BCT. Asterisk above bar charts represent significant changes in both Cd and Mn uptake compared to un-mutated S*BCT (*p* < 0.05). **c** Titration curves of fully mutated cadmium and strontium transporters in strain BCT were performed for Cd or Sr, respectively, with or without 100 μM Mn; *x*-axis represents the concentration of either Cd, Sr, or Cd, Sr, with Mn. **d** Sequential uptake experiments, up to 4 rounds, were performed to measure the amount of iterations required for complete elimination of 100 μM cadmium or strontium in a mixture of 100 μM manganese. Images on the right are colorimetric detection of cadmium and manganese (there are no available colorimetric assays for strontium at this concentration) showing selective preference for cadmium (no coloration) against native metal manganese (darkened well). For all data, the mean ± s.d. of three replicates are shown. The source data underlying Fig. 5c are provided as a Source Data file

The SMF1 mutant with the highest cadmium specificity (denoted as mCd) contained mutations S105C, M276C, and S269T; whereas the SMF1 mutant with the most selectivity for strontium (denoted as mSr) contained mutations G189R, T266S, M276C, and G283Q (Fig. 5a). To test the contributions of each mutation, SMF1* was systematically mutated at each of the changed residues to reveal their significance and effect on SMF1 expression and function. Many of the mutations on mCd and mSr were located on TM6 rather than TM1, which supports past observations of the highly sensitive permeation region in the first alpha-helix segment of TM1 (Supplementary Fig. 12). In addition, rounds of mutations leading to mCd and mSr did not significantly change expression levels (Supplementary Fig. 13).

Supporting Bozzi’s et. al. work, M276 plays a critical role in metal selectivity^34^. Changing the methionine to cysteine doubled cadmium uptake while halving manganese uptake (Fig. 5b) (*p* < 0.05). Whereas changing the methionine into alanine, and subsequently changing G189 into arginine enhanced strontium uptake while dramatically reducing uptake of manganese by almost 8-fold (*p* < 0.01) (Fig. 5b). These modifications, and each subsequent change, reduced Mn uptake while increasing uptake of Cd or Sr for mCd and mSr, respectively (Fig. 5b). It should be noted that these mutations could instead impede Mn uptake allowing increased permissiveness of Cd and Sr transport, rather than strictly increasing sensitivity for Cd or Sr; a subtle yet important distinction. However, in either case, the goal of improving Cd or Sr uptake is shown for mCd and mSr, respectively.

A titration experiment of cadmium and strontium with mCd and mSr, respectively, in the background of 100 μM manganese was performed to determine their new K_U_’s. For mCd the K_U_ for manganese dropped by 40-fold to 26.2 ± 7.6 (*p* < 0.01), whereas the K_U_ for cadmium went from 100 ± 3.2 without manganese to 75.8 ± 10.3 in the presence of manganese, a reduction by less than 25% (*p* < 0.05) in comparison to the 5-fold decrease with the non-mutated version (*p* < 0.01) (Fig. 3d; Fig. 5c). Similarly, for mSr, the K_U_ for manganese dropped to 17.9 ± 1.6 (*p* < 0.01) whereas the K_U_ for strontium was 26.8 ± 5.7 and remained constant at 27.1 ± 11 in the presence of manganese.

The improved preference for cadmium and strontium uptake was more obvious when performing iterative rounds of metal uptake. When comparing uptake of cadmium or strontium in the presence of manganese for mCd and mSr against the un-mutated S*, it took two rounds to fully remove manganese in the un-engineered case, while there still remained >10% manganese after 4 rounds for mCd and mSr which showed a significant reduction in manganese uptake (*p* < 0.01) (Fig. 5d). However, when measuring cadmium or strontium uptake, mCd completely removed cadmium after 3 rounds, while the un-engineered strain required 4 because of manganese uptake inhibition. For mSr, strontium incrementally decreased after each round without signs of manganese inhibition. After 4 rounds strontium levels reached below 10%, while the un-engineered strain had >80% strontium remaining (*p* < 0.01) signifying a significant change in metal preference from manganese to strontium.

## Discussion

This work demonstrated that yeast can be engineered to hyperaccumulate metals by overexpressing and evolving native metal transporters and engineering mechanisms for metal detoxification. The main design requirements for yeast hyperaccumulation are: (1) overexpression and engineered hyperactive membrane transporter activity, (2) overexpression of vacuole transporters for metal compartmentalization, and (3) enhanced metal tolerance. Co-expression of a cell membrane transporter (SMF1) and a vacuole metal transporter (CCC1), enhanced metal uptake of manganese and cadmium by more than 10-fold, exceeding their plant hyperaccumulating threshold of 10 mg/gDW and 0.1 mg/gDW, respectively. In addition, simultaneous expression of CCC1 and plant phytochelatin synthase TaPCS1 rescued yeast survival in the presence of 100 μM cadmium. In order to improve metal selectively against the preferred manganese substrate, and more towards cadmium or strontium, information from crystallographic and empirical observations from Nramp point mutations were utilized to strategically engineer relevant SMF1 transmembrane domains. Semi-rational mutagenesis of SMF1 combined with a screening pipeline based on mass changes using rate-zonal centrifugation generated SMF1 variants with either cadmium or strontium preference and more than 10-fold reduction in manganese selectivity.

Actual application of these yeast strains in real-world settings would require another layer of technological development, such as a container or cartridge to secure yeast in a controllable unit. Fortunately, these technologies exist, such as yeast packaging, freeze-drying, and delivery which are routine technologies found in the consumer market. A potential concept would be to grow and store yeast in commercial filter-like cartridges where they can be housed in filtering units with size-exclusion cutoffs to prevent yeast leakage back into the purified waters. An additional layer of safety is to genetically modify these yeast with kill switches, or a metabolic reliance on a controlled nutrient such that removal from these containers will result in cell death^45^.

There are yet many more handles that offer better control over metal hyperaccumulation. Expression levels of membrane metal transporters, specifically SMF1, can be enhanced by performing additional ubiquitin associated lysine mutations, deleting specific proteases such as PEP4^39,40^, or integrating multiple copies into the genome with inducible or constitutive expression. Uptake could be further enhanced by trafficking metals into other organelles such as the mitochondria, ER, or Golgi which themselves harbor multiple metal transpoters^26^. The same transporter screening pipeline developed in this work could also be used to engineer organelle metal transporters, such as CCC1, to further increase metal uptake and specificity in conjunction with a surface metal transporter like SMF1. A complementary approach, which is currently being investigated, is whether the deletion of metal exporters could improve metal retention and enhance overall metal accumulation. It may be a promising strategy to delete exporters from organelles, such as the Golgi, ER, mitochondria, etc. to gradually build up metal compartmentalization. This is a particularly interesting strategy if there is no good metal transporter candidate from these organelles, or if they are difficult to rationally engineer. Finally, yeast morphology could be altered to allow higher uptake capacity. Theoretically, the upper limit for metal uptake capacity is restricted to intracellular volume. If needed, organelle size, morphology, copy-number, and yeast size could be controlled with key genes such as VPH1 and VTCs^46,47^. Future work will assess whether increasing the physical volume of yeast or increasing the number of organelles such as the vacuole will lead to higher metal uptake capacity.

The major benefit of using transporters for metal hyperaccumulation and environmental remediation is the control over metal selectivity. Unlike current non-specific physicochemical techniques, biological transporters engineered for metal hyperaccumulation can distinguish less abundant yet more toxic metals over background elements. Biological systems have evolved a repertoire of transporters that can be leveraged for such selective metal uptake. This work demonstrated a focused study on SMF1; however, a similar approach using the same screening pipeline can be employed to other transporters mentioned earlier. Such engineering may be limited by the lack of structure-to-function knowledge and crystal structure availability for some transporters. However, advances in nanobody-aided crystallography, NMR, and cryoTEM may help elucidate transporter crystal structures for better mechanistic understanding^33,38^.

There are other areas in which yeast hyperaccumulators can have potential real-world applications. Given the customizability of yeast and methods proposed here to engineer metal selectivity, there is a possibility to design yeast strains by demand. Certain geographic areas suffer from specific metal contamination because of specific industries, for example areas in Bangladesh and India suffer from arsenic and chromium poisoning due to the textile industry^2,3^. Therefore, yeast could be tailored to selectively capture and remediate arsenic and chromium from their soils. The second application is to recycle, or mine out heavy metals back from solutions. Waste typically contains a mix of metals, making it extremely difficult to process and especially difficult to extract and re-capture precious metals. With this yeast-based approach it may be possible to not only remediate waste, but also to extract, concentrate, and store removed metals in yeast for mining purposes. Therefore, if a certain mixture contains *X* number of metals of interest, it would be possible to design *X* number of strains to individually target and mine back those metals. Using yeast as a mechanism for metal removal, as well as mining and recycling can close the loop between manufacturing, use, and disposal. Therefore, rather than providing a palliative solution for the waste management crisis, yeast could be an integral tool for waste treatment processes and recycling.

## Methods

### Yeast strain and culture

Yeast strain W303*α* was obtained from the Amon Lab at MIT. Synthetically defined dropout media (SD) was made by combing 1.7 g/L yeast nitrogen base without amino acid and ammonium sulfate (YNB) (Fischer), 5 g/L ammonium sulfate (Sigma), 0.6 g CSM-HIS-LEU-TRP-URA powder (MPBio), 20 g/L glucose (Sigma), and 10 mL/L of 100× adenine hemisulfate stock (1 g/L) (Sigma). 100× stocks of His (5 g/L), Leu (10 g/L), Trp (10 g/L), and Ura (2 g/L) (Sigma) were made in ddH_2_O and filtered sterilized before supplementing cultures. Alternatively, complete synthetically defined media (CSM) was made with the above ingredients but with 0.79 g/L CSM mix (MPBio). YPD was made with 20 g/L peptone, 20 g/L glucose and 10 g/L yeast extract. CSM/SD-R media was made by replacing 20 g/L glucose with raffinose (VWR). CSM/SD-G media was made by replacing 20 g/L glucose with 20 g/L galactose and 20 g/L raffinose. Solutions were stirred and filter sterilized through a 0.22 μm filter top (EMD). Agar plates were made by adding 20 g/L BactoAgar (Fisher) and autoclaving before pouring.

### Isolating genomic DNA

Cultures were grown overnight in their appropriate drop-out media. 500 μL of cells were then transferred and pelleted at 900×*g* for 3 min Cells were then resuspended in 250 μL DNA breakage buffer containing 2% Triton X-100, 1% SDS, 0.1 M NaCl, 10 mM Tris-HCl, 0.5 M EDTA (Sigma) in 100 mL ddH_2_O. Approximately 1:1 of acid-washed 420–600 μm glass beads (Sigma) to cell pellet were added to the tubes. 250 μL phenol/chloroform/isoamyl alcohol (25:24:1; Sigma) was then layered on top. Tubes were bead beaten for 5 min and spun down at 14,000 × *g* for 5 min at 4 °C. The aqueous layer was then removed and added to 1 mL of ice-cold 100% EtOH (VWR) and spun down at 14,000 × *g* for 5 min at 4 °C. EtOH was aspirated leaving behind precipitated DNA which was then dried at room temp for 30 min Cells were then resuspended in TE buffer (Sigma) for downstream cloning.

### Cloning metal transporters

Sequences were acquired from the Yeast Genome Database (www.yeastgenome.org) or through NCBI (https://www.ncbi.nlm.nih.gov/gene). All cloning steps were first simulated with Snapgene. All enzymes including the commercial non-trademarked Gibson assembly master mix, HiFi, were purchased from NEB. All references to Gibson assembly used the HiFi master mix. Between each PCR step, products were cleaned using the Wizard SV Gel and PCR CleanUp Kit (Promega). The pYES2/CT (Invitrogen) was used as the plasmid backbone for gene expression. The pYES2/CT vector was modified by inserting a stop codon after the V5 tag to eliminate expression of the C’ terminus 6xHis tag. All sequences were confirmed by Sanger sequencing using Quintara Bio.

Metal transporters, CTR1, CTR3, FET4, FTR1, SMF1, SMF2, ZRT1, and ZRT2 were amplified from genomic W303*α* DNA using PCR and ligated into pYES2/CT via restriction cloning. Forward and reverse primers (Supplementary Table 2) of the metal transporter genes were flanked with the KpnI and XhoI restriction sites and trailed by *TAAGCA* junk DNA to enable efficient restriction cleavage. All genes were followed by the V5 tag native to the pYES2/CT vector.

Permeases Pho84, Pho87, Pho89, Sul1, Sul2 were amplified from genomic W303*α* DNA using PCR and Gibson assembled into the pYES2/CT vector. During assembly overhangs contained a HA tag to replace the V5 tag of the pYES2/CT vector (Supplementary Table 3).

Nrat1 protein sequence was retrieved from Uniprot (www.uniprot.org), codon optimized, and synthesized using Genscript. Nrat1 was Gibson assembled into pYES2/CT and immediately followed by the V5 tag (Supplementary Table 4).

CCC1, COT1, ZRC1, and SMF3 were assembled into a modified pYES2/CT vector. The pYES2/CT original URA marker was replaced with a LEU marker taken from the pRS305 vector. The CCC1 gene was first cloned using restriction sites SacI and BamHI. The V5 tag was replaced with a Flag tag by introducing the appropriate PCR primer overhangs. The remaining vacuole transporter genes COT1, ZRC1 and SMF3 were created by replacing the CCC1 via Gibson assembly (Supplementary Table 5).

### Engineering S*BCT

A mutated version of SMF1 was performed by mutagenzing the K33,34 region, *AAGAAA*, into arginines, *AGGAGA*, using QuikChange site-directed mutagenesis (Agilent) (Supplementary Table 6). The BSD2 ubiquitin ligase was deleted amplifying the HIS cassette using PCR from pRS303 containing 40 bp overlap with the genomic BSD2 region and transformed following the transformation protocol described below (Supplementary Table 6).

TaPCS1 was ordered from Addgene (#49767; deposited by the Julian Schroeder Lab) and inserted into the pD1235 vector (ATUM) via Gibson assembly. The gene along with the TRP marker was amplified with PCR with 40 bp overlap over the *trp1–1* region of the W303 strain and transformed for genomic integration (Supplementary Table 6).

pYES2/CT with CCC1 was modified to allow proper integration into the yeast genome. The vector was reorganized to have the LEU marker downstream of the gene, the swap being made via Gibson assembly. CCC1 along with the LEU cassette was amplified with PCR with 40 bp overlap over the *leu2–3* region of the W303 strain and transformed for genomic integration (Supplementary Table 6).

### Identifying and mutagenizing SMF1 TM regions

Pfam (https://pfam.xfam.org/) was used to curate the representative proteomes from the Nramp family and were compared using TCoffee’s transmembrane multi-alignment algorithm (http://tcoffee.crg.cat/). To check the accuracy of this tool the same dataset was aligned using Clustal Omega (https://www.ebi.ac.uk/services/teams/clustal-omega), which showed similar results. The resultant multi-aligned file was visualized using ESPript (http://espript.ibcp.fr/ESPript/ESPript/) with reference sequence taken from PDB entry 5KTE (https://www.rcsb.org/structure/5tke) to help indicate regions with secondary structure. Red highlighted amino acids indicate highly conserved regions with similarity scores >0.7. All other amino acids are colored black. Visualized alignments identified transmembrane regions on SMF1, and mapped residues G153 and M230 found in 5KTE to G189 and M276 on SMF1, respectively. Sequence usage of the Nramp family was also visualized using WebLogo (https://weblogo.berkeley.edu/logo.cgi) and mapped onto TM1,4 and 6 of SMF1 to qualitatively understand the significance of mutated regions during screening.

Libraries of SMF1 were generated using primers flanking TM1 and 6 which were then used with Agilent’s GenemorphII EZClone mutagenesis kit (Supplementary Table 7). Site-directed mutagenesis primers were created using Agilent’s primer design webservice (www.agilent.com/genomics/qcpd) and mutations were introduced using Agilent’s Quikchange lightning or multi-site mutagenesis kits (Supplementary Table 7).

### Transformations

Plasmid constructions were performed in NEB*α* competent cells (NEB) and transformed following NEB’s protocol. Yeast transformations were performed with the Frozen-EZ Yeast Transformation Kit II (Zymo Research). The protocol was modified slightly for integrated constructs. Transformed cells were first plated onto YPAD plates and grown for 1 day. Plates were replica-plated on their respective SD drop-out and grown for an additional 1–2 days. A total of 4–8 colonies were then picked, grown overnight, and smash and grabbed to isolate their genomic DNA. DNA was then amplified using PCR with primers flanking the integrated area of interest and ran on a gel to verify proper integration.

### Correlating OD_600_ to culture dry weight

Wild-type W303 were grown and diluted to various culture densities ranging from 0.1–2 OD_600_ in 500 mL. Cells were pelleted and washed 3× in ddH_2_O. 50 mL conical tubes were pre-weighed on an analytical balance with microgram resolution. Cells were transferred into these tubes, pelleted, and resuspended in 1 mL of H_2_O. Tubes were then dipped and snap-freezed in liquid nitrogen. Tubes were then capped with a porous cloth and fitted into a lyophilization chamber (VirTis) and lyophilized for 48 h. Tubes with cells were weighed with weight of the tube subtracted to calculate cell dry weight (DW). Mass of cells per volume (*y*-axis) was plotted against measured OD_600_ (*x*-axis) giving a ratio between OD and culture dry weight per culture volume. OD to culture dry weight correlation factor was used to convert ICP results with units of μM to milligram of metal removed per gram of yeast dry weight (mg/gDW) for each strain mentioned in the results.

### Metal uptake analysis using inductive coupled plasma

Liquid stocks of copper (II) chloride, zinc chloride, iron (II) chloride, manganese (II) chloride, cadmium nitrate, and strontium chloride (Sigma) were made at 100 mM in ddH_2_O and filtered through a 0.22 μm filter. Colonies were streaked on SD agar plates, picked, and inoculated in SD-R media with the appropriate supplemented amino acids. Overnights were diluted 1:10 in SD-R and grown for 4 h. Cells were then pelleted and resuspended in SD-G media for induction overnight.

To prepare cells for metal uptake analysis, cells induced with SD-G were diluted to 1 OD_600_ in fresh SD-G and spiked with 100 μM metal and incubated for 4 h at 30 °C. After 4 h of metal incubation OD_600_ was measured again to consider any changes in culture density. Afterwards, cells were pelleted and supernatant collected for metal analysis. Metal concentrations were measured in an inductive coupled plasma (ICP) Agilent ICP-AES 5100 instrument following standard operating procedures provided by the Center of Material Science facility at MIT. Metal standards were made from ICP-grade stock solutions purchased from Fluka and diluted in a 2–3% HNO_3_ matrix in CSM buffer. After ICP analysis metal uptake was calculated by subtracting 100 μM (original metal concentration) by the metal concentration measured in the supernatant. The value was then divided by the OD_600_ measurement to give units of μM/OD in order to equally compare uptake levels between strains and metals. Units were further converted to mass of metal removed per cell dry weight to help compare against literature values which report hyperaccumulation values in units of mass (mg/gDW). The conversion of μM to mg/gDW required multiplying the molarity of metal removed by the molecular weight of the metal, and converting the culture OD to gram of dry weight using the ratio derived in the OD_600_ to culture dry weight analysis described above.

To control for non-specific metal binding onto the cell wall, a control sample containing a wild-type W303*α* (WT) strain was also spiked with 100 μM metal and processed similarly to account for non-specific uptake for in a non-expressing strain. Another sample containing no cells was also spiked with 100 μM metal to test for non-specific metal binding on to the test tube and equipment. In addition, a more rigorous test for non-specific binding was performed by washing cells after metal uptake and measuring the metal content in the wash buffer. After metal uptake experiments, cells were washed once with ddH_2_O to remove any residual liquid, as not all the liquid was removed for ICP analysis. Afterwards, the cells were washed once more with ddH_2_O to the original volume and gently incubated for 3 min Cells were spun down, and supernatant measured for metal content. Afterwards, cells were washed another time in an EDTA buffer (10 mM Tris with 1 mM EDTA, pH 7.4) to the original volume and incubated for 3 min Cells were then pelleted, supernatant removed, and measured for metal content again.

Metal uptake titration experiments were performed following the same method but using different metal concentrations ranging from 1 μM to 100 μM or 1 mM. Metal uptake was normalized to percent uptake with respects to the original metal concentration added. The concentration at which 50% of metal was removed was termed K_U_. For interference experiments, titrations against the desired metal (cadmium or strontium) was performed in the presence of constant 100 μM manganese.

Iterative metal uptake experiments were performed by taking the supernatant of a previous metal uptake experiment, and transferring the supernatant directly into a freshly induced culture normalized to 1 OD_600_. Uptake was performed for 4 h, and supernatant transferred iteratively to a fresh new culture up to 4 times. At each iteration the supernatant was sampled and measured using ICP to calculate the metal uptake per round.

### Staining and microscopy

Transporter expression was measured using immunohistochemistry. Cells were induced following the procedure mentioned above and fixed with 3.7% paraformaldehyde (EMS) at 0.5 OD_600_ for 30 min at room temperature_._ Cells were pelleted at 900×*g* and washed 3× in 1.2 M sorbitol-citrate buffer (Sigma) before resuspending in the same buffer with 1:100 dilution of 100T Zymolyase (Zymo) and incubated at 30 °C for 30 min to 1 h. Cells were pelleted and washed 3× in PBS + 1% BSA before settling on poly-lysine treated 8 well chamber slides (Lab-Tek). Cells were gently permeabilized with 0.1% Tween-20 (Sigma) in PBS + 1% BSA on ice for 5 min Cells were then stained with the appropriate primary antibody against V5 (2F11F7; Thermo), HA (5B1D10; Thermo), or Flag tag (FG4R or PA1-984B; Thermo). V5 and HA antibodies were diluted 1:500, while the Flag antibodies were diluted 1:1000. After 1 h at 4 °C, cells were washed 3× in PBS + 1% BSA, and stained with the appropriate secondary antibody conjugated to AlexaFluor488 (A-11001; Thermo) or 647 (A-21245; Thermo) diluted to 1:2000. DAPI at 5 μg/mL (Thermo) in PBS was used to stain nuclei for 3–5 min Cells were washed and aspirated before removing the wells. A 24 × 50 mm coverslip was placed gently on the slide with 60% glycerol in PBS as the mounting media. Nail polish was used to seal the edges and slides were imaged on an AxioPlan2 within 24 h.

In all experiments, a non-expressing WT control was stained in parallel to measure non-specific antibody binding and autofluorescence. The same primary and secondary antibodies (V5, HA, Flag tag, etc.), and staining conditions were performed similarly with the experimental samples.

### Quantifying membrane expression using flow cytometry

SMF1 variants (S, S^*^, SB, S^*^B, iS^*^B) were stained with antibodies following the same steps in the staining and microscopy methods. Cells were diluted to 0.1 OD_600_ in PBS + 1% BSA and measured on a BD FACS Celesta or LSR II following standard operating procedures provided by the Koch Flow Cytometry Core. Yeast cell gating strategy followed: FSC-A and SSC-A was used to gate on cells. FSC-W and FSC-H was used to gate vertically oriented single cells (vertical singlets). SSC-W and SSC-H was used to gate horizontally oriented single cells (horizontal singlets). After gating on these 3 plots, single cells were measured based on fluorescence (Supplementary Fig. 1^4^). Cell counts were plotted against binned fluorescent intensity (*x*-axis) creating a population distribution histogram of fluorescence (*y*-axis). The mean fluorescent intensity weighted by cell count was used to quantitatively compare fluorescent intensity (i.e., expression) against metal uptake measured by ICP for those strains.

### Cell culture density measurements and viability assays

OD_600_ measurements were performed using 2 mL non-frosted cuvettes and a table-top DU800 Beckman Coulter spectrophotometer measured at 600 nm. OD_600_ values were used to divide metal uptake values measured by ICP to normalize for culture density.

Cell viability was measured at different metal concentrations ranging from 1 μM to 100 μM. Cultures were grown overnight and then diluted to <0.1 OD_600_. Cultures were aliquoted to a total volume of 100 μL and spiked with varying metal concentrations. Cultures were placed in a 96 well U-bottom plate (Cellstar) and shaken in a BioTek Synergy 2 plate reader held at 30 °C for 24–36 h. Growth rates were calculated by finding the maximum slope in the growth curve.

A live-dead assay was also performed to analyze cell viability by calculating the ratio of live to dead cells after metal uptake experiments. Cells after metal uptake experiments were resuspended in culture media and dyed with a live-dead fluorescent indicator (Thermo). A positive control of freshly grown cells, and a negative control of cells heated to 70 °C for 15 min, were used to gate the live and dead cell populations, respectively. Counts within those gates were used to calculate ratio of live cells after metal uptake. Cells were analyzed under the FITC and PE channels of an LSR II flow cytometer.

### Manganese assay

The manganese colorimetric detection Hach kit was modified to fit a 96-well format. Fifty microliter of sample was added to 50 μL of 2× ascorbic acid provided by the kit. Then 5 μL of the cyanide and PANI reagent were used to detect manganese given a colorimetric change from yellow to red. Wells were measured at 560 nm. Cyanide was disposed of using guidelines approved by MIT EH&S.

### Screening transporter libraries

Percoll (Sigma) buffered with 1.5 M NaCl was used to make density gradients. A Pharmacia LKB Pump P-1 peristaltic pump joined to a gradient maker (GE) was used to make Percoll gradients. Gradients were formed in Greiner 16 × 100 mm round bottom polystyrene tubes (Sigma) which were first hydrophobically coated with Sigmacote (Sigma). A purple dye was used as a control to visually inspect consistency of gradient formation per batch.

Libraries were transformed into yeast and plated. Single colonies were pooled together using a scraper (Corning) into 10 mL of SD-R with the appropriate amino acids. Cells were grown for 12 h before being diluted into 50 mL of SD-R for 4 h. Cells were then pelleted and resuspended in SD-G with the appropriate amino acids for induction overnight. Induced culture was then diluted to 1 OD_600_ into multiple 10 mL SD-G media with spiked 100 μM of cadmium or strontium. Cultures were grown for 4 h before washing and resuspending in 150 mM NaCl. Cells, percoll gradient, and an Eppendorf 5804-R swinging bucket centrifuge were chilled to 16 °C before spinning. Settings for acceleration and braking were set to 0. Cells were gently layered onto the gradient and spun in increments of 5 min at 100 × *g*. A total of 3–4 spins were sufficient to observe segregation of cells which signified a fractionation of heavier cells due to metal uptake. Approximately a centimeter below the least visible band was collected and spun down at 1500 × *g* for 3 min before resuspending in SD with the appropriate amino acids. Cells were rescued for 1.5 h before plating. Collected cells were plated onto 2–3 plates giving approximately 10–100 colonies each.

After platting roughly 10–50 colonies were picked in 100 μL SD-R cultures in a 96-well format and induced following the same protocol as before. Cells were diluted to 1 OD_600_ and spiked with 100 μM cadmium or strontium with the addition of 100 μM manganese and shaken for 4 h. Plates were spun down at 900 × *g* for 3 min and the supernatant was diluted 1:10 in ddH_2_O and assayed using the modified manganese Hach detection kit described above. The top 4–6 wells with the highest readings (most manganese remaining) were selected and plated again. Selected colonies were then subjected to a more thorough metal uptake ICP experiment and sequenced before re-introduction into the screening pipeline.

### Mathematical analysis and plotting

Raw data were collected and stored as csv or excel file formats. Data were imported and analyzed with python using modules such as numpy, pandas, and scipy. Plots were graphed using matplotlib.

### Statistical analysis

Statistical parameters including the definitions and values of *n*, SDs, and/or SEs are reported in the figures and corresponding figure legends. When reporting significance, a two-tailed unpaired *t*-test was performed for all calculated *p*-values. The significance threshold was set to *p* < 0.05 for all experiments, or as specified in the text.

### Reporting summary

Further information on research design is available in the Nature Research Reporting Summary linked to this article.

## Supplementary information

Supplementary Information

Peer Review

Reporting summary

Source Data

## Data Availability

Data supporting the findings of this work are available within the paper and its Supplementary Information files. A reporting summary for this Article is available as a Supplementary Information file. The datasets generated and analyzed during the current study are available from the corresponding author upon request. The source data underlying Figs. 1b, 2b, 3a, b, d, and 5c, as well as Supplementary Figs. 2, 3, 7 and 9 are provided as a Source Data file.

## References

[1] Powell JT, Townsend TG, Zimmerman JB (2016). Estimates of solid waste disposal rates and reduction targets for landfill gas emissions. Nat. Clim. Change.

[2] Robinson T, McMullan G, Marchant R, Nigam P (2001). Remediation of dyes in textile effluent: a critical review on current treatment technologies with a proposed alternative. Bioresour. Technol..

[3] Manzoor S, Shah MH, Shaheen N, Khalique A, Jaffar M (2006). Multivariate analysis of trace metals in textile effluents in relation to soil and groundwater. J. Hazard. Mater..

[4] Sahoo SK (2016). Strontium-90 activity concentration in soil samples from the exclusion zone of the Fukushima daiichi nuclear power plant. Sci. Rep..

[5] Shutov, V. N., Bruk, G. Y., Balonov, M. I., Parkhomenko, V. I. & Pavlov, I. Y. In *The Chernobyl papers. Volume 1. Doses to the Soviet Population and Early Health Effects Studies* (eds Balonov, M. I. & Mervin, S. E.) 167–218 (Research Enterprises, 1993).

[6] Mangano JJ, Sherman JD (2011). Elevated in vivo strontium-90 from nuclear weapons test fallout among cancer decedents: a case-control study of deciduous teeth. Int. J. Health Serv..

[7] Fu F, Wang Q (2011). Removal of heavy metal ions from wastewaters: a review. J. Environ. Manag..

[8] Kurniawan TA, Chan GYS, Lo W-H, Babel S (2006). Physico–chemical treatment techniques for wastewater laden with heavy metals. Chem. Eng. J..

[9] Kumar Gupta V, Ali I, Saleh A, Nayak T, Agarwal A (2012). S. Chemical treatment technologies for waste-water recycling-an overview. RSC Adv..

[10] Marques AP, Rangel AO, Castro PM (2009). Remediation of heavy metal contaminated soils: Phytoremediation as a potentially promising clean-up technology. Crit. Rev. Environ. Sci. Technol..

[11] Singh A, Prasad SM (2015). Remediation of heavy metal contaminated ecosystem: An overview on technology advancement. Int. J. Environ. Sci. Technol..

[12] Prasad V, Narasimha M, Freitas, de O, Maria H (2003). Metal hyperaccumulation in plants: Biodiversity prospecting for phytoremediation technology. Electron. J. Biotechnol..

[13] Krämer U (2010). Metal hyperaccumulation in plants. Annu. Rev. Plant Biol..

[14] Branquinho C, Serrano HC, Pinto MJ, Martins-Loução MA (2007). Revisiting the plant hyperaccumulation criteria to rare plants and earth abundant elements. Environ. Pollut..

[15] Clemens S, Palmgren MG, Krämer U (2002). A long way ahead: understanding and engineering plant metal accumulation. Trends Plant Sci..

[16] Hall JL (2002). Cellular mechanisms for heavy metal detoxification and tolerance. J. Exp. Bot..

[17] Eapen S (2006). Phytoremediation of radiostrontium (90Sr) and radiocesium (137Cs) using giant milky weed (*Calotropis gigantea R.Br*.) plants. Chemosphere.

[18] Yang XE (2004). Cadmium tolerance and hyperaccumulation in a new Zn-hyperaccumulating plant species (*Sedum alfredii Hance*). Plant Soil.

[19] Sarma H (2011). Metal hyperaccumulation in plants: a review focusing on phytoremediation technology. J. Environ. Sci. Technol..

[20] Eapen S, D’Souza SF (2005). Prospects of genetic engineering of plants for phytoremediation of toxic metals. Biotechnol. Adv..

[21] Liu W, Yuan JS, Stewart CN (2013). Advanced genetic tools for plant biotechnology. Nat. Rev. Genet..

[22] Barth-Haas Group. Worldwide beer production, 2016. https://www.statista.com/statistics/270275/worldwide-beer-production/ (2018).

[23] BCC Research. Yeasts, yeast extracts, autolysates and related products: the global market. https://www.bccresearch.com/market-research/chemicals/yeast-yeast-extracts-autolysates-products-chm053c.html (2017).

[24] PR Newswire. Value of the yeast product market worldwide from 2016 to 2022 (in billions U.S. dollars). https://www.statista.com/statistics/728147/global-yeast-product-market-size/ (2017).

[25] André B (1995). An overview of membrane transport proteins in *Saccharomyces cerevisiae*. Yeast.

[26] Luk E, Jensen LT, Culotta VC (2003). The many highways for intracellular trafficking of metals. JBIC J. Biol. Inorg. Chem..

[27] Anthony Van Ho, Diane McVey Ward, Kaplan J (2002). Transition metal transport in yeast. Annu. Rev. Microbiol..

[28] Catarecha P (2007). A mutant of the Arabidopsis phosphate transporter PHT1;1 displays enhanced arsenic accumulation. Plant Cell.

[29] Pereira Y (2008). Chromate causes sulfur starvation in yeast. Toxicol. Sci..

[30] Zhao FJ, Ma JF, Meharg AA, McGrath SP (2009). Arsenic uptake and metabolism in plants. New Phytol..

[31] Shanker AK, Cervantes C, Loza-Tavera H, Avudainayagam S (2005). Chromium toxicity in plants. Environ. Int..

[32] Xia J, Yamaji N, Kasai T, Ma JF (2010). Plasma membrane-localized transporter for aluminum in rice. Proc. Natl Acad. Sci. USA.

[33] Bozzi AT (2016). Crystal structure and conformational change mechanism of a bacterial nramp-family divalent metal transporter. Structure.

[34] Bozzi AT (2016). Conserved methionine dictates substrate preference in Nramp-family divalent metal transporters. Proc. Natl Acad. Sci. USA.

[35] Ehrnstorfer IA, Manatschal C, Arnold FM, Laederach J, Dutzler R (2017). Structural and mechanistic basis of proton-coupled metal ion transport in the SLC11/NRAMP family. Nat. Commun..

[36] Courville P, Chaloupka R, Cellier MFM (2006). Recent progress in structure-function analyses of Nramp proton-dependent metal-ion transporters. Biochem. Cell Biol..

[37] Liu XF, Supek F, Nelson N, Culotta VC (1997). Negative control of heavy metal uptake by the *Saccharomyces cerevisiae* BSD2 Gene. J. Biol. Chem..

[38] Ehrnstorfer IA, Geertsma ER, Pardon E, Steyaert J, Dutzler R (2014). Crystal structure of a SLC11 (NRAMP) transporter reveals the basis for transition-metal ion transport. Nat. Struct. Mol. Biol..

[39] Nikko E, Pelham HRB (2008). Arrestin-like proteins mediate ubiquitination and endocytosis of the yeast metal transporter Smf1. EMBO Rep..

[40] Liu XF, Culotta VC (1999). Post-translation control of nramp metal transport in yeast. J. Biol. Chem..

[41] Jeyaprakash A, Welch JW, Fogel S (1991). Multicopy CUP1 plasmids enhance cadmium and copper resistance levels in yeast. Mol. Gen. Genet..

[42] Clemens S, Kim EJ, Neumann D, Schroeder JI (1999). Tolerance to toxic metals by a gene family of phytochelatin synthases from plants and yeast. EMBO J..

[43] Xu H, Jin J, DeFelice LJ, Andrews NC, Clapham DE (2004). A Spontaneous, recurrent mutation in divalent metal transporter-1 exposes a calcium entry pathway. PLoS Biol..

[44] Bryan AK, Goranov A, Amon A, Manalis SR (2010). Measurement of mass, density, and volume during the cell cycle of yeast. Proc. Natl Acad. Sci. USA.

[45] Chan CTY, Lee JW, Cameron DE, Bashor CJ, Collins JJ (2016). ‘Deadman’ and ‘Passcode’ microbial kill switches for bacterial containment. Nat. Chem. Biol..

[46] Desfougères Y, Neumann H, Mayer A (2016). Organelle size control-increasing vacuole content activates SNAREs to augment organelle volume through homotypic fusion. J. Cell Sci..

[47] Turner JJ, Ewald JC, Skotheim JM (2012). Cell size control in yeast. Curr. Biol..

