# Supplementary material for: Designing yeast as plant-like hyperaccumulators for heavy metals
Source: Nat Commun. 2019 Nov 8;10:5080. doi: 10.1038/s41467-019-13093-6 (PMC6841955; doi:10.1038/s41467-019-13093-6)
Supplement: Supplementary file 1 — Supplementary Information [file 41467_2019_13093_MOESM1_ESM.pdf]

Designing yeast as plant-like hyperaccumulators for heavy metals

Sun *et al.*

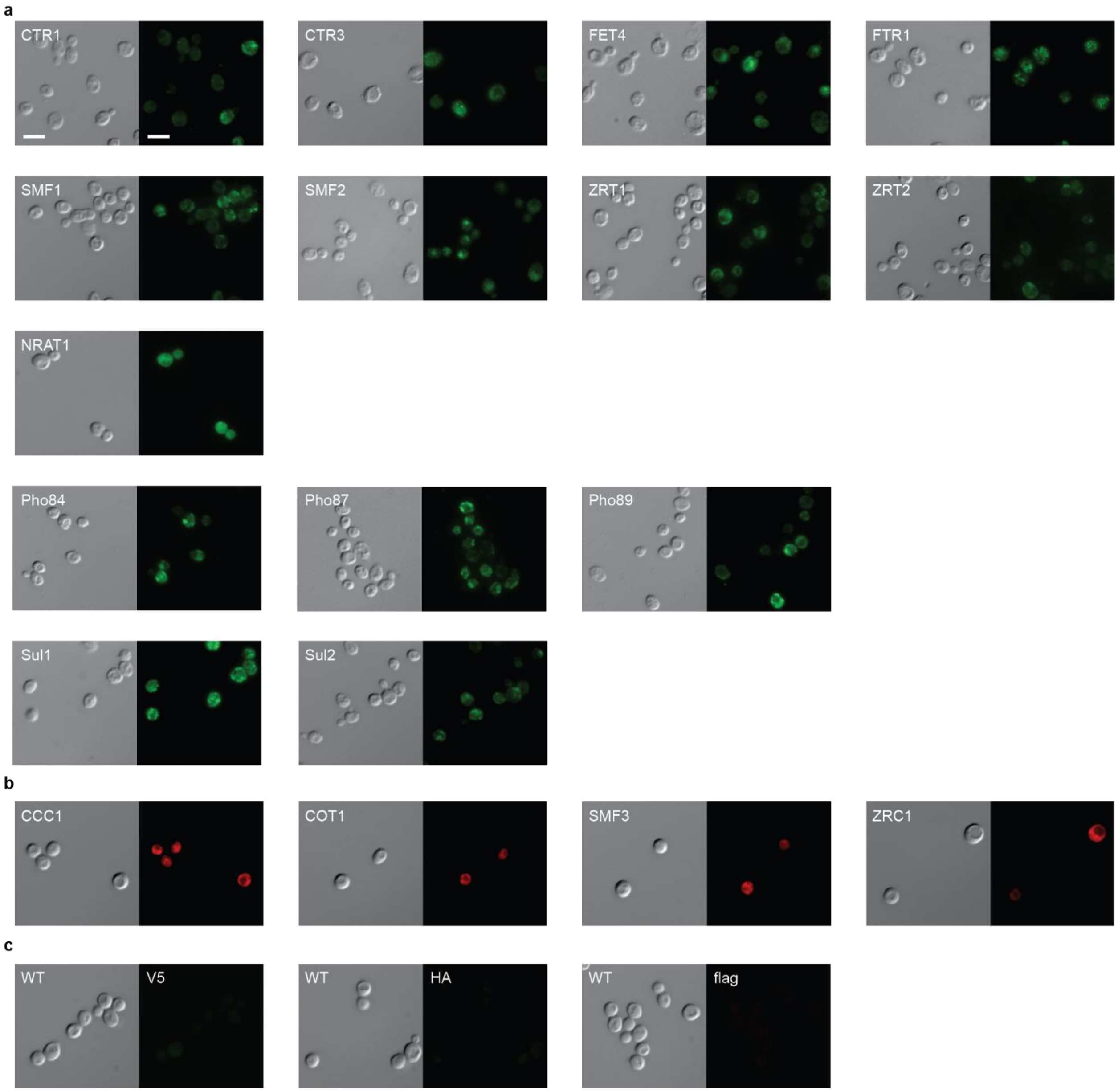

Supplementary Figure 1. Visualization of fluorescently stained membrane and vacuole transporters. a) Membrane transporters CTR1, CTR3, FET4, FTR1, SMF1, SMF2, ZRT1, ZRT2 and Nrat1 were fused with a C'-terminus V5 tag and stained. Pho84, Pho87, Pho89, and Sul1 and Sul2 were fused with a C'-terminus HA tag and stained. **b**) Vacuole transporters CCC1, COT1, SMF3, and ZRC1 were fused with a C'-terminus flag tag and stained. **c**) Negative controls of WT were stained with identical antibodies targeting V5, HA, and flag tag in parallel

with the transporters already described. No noticeable background fluorescence was observed. All tags were labelled with the appropriate primary and secondary antibodies conjugated with either an Alex488 (green; membrane) or Alex647(far red; vacuole) dye. Scale bars represent 5 μm for all images.

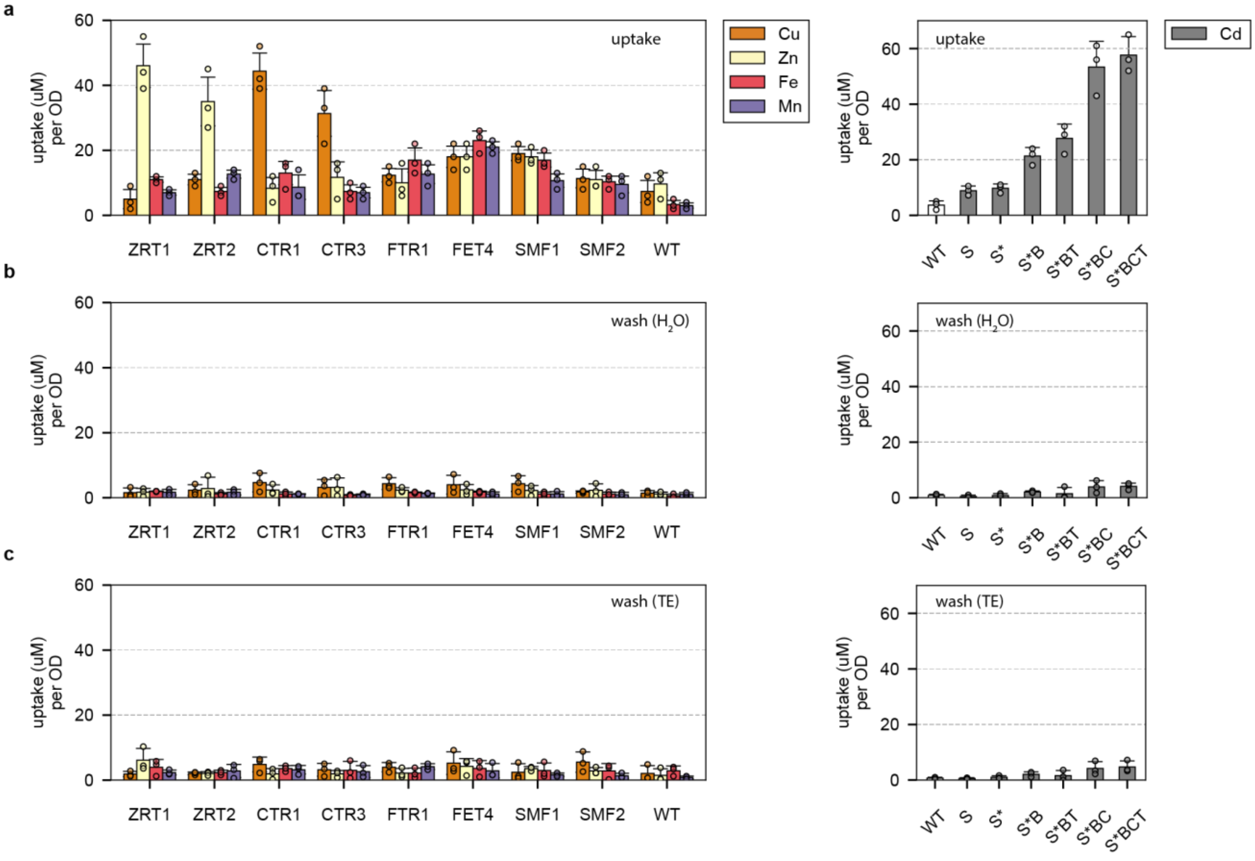

Supplementary Figure 2. Impact of non-specific metal binding during metal uptake experiments. a) Measurement of metal removed from a 100 μM metal uptake experiment (refer to Figure 1b and 3a). **b**) After a metal uptake experiment, cells were washed with ddH_2O and supernatant measured for freed metal. **c**) After the ddH_2O wash step, another wash step in a 1 mM EDTA buffer was similarly measured for freed metal. For all data, the mean ± s.d. of three replicates are shown. Source data are provided as a Source Data file.

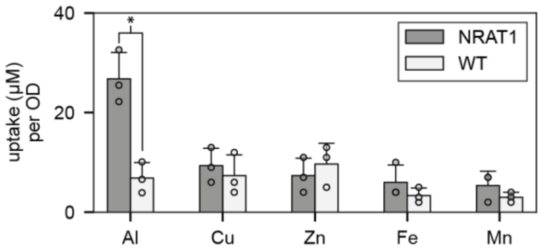

Supplementary Figure 3. Nrat1 uptake is selective for Al^{3+} ions. Metal uptake experiments for Nrat1 was performed with Al, Cu, Zn, Fe, and Mn and compared against a non-expressing WT strain. Asterisk above bar chart represents significant uptake when compared to WT ($p < .05$). For all data, the mean ± s.d. of three replicates are shown. Source data are provided as a Source Data file.

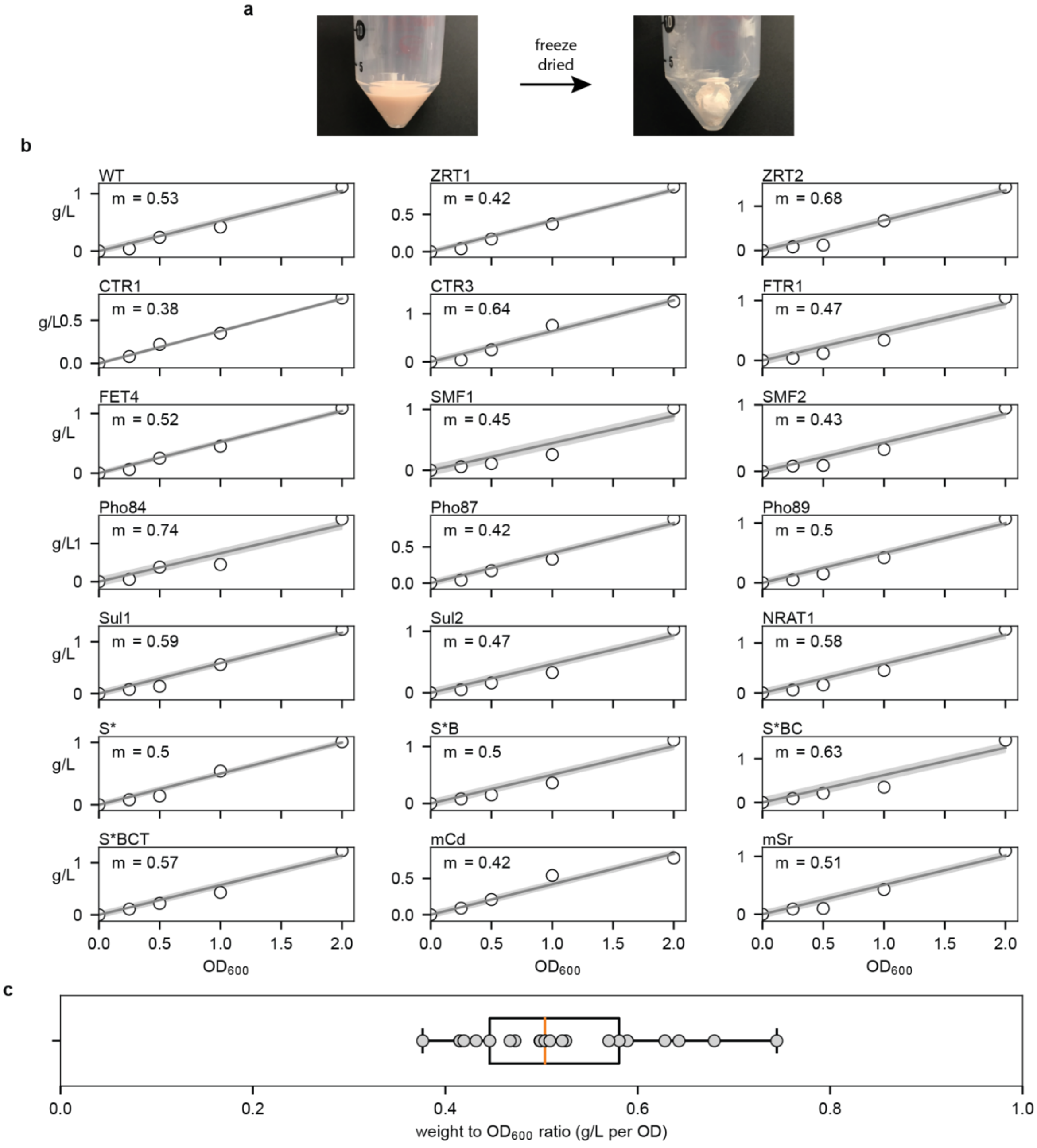

Supplementary Figure 4. Correlating culture optical density to grams of culture dry weight. **a**) Cells were grown, washed, pelleted, and freeze-dried to obtain culture dry weight per culture volume. Masses were weighed on a precision scale with hundredths of milligram resolution. **b**) A line of best fit with intercept at 0 was performed to obtain a correlation factor between optical density (OD_{600}) and gram of dry weight (gDW) per culture volume for each strain. **c**) A box plot

of all OD_{600} to gram dry weight correlation factors. On average the correlation factor was approximately 0.52 g/L per OD_{600}. For all data, the mean ± s.d. of three replicates are shown.

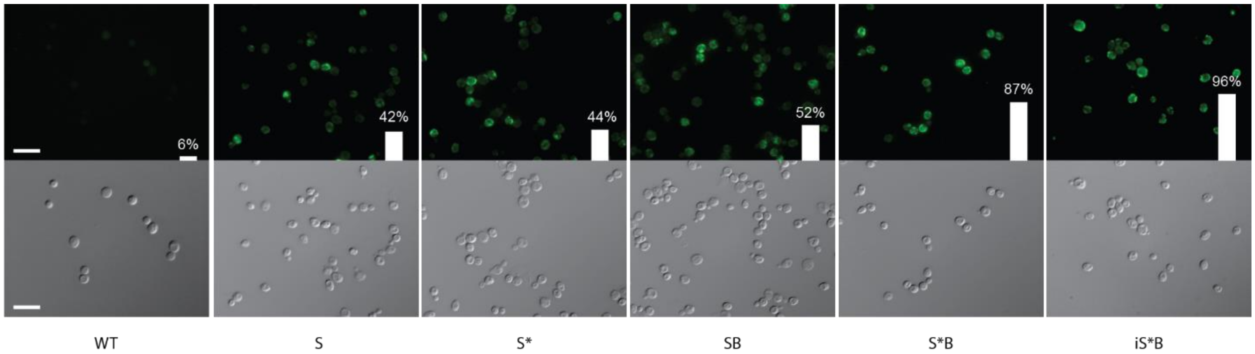

Supplementary Figure 5. Increasing SMF1 expression with increased modifications. Labels from left to right: WT, overexpression of SMF1 (S), SMF1 with K33,34R mutation (S*), S with BSD2 deletion (SB), S* with BSD2 deletion (S*B), and S integrated with BSD2 deletion (iS*B). Bars and values indicate percent expression after subtracting background signal from WT controls. Images and expression scores were calculated using ImageJ. Scale bars represent 10 μm for all images.

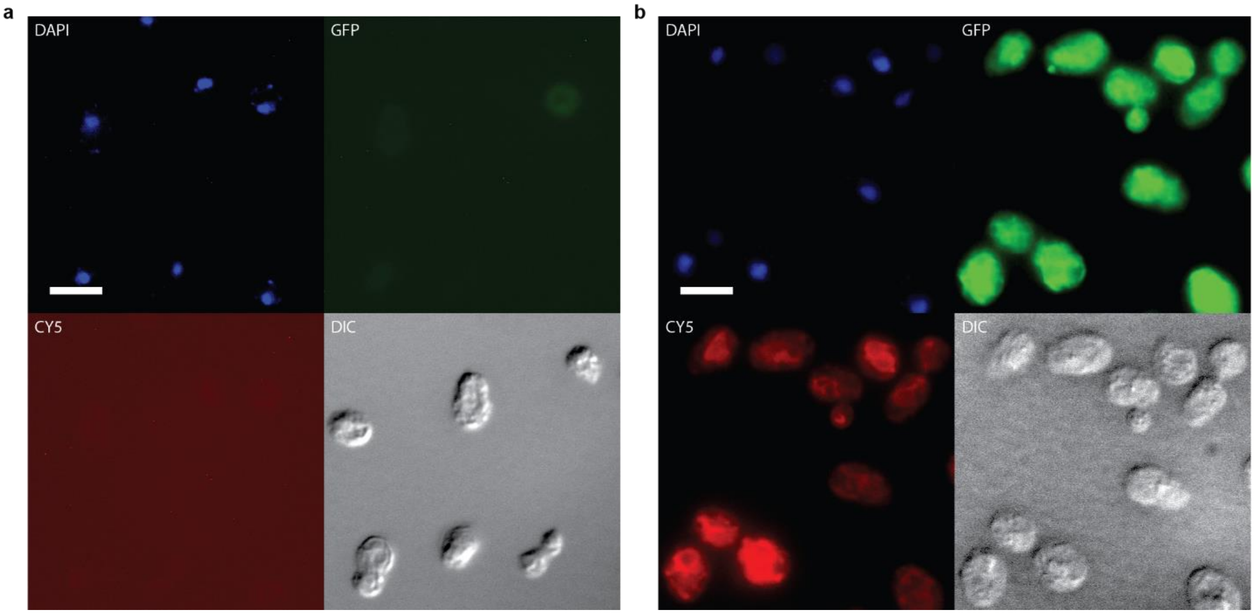

Supplementary Figure 6. Visualization of fluorescently labelled co-expression of SMF1 and CCC1. a) Fluorescent measurements of non-expressing WT strain as a control. **b**) SMF1* was fused with a C' terminus V5 tag, whereas CCC1 was fused with a C' terminus flag tag. Tags were stained with the appropriate primary and secondary antibodies conjugated with either an Alex488 (green; SMF1*) or Alex647(far red; CCC1) dye, respectively. Scale bars represent 5 μm for all images.

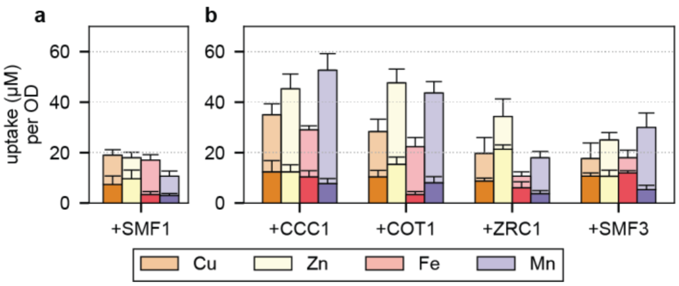

Supplementary Figure 7. Increasing metal uptake with the addition of a vacuole transporter. **a**) Heavier colored bars represent WT metal uptake. Lighter colored overlaid bars represent metal uptake with SMF1 expression (S; no modifications). **b**) Heavier colored bars represent metal uptake with vacuole transporter expression. Lighter colored overlaid bars represent metal uptake with co-expression of SMF1. For all data, the mean ± s.d. of three replicates are shown. Source data are provided as a Source Data file.

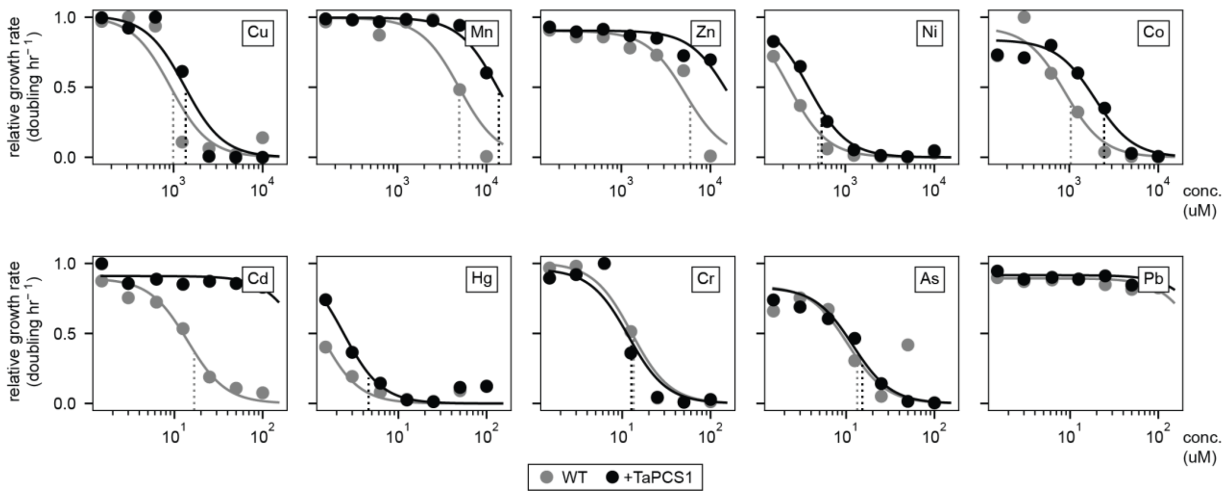

Supplementary Figure 8. TaPCS1 improves metal tolerance for a variety of transition metals. Cu, Mn, Zn, Ni, and Co were added to cultures from 10 μM to 10 mM. Cd, Hg, Cr, As, and Pb were added to cultures from 1 μM to 100 μM. The addition of TaPCS1 slightly enhanced tolerance to Cu, Mn, Zn and Co in the millimolar range. Of the more toxic elements, TaPCS1 specifically conferred tolerance to Cd while changing little against Hg, Cr, and As. Growth rate curves for Pb and Fe were misleading as Fe and Pb precipitated in culture during the 24-hour growth experiment. For all data, the growth rates were fitted with growth curves of three replicates.

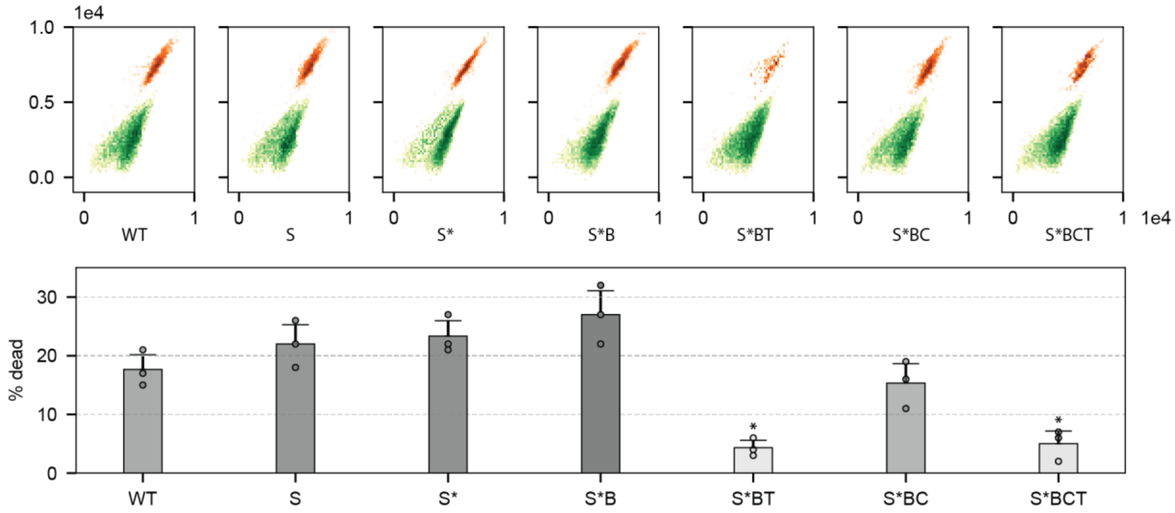

Supplementary Figure 9. Percent survival after metal uptake experiments measured by FACS live-dead assay. Increased transporter expression (**Supplementary Figure 5**) and cadmium uptake (**Figure 2a**) loosely correlated to increase cell death during metal uptake experiments (WT → S → S* → S*B). Expression of TaPCS1 (T) and CCC1 (C) enhanced cell viability despite increased cadmium uptake. Asterisk above bar charts represent significance change in survival percentage ($p < .05$) compared to WT. For all data, the mean ± s.d. of three replicates are shown. Source data are provided as a Source Data file.

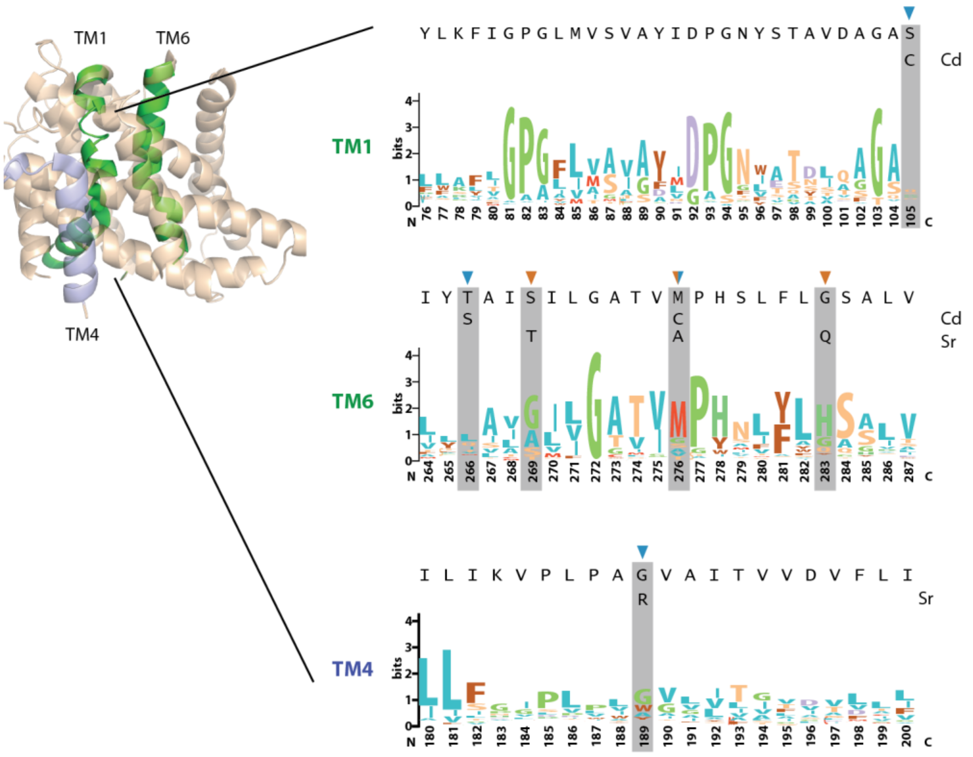

Supplementary Figure 10. Mutations in transmembrane regions TM1 and 6 and TM4. Mutations introduced based on previous findings[34] were M276C for cadmium mutants, and G189R and M276A for strontium mutants. Mutations discovered using the developed transporter assay were S105C and T266S for cadmium mutant mCd, and S269T and G283Q for mSr.

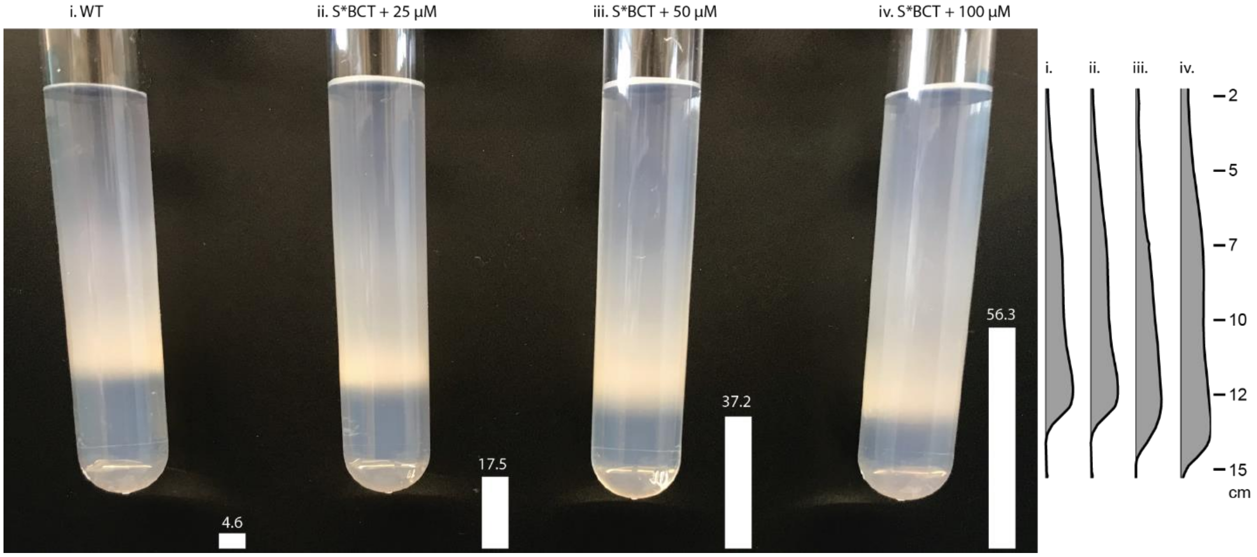

Supplementary Figure 11. Fractionating cells based on metal uptake using rate-zonal density gradient centrifugation. Conditions from left to right were: (i) WT incubated with 100 μM cadmium; (ii), (iii), and (iv) S*BCT strain incubated with 25, 50, and 100 μM cadmium, respectively. Bottom bar charts indicate the amount of metal uptake per condition. Left chart shows the population distribution of migrated cells by measuring the tube's opacity as a function of height using ImageJ. Ticks represent distance measured from the meniscus to the tube's bottom in centimeters.

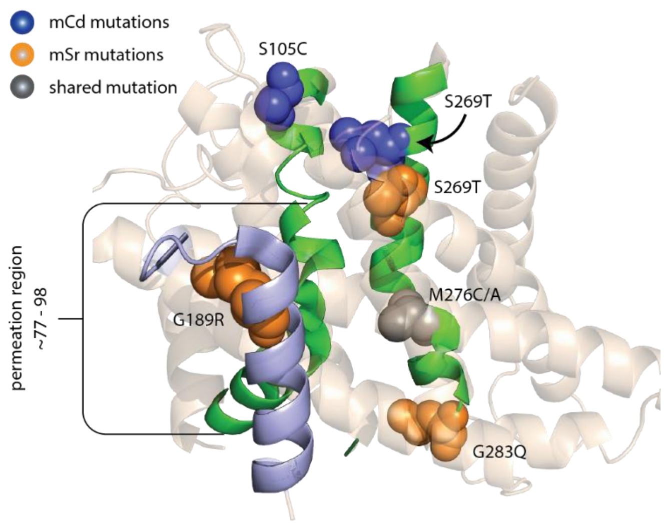

Supplementary Figure 12. Approximate mutation locations for mCd and mSr on homologue DraNramp. PDB accession of DraNramp is PDB 5KTE. Many of the mutations for both mCd and mSr reside on TM6, or at the entry of TM1. This could suggest that the region 77-98 (41-61 for DraNramp) in the first alpha-helix sequence of TM1 is highly sensitive to mutations, as observed in previous works[33,35,36]. This region, referred to as the permeation region[33], has a highly conserved DPGN sequence which may act as an actuator to transport metals through the inner cavity. Whereas, TM6 and 4 may provide the spacing and environment to select for certain metals.

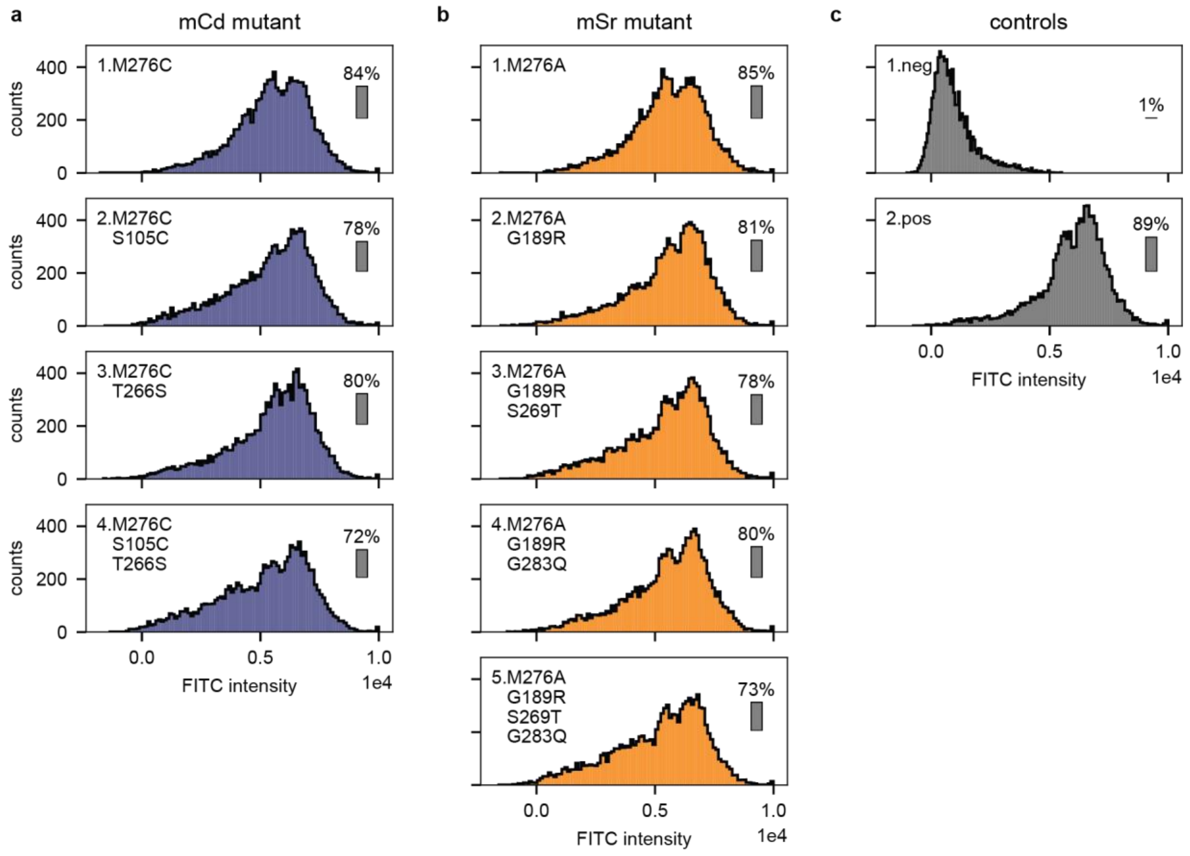

Supplementary Figure 13. Effect of mCd and mSr mutations on SMF1 expression. a) Mutants were fluorescently labelled and measured using flow cytometry. Progressive mutations (1-4; corresponding to numberings in **Figure 5b**) leading to mCd caused little change in transporter expression. **b**) Likewise, a slight reduction in mSr transporter expression was observed, but not significant. **c**) Negative (WT) and positive controls (S*BCT) were also measured to compare expression changes with non-engineered strains.

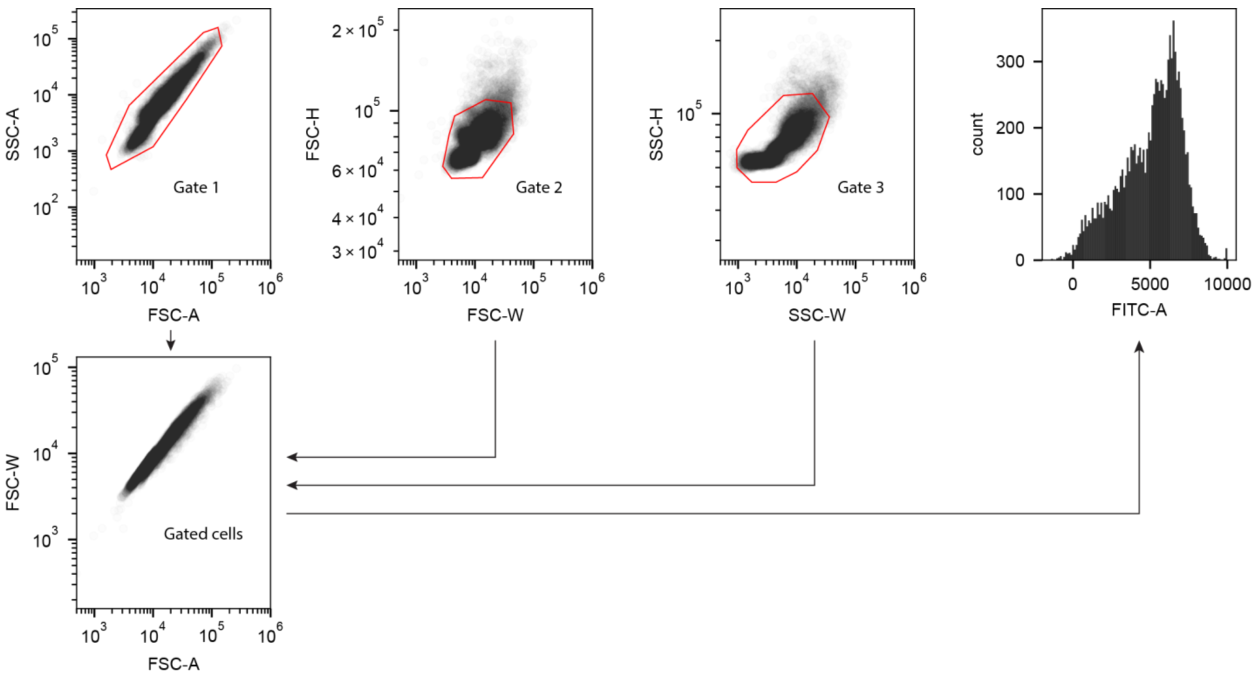

Supplementary Figure 14. Yeast gating strategy for flow cytometry measurements. FSC-A and SSC-A gated cells. FSC-W and FSC-H gated vertically oriented cells (vertical singlets). SSC-W and SSC-H gated horizontally oriented cells (horizontal singlets). Gating on these 3 plots, single cells were measured for fluorescence.

Supplementary Table 1. Hyperaccumulator values for engineered strains.

strain	metal	uptake (µM)	uptake (mg/g)	hyper threshold (mg/g)
ZRT1	Zn	46 ± 6.7	7.3 ± 1.1	10
ZRT2	Zn	35 ± 7.5	3.4 ± 0.7	10
CTR1	Cu	44 ± 5.6	7.5 ± 0.9	1
CTR3	Cu	31 ± 7.0	3.1 ± 0.7	1
FTR1	Fe	17 ± 3.7	2.0 ± 0.4	10
FET4	Fe	23 ± 2.9	2.5 ± 0.3	10
SMF1	Mn	11 ± 2.1	1.3 ± 0.3	10
SMF2	Mn	10 ± 2.6	1.2 ± 0.3	10
Pho84	As	28 ± 3.8	2.8 ± 0.4	0.1
Pho87	As	13 ± 1.0	2.4 ± 0.2	0.1
Pho89	As	18 ± 3.0	2.8 ± 0.5	0.1
Sul1	Cr	25 ± 2.4	2.2 ± 0.2	1
Sul2	Cr	24 ± 1.9	2.7 ± 0.2	1
NRAT1	Al	27 ± 4.3	1.2 ± 0.2	1
S*	Cd	10 ± 1.3	2.2 ± 0.3	0.1
S*B	Cd	21 ± 2.5	4.8 ± 0.6	0.1
S*BC	Cd	53 ± 7.6	9.5 ± 1.4	0.1
S*BCT	Cd	58 ± 5.4	11.4 ± 1.1	0.1
S*BCT	Mn	328 ± 14.8	31.7 ± 1.4	10
mCd	Cd	43 ± 4.6	11.5 ± 1.2	0.1
mSr	Sr	30 ± 3.9	5.1 ± 0.7	N/A

Transporters and strains developed in this work that are within or have exceeded the hyperaccumulating thresholds[12,13] for their respective metal.

Supplementary Table 2. Divalent metal transporter primers for pYES2/CT subcloning.

name	direction	sequence
ZRT1	fwd	TAAGCAGGTACCATGAGCAACGTTACTACG
	rev	TAAGCACTCGAGAGCCCACTTACCGATC
ZTR3	fwd	TAAGCAGGTACCATGGAAAAAATTCCCAGGTG
	rev	TAAGCACTCGAGAGTGAAAAGGGCACTC
CTR1	fwd	TAAGCAGGTACCATGGAAGGTATGAATATGG
	rev	TAAGCACTCGAGGTTATGAGTGAATTTTTCG
CTR3	fwd	TAAGCAGGTACCATGAATATGGGAGGCAG
	rev	TAAGCACTCGAGCAAGCAGCATTTGC
FET4	fwd	TAAGCAGGTACCATGGGTAAAATTGCAGAG
	rev	TAAGCACTCGAGTTTTTCCAACATCATAACC
FTR1	fwd	TAAGCAGGTACCATGCCTAACAAAGT
	rev	TAAGCACTCGAGAAGAGAGTCGGCTTTAAC
SMF1	fwd	TAAGCAGGTACCATGGTGAACGTTGG
	rev	TAAGCACTCGAGACTGATATCACCATGAGAC
SMF2	fwd	TAAGCAGGTACCATGACGTCCCAAGAATAT
	rev	TAAGCACTCGAGGAGGTGTACTTCTTTGC

Cloning strategy involved amplifying the appropriate regions and using the KpnI and XhoI restriction sites.

Supplementary Table 3. Permease primers for pYES2/CT subcloning.

name	direction	sequence
Sul1	fwd	ATAGGGAATATTAAGCTTGGTACCGAGCTCATGTCACGTAAGAGCTC
	rev	AGCGTAGTCTGGAACGTCGTATGGGTAGGATCCACCGCCAACGTCCCATTTAGAAAAATC
Sul2	fwd	ATAGGGAATATTAAGCTTGGTACCGAGCTCATGTCCAGGGAAGGTTA
	rev	AGCGTAGTCTGGAACGTCGTATGGGTAGGATCCACCGCCGATATCCCATTTAGCAAAATC
Pho84	fwd	ATAGGGAATATTAAGCTTGGTACCGAGCTCATGAGTTCCGTCAATAAAGATAC
	rev	AGCGTAGTCTGGAACGTCGTATGGGTAGGATCCACCGCCTGCTTCATGTTGAAGTTGAG
Pho87	fwd	ATAGGGAATATTAAGCTTGGTACCGAGCTCATGAGATTCTCACACTTTCTCA
	rev	AGCGTAGTCTGGAACGTCGTATGGGTAGGATCCACCGCCAGTGCTACCTTTTAAGACG
Pho89	fwd	ATAGGGAATATTAAGCTTGGTACCGAGCTCATGGCTTTACATCAATTTGACT
	rev	AGCGTAGTCTGGAACGTCGTATGGGTAGGATCCACCGCCTGTCATTTGGTATTCCACAC
pYES2/CT	fwd	GAGCTCGGTACCAAGCTTAATATTC
	rev	GGCGGTGGATCCTACCCATACGACGTTCCAGACTACGCTTAAGTTTAAACCCGCTGATCC

Cloning strategy also involved substituting the V5 tag by appending primers with the HA tag.

Supplementary Table 4. Nrat1 primers for pYES2/CT subcloning.

name	direction	sequence
NRAT1	fwd	CTCACTATAGGGAATATTAAGCTTGGTACCATGGAAGGTACTGGTGAAATG
	rev	ACCGAGGAGAGGGTTAGGGATAGGCTTACCCATACTAGCATCTGCCAAATCTT
pYES2/CT	fwd	GGTAAGCCTATCCCTAACCC
	rev	GGTACCAAGCTTAATATTCCCTATAGTG

Nrat1 was first codon-optimized from *O. sativa* and synthesized from Genscript before amplifying and assembling into pYES2/CT.

Supplementary Table 5. Vacuole transporter primers for modified pYES2/CT subcloning.

name	direction	sequence
CCC1	fwd	GTCTTAGAGCTCGTCTTAGAGCTCATGTCCATTGTAGCACTAAAGA
	rev	GTCTTAGGATCCTTAACCCAGTAACTTAACAAAGAAC
COT1	fwd	ATAGGGAATATTAAGCTTGGTACCGAGCTCATGAAACTCGGAAGCAA
	rev	ATCCTTGTAATCACTTCCACCTCCGGATCCATGATCCTCTAAGCAATCAG
ZRC1	fwd	ATAGGGAATATTAAGCTTGGTACCGAGCTCATGATCACCGGTAAAGAATTG
	rev	ATCCTTGTAATCACTTCCACCTCCGGATCCCAGGCAATTGGAAGTATTGC
SMF3	fwd	ATAGGGAATATTAAGCTTGGTACCGAGCTCATGCGATCTTATATGCAGATTC
	rev	ATCCTTGTAATCACTTCCACCTCCGGATCCAAAATGGATGTCGGCAC

The pYES2/CT vector was modified to contain the LEU marker instead of its URA marker. CCC1 was first cloned via SacI and BamHI restriction enzymes, and later modified to contain a downstream flag tag instead of the V5 tag. COT1, ZRC1, and SMF3 were amplified with PCR and Gibson assembled into the modified pYES2/CT vector replacing CCC1.

Supplementary Table 6. Primers used to construct S*BCT strain.

name	direction	sequence
SMF1*	fwd	TCAATTACCACTGTAGAATCTCTCCTATCCCTCAGTTCGAATACTTCTTC
	rev	GAAGAAGTATTCGAACTGAGGGATAGGAGAGATTCTACAGTGGTAATTGA
ΔBSD2::HIS	fwd	TGAGAATAACAAGAACACGTAGTCTAGGAAACTAAGCGCTTATTACTCTTGGCCTCCT
	rev	AAAGTTATATATCTCTTTTTATCATAATGAAGAAGATGGCCCTGATGCGGTATTTTCT
TaPCS1	fwd	CTAAGGGGATGGAGGCTCTT
	rev	ATGGAGGTGGCGTCG
pD1235	fwd	CACCCGCCGGTACAGCGACGCCACCTCCATTTTATCCGTCGAAACTAAG
	rev	GAGGCATGTCAAGAGCCTCCATCCCCTTAGCAGGTTAAATCATGTAATTAGTTATG
iTaPCS1	fwd	CACCCGCACGGCAGAGACCAATCAGTAAAAATCAACGGTTTCATTATCAATACTCGCCAT
	rev	CAAGTGCACAAACAATACTT
part1-GAL	fwd	CAGTCACGACGTTGTAAAACGACGGCCAGTAGTACGGATTAGAAGCCG
	rev	GGTTTTTTCTCCTTGACGT
part2-CCC1	fwd	AGTTTCGACGGATTCTAGAACTAGTGGATCCTCATGTCCATTGTAGCACTA
	rev	GCAGCTTGCAAATTAAAGC
part3-LEU	fwd	GACGCTCGAAGGCTTTAATTTGCAAGCTGCAACTGTGGGAATACTCAGG
	rev	CACGTTGAGCCATTAGTATC
part4-pUC18	fwd	TACCTCTATACTTTAACGTCAAGGAGAAAAAACCGTCATAGCTGTTTCCTGTGT
	rev	ACTGGCCGTCGTTTTA
iCCC1	fwd	AACTGTGGGAATACTCAGGTATCGTAAGATGCAAGAGTTCAGTACGGATTAGAAGCCG
	rev	CACGTTGAGCCATTAGTATC

SMF1* was created using mutagenesis primers altering the K33,34 region (*AAGAAA*) into arginines (*AGGAGA*). ΔBSD2 was created by replacing the BSD2 gene with the HIS3 cassette amplified from pRS303. TaPCS1 was constitutively integrated into the genome by replacing the TRP auxotrophic marker of W303α. The GAL1 inducible CCC1 construct was assembled using Gibson assembly, and product was genomically integrated into the genome.

Supplementary Table 7. Library generation and site-directed mutagenesis primers for SMF1*.

name	direction	sequence
mTM1	fwd	GCAGGTAATGAGAGATATTTTTGCTAAA
	rev	CAAAATGATACAAAGTAGGGAAAATTGATT
mTM6	fwd	CCAAATGTTTGACCACAATGGT
	rev	GTCATAATCTAAAAGCCTTGGCTG
Cd-S105C	fwd	GTCGATGCAGGTGCCTGTAATCAATTTTCCCTAC
Cd-T266S	fwd	CCAAATGTTTGACCACAATGGTATTTATTCTGCTATTTCCATCTTAGGTGC
Cd-M276C	fwd	CTATTTCCATCTTAGGTGCTACTGTTTGTCCACATTCGTTGTTTTTGGGTTC C
Sr-G189R	fwd	GTGCCCCTTCCAGCGAGAGTGGCCATTACTGTT
Sr-S269T	fwd	GACCACAATGGTATTTATACCGCTATTACTATCTTAGGTGCTACTGTTA
Sr-M276A	fwd	CTATTTCCATCTTAGGTGCTACTGTTGCTCCACATTCGTTGTTTTTGGGTTC C
Sr-G283Q	fwd	GTTATGCCACATTCGTTGTTTTTGCAATCCGCTTTAGTGCAGCCAA

Primers used to error-prone PCR regions TM1 and 6 of SMF1 to construct libraries using Agilent's GeneMorph(II) protocol. Afterwards, site-directed mutagenesis primers were used to selectively mutate residues identified to have effects on SMF1 metal preference and uptake.
