# Supplementary material for: Designing yeast as plant-like hyperaccumulators for heavy metals
Source: Nat Commun. 2019 Nov 8;10:5080. doi: 10.1038/s41467-019-13093-6 (PMC6841955; doi:10.1038/s41467-019-13093-6)
Supplement: Supplementary file 3 — Reporting summary [file 41467_2019_13093_MOESM3_ESM.pdf]

nature research

Corresponding author(s): Angela M. Belcher

Last updated by author(s): 9/27/2019

Reporting Summary

Nature Research wishes to improve the reproducibility of the work that we publish. This form provides structure for consistency and transparency in reporting. For further information on Nature Research policies, see Authors & Referees and the Editorial Policy Checklist .

Statistics

For all statistical analyses, confirm that the following items are present in the figure legend, table legend, main text, or Methods section.

n/a	Confirmed	
☐	☒	The exact sample size (*n*) for each experimental group/condition, given as a discrete number and unit of measurement
☐	☒	A statement on whether measurements were taken from distinct samples or whether the same sample was measured repeatedly
☐	☒	The statistical test(s) used AND whether they are one- or two-sided<br>*Only common tests should be described solely by name; describe more complex techniques in the Methods section.*
☐	☒	A description of all covariates tested
☐	☒	A description of any assumptions or corrections, such as tests of normality and adjustment for multiple comparisons
☐	☒	A full description of the statistical parameters including central tendency (e.g. means) or other basic estimates (e.g. regression coefficient) AND variation (e.g. standard deviation) or associated estimates of uncertainty (e.g. confidence intervals)
☐	☒	For null hypothesis testing, the test statistic (e.g. *F*, *t*, *r*) with confidence intervals, effect sizes, degrees of freedom and *P* value noted<br>*Give P values as exact values whenever suitable.*
☒	☐	For Bayesian analysis, information on the choice of priors and Markov chain Monte Carlo settings
☒	☐	For hierarchical and complex designs, identification of the appropriate level for tests and full reporting of outcomes
☒	☐	Estimates of effect sizes (e.g. Cohen's *d*, Pearson's *r*), indicating how they were calculated

Our web collection on statistics for biologists contains articles on many of the points above.

Software and code

Policy information about availability of computer code

Data collection	Outputs from instruments or machines (e.g. ICP, plate readers) were exported or copied into a text readable format (e.g. .csv).
Data analysis	Excel and python were used to compile and analyze data. Open-source packages such as numpy, pandas, and matplotlib were used to quantify and graph results. Multi-sequence alignments and clusterings were performed using online services such as PFam, ClustalOmega, TCoffee, and Espript3.

For manuscripts utilizing custom algorithms or software that are central to the research but not yet described in published literature, software must be made available to editors/reviewers. We strongly encourage code deposition in a community repository (e.g. GitHub). See the Nature Research guidelines for submitting code & software for further information.

Data

Policy information about availability of data

All manuscripts must include a data availability statement. This statement should provide the following information, where applicable:

- Accession codes, unique identifiers, or web links for publicly available datasets
- A list of figures that have associated raw data
- A description of any restrictions on data availability

The datasets generated during and/or analyzed during the current study are available from the corresponding author on request.

Field-specific reporting

Please select the one below that is the best fit for your research. If you are not sure, read the appropriate sections before making your selection.

☒ Life sciences ☐ Behavioural & social sciences ☐ Ecological, evolutionary & environmental sciences

Life sciences study design

All studies must disclose on these points even when the disclosure is negative.

Sample size	Sample sizes of 3-5 were used in all experiments.
Data exclusions	No data or outliers were excluded in the results.
Replication	Metal uptake experiments were performed on samples prepared on separate days as well as measured on separate days when possible. Library generation and density gradient centrifugation screening, an experimental pipeline introduced in this manuscript, may not necessarily provide similar results per experiment or experimeter due to the nature of library screening.
Randomization	Samples were not randomized during experimentation.
Blinding	Investigators were not blinded during the study.

Reporting for specific materials, systems and methods

We require information from authors about some types of materials, experimental systems and methods used in many studies. Here, indicate whether each material, system or method listed is relevant to your study. If you are not sure if a list item applies to your research, read the appropriate section before selecting a response.

Materials & experimental systems

n/a	Involved in the study	
☐	☒	Antibodies
☒	☐	Eukaryotic cell lines
☒	☐	Palaeontology
☒	☐	Animals and other organisms
☒	☐	Human research participants
☒	☐	Clinical data

Methods

n/a	Involved in the study	
☒	☐	ChIP-seq
☐	☒	Flow cytometry
☒	☐	MRI-based neuroimaging

Antibodies

Antibodies used

V5 Epitope Tag Antibody (2F11F7), HA Epitope Tag Antibody (5B1D10), Flag Epitope Tag (FG4R), Flag Epitope Tag (Rabbit/igG), Goat anti-Mouse IgG (H+L) Cross-Adsorbed Secondary Antibody Alexa Fluor 488, Goat anti-Rabbit IgG (H+L) Highly Cross-Adsorbed Secondary Antibody Alexa Fluor 647.

Validation

All antibodies were commercially purchased and validated.

V5 primary antibody: https://www.thermofisher.com/order/genome-database/antibody/V5-Epitope-Tag-Antibody-clone-2F11F7-Monoclonal/37-7500

HA primary antibody: https://www.lifetechnologies.com/order/genome-database/antibody/HA-Epitope-Tag-Antibody-5B1D10-Monoclonal/32-6700

Flag primary antibody (mouse): https://www.thermofisher.com/order/genome-database/antibody/FLAG-Epitope-Tag-DYKDDDDK-Antibody-clone-FG4R-Monoclonal/MA1-91878

Flag primary antibody (rabbit): https://www.thermofisher.com/antibody/product/FLAG-Epitope-Tag-DYKDDDDK-Antibody-Polyclonal/PA1-984B

Goat anti-Mouse secondary antibody Alexa Fluor 488: https://www.thermofisher.com/antibody/product/Mouse-IgG-H-L-Secondary-Antibody-Polyclonal/A-11001

Goat anti-Rabbit secondary antibod Alexa Fluor 647y: https://www.thermofisher.com/antibody/product/Goat-anti-Rabbit-IgG-H-L-Secondary-Antibody-Polyclonal/A-21245

Flow Cytometry

Plots

Confirm that:

- [x] The axis labels state the marker and fluorochrome used (e.g. CD4-FITC).
- [x] The axis scales are clearly visible. Include numbers along axes only for bottom left plot of group (a 'group' is an analysis of identical markers).
- [x] All plots are contour plots with outliers or pseudocolor plots.
- [x] A numerical value for number of cells or percentage (with statistics) is provided.

Methodology

Sample preparation	W303 yeast strains were grown on plates, transferred to liquid culture, and processed according to the methods section for experiments and analysis.
Instrument	BD FACS LSR II, BD FACS Celesta
Software	Python, with library FlowCytometryTools (https://eyurtsev.github.io/FlowCytometryTools/index.html)
Cell population abundance	Yeast strains were measured at 0.1 OD600, or roughly 1 million cells per mL.
Gating strategy	FSC-A and SSC-A was used to gate on cells. FSC-W and FSC-H was used to gate vertically orientated single cells (vertical singlets). SSC-W and SSC-H was used to gate horizontally orientated single cells (horizontal singlets). After gating on these 3 plots, single cells were measured for fluorescence.

- [x] Tick this box to confirm that a figure exemplifying the gating strategy is provided in the Supplementary Information.
