# Supplementary material for: Designing yeast as plant-like hyperaccumulators for heavy metals
Source: Nat Commun. 2019 Nov 8;10:5080. doi: 10.1038/s41467-019-13093-6 (PMC6841955; doi:10.1038/s41467-019-13093-6)
Supplement: Supplementary file 2 — Peer Review [file 41467_2019_13093_MOESM2_ESM.pdf]

Reviewers' comments:

Reviewer #1 (Remarks to the Author):

It is an appealing concept to exploit the unique power of yeast genetics for tackling global metal contamination problems. The authors set out to engineer yeast cells so that they would acquire characteristics of metal hyperaccumulating plants. Some of the concerns I have about the manuscript can maybe illustrated best by directly referring to hyperaccumulating plants:
1. Metal hyperaccumulating plants show true uptake and translocate metals to aboveground tissues. The experiments reported here fall short of demonstrating true accumulation. Reading the methods description I found no mention of a washing procedure for cells (e.g. with 10 mM $CaCl_2$ or with EDTA). Thus, it is not really clear how much of the reported metal content (in my view not appropriate to express it as μM per unit OD, i.e. a concentration per OD) is actually intracellular as opposed to bound to the cell wall.
2. Hyperaccumulation is defined as metal accumulation that is two or three orders of magnitude higher than the accumulation shown by "normal plants" grown under the same conditions in the field. The increases shown here are not nearly of the same magnitude (about 10fold higher than WT). Part of the reason is that external metal concentrations were very high, a situation that also in other organisms can lead to considerable accumulation that on should not refer to as hyperaccumulation.
3. The motivation of this study is possible application for metallic waste removal. While I acknowledge that first the engineering has to be achieved I would still expect that at least a rough idea is presented how yeast cells could be used for this purpose. Just to mention one aspect: after a while cell lysis would release metals again; this is entirely different for plants where the accumulating organs can be harvested.
Another major issue: several of the reported results are not novel. This applies to some of the metal uptake effects, to tolerance gain when expressing TaPCS (which is from wheat, not rice!), and also to the Nramp transporter with altered metal specificity.

Reviewer #2 (Remarks to the Author):

In this work, a yeast metal hyper accumulator has been engineered based on knowledge from plant hyper accumulators. The yeast hyperaccumulator strain is assembled based on the knowledge of the basis of metal hyperaccumulation in plants. This includes expression of plasma membrane metal transporters, expression of vacuolar metal transporters and expression of metal chelators in the yeast cytosol. In addition, the metal selectivity of the plasma membrane transporter Smf1p is altered from manganese to both cadmium and strontium by site mutagenesis and site semi random imperfect PCR mediated site mutagenesis. Selection of Smf1p proteins with altered specificity are determined by iterative mass based density gradient selection and competition assay rounds. The results are two yeast strains that convincingly hyperaccumulate cadmium and strontium in a plant hyperaccumulator-like manner. The paper is interesting and present results that are very relevant to the fields of both bio-remediation and metal transport.

Unfortunately, there are several issues that need to be addressed before publication of the manuscript can be recommended. A number of the experimental approaches are very sparingly described, several figures are not correctly explained or numbered, a number of text sections are hard to understand, and some basal approaches to data calculations are not viable.

A non-exhaustive list of examples is:

- Figure 1 B: Figure text is minimal, and the Materials and Methods section is unclear. The Y-axis unit of μM/OD is hard to access. Materials and Methods state that cultures are grown at 100 μM concentrations of all metals, however the text later describes the severe inhibition of WT yeast growth

in cadmium concentrations above 10 µM; the presentation of the data is not very transparent.

- Figure 2a) A description of Figure 2a is minimal, it is not stated that the upper part is flow cytometry, or that the lower part is ICP-AES measurements.

- Figure 3 d: Titration curve of manganese and cadmium competition: The subfigure is mislabeled in the figure legend as Figure 3c. Units are missing from the Y-axis, an explanation of the process is missing from the figure text as well as no description is evident in the Materials and Methods section of how this was performed.

- Figures 2c, 3a+b, 5d: several text segments refers to the significant difference or lack of significant difference between samples, however no indicators of statistical significance are evident in the paper.

- Line 141-143 and Supplementary Figure 2/Figure 7: In a large part of the work, measurements of metal uptake/optical density is converted into a measure of mg metal /grams of dry weight and this is used among other things to relate to the status of hyperaccumulator. The method used for these measurements is described differently in the figure text and in the Materials and Methods section. It is doubtful whether weight/OD correlations can be transferred between lines overexpressing transporters and, in addition, accumulating heavy metals. In the view of this reviewer this wild type weight/OD correlation seems too weak to use for calculating actual metal concentrations in the transformant yeast lines.

- Supplementary Figure 1/ Figure 6: the figure shows microscope images of yeast cells labeled with fluorescent antibodies to confirm expression of inserted transporters, however, the seemingly cytosolic localization of the tagged molecules underlines the need for a negative control in order to demonstrate that what is tagged is actually the membrane proteins in question.

- Accession numbers: Throughout the paper, accession numbers for the genes implemented are missing. In addition, species denominators are largely missing and in one case where present is wrong (Line 206 states that TaPCS1 originates from rice/Oryza sativa)

Reviewer #3 (Remarks to the Author):

Contamination of soils and water by toxic metals is widespread and of growing concern. Various bioremediation approaches may be used to clean up polluted soils but the progress of developing plants that hyper-accumulate metals has been slow. In this study, the authors used baker's yeast to create strains that hyper-accumulate metals. The strategy was based on: 1) increased expression of metal importers; 2) increased expression of proteins that transport metals into vacuoles; 3) expression of cytosolic metal-binding proteins. The authors then developed a screen to identify metal hyper-accumulator strains expressing forms of SMF1 with altered substrate specificity.

Overall, this is an interesting study that systematically examines the combined effect of gene overexpression (points 1-3 above) on metal hyper-accumulation (and cell survival). The novelty of this part of the manuscript is limited to the simultaneous co-expression of different genes already known individually (or in some cases combined 2 by 2) to contribute to metal uptake. The most novel aspects of the study includes 1) the density-based screening approach to identify metal hyper-accumulators coupled to colorimetric assays for metal specificity and 2) the generation of SMF1 variants that appear

to have altered substrate specificity. However, the claim that these SMF1 variants have altered substrate specificity is not supported by solid biochemical data (e.g. quantification of protein amounts in the plasma membrane and/or reconstitution experiments). Efforts should be made to cement this claim as engineering transporters for altered substrate specificity is a key aim of this work. Thus, the advance from previous studies appears moderate when it comes to novel mechanistic understanding and overall impact.

Specific comments
1. line 131: it is claimed that Nrat1 promotes selective uptake of aluminium. This claim is not supported by data (only Al has been tested as substrate).
2. line 136: the statement 'preferential accumulation of aluminium' is not supported by experimental data.
3. Please add p-values for significance for the metal accumulation assays (e.g. Fig 1b, 2b, 5b etc) and adjust the text accordingly.
4. line 199: GSH functions as a metal chelator in yeast on its own (and not only as a constituent of phytochelatin) – please mention this fact.
5. line 206: TaPCS1 – Ta stands for Triticum aestivum (wheat) and not rice. Please correct.
6. line 2249-251: the sentence 'Multi-aligning the…. revealed region…' is unclear. Please clarify what was revealed (e.g. highest homology?).
7. line 298: Please explain the experimental set-up: was it iterative rounds of growth to saturation?
8. line 300: un-engineered S* - S* is a mutated form of SFM1. Please correct.
9. Fig 2: It is unclear what the top panel in Fig 2a shows. Fig 2c lacks label of the y-axis.
10. Fig 4a: Please explain what black and red letters denote.
11. Fig 5b: Please indicate the strain background in the legend.
12: Fig S1 (labelled Fig 6 in the submitted MS): The fluorescent images do not show clear/uniform plasma membrane localization of the transporters. Instead, the fluorescent signal is often visible as dotted structures inside the cell. Thus, using total fluorescence as a measure of functional protein expression is clearly problematic.

Reviewer #4 (Remarks to the Author):

The Sun et al. paper is an original study aiming to engineer budding yeast, the laboratory model widely used to characterize plant metal transporters, toward an hyperaccumulator microorganism that could be used for bioremediation purposes.
The main functional adaptations of known plant hyperaccumulators were combined and successfully applied to the yeast model (improved expression of metal importer at the cell surface combined to overexpression of vacuole metal importer and synthesis of intracellular organic metal chelator that can be stored in the vacuole) to demonstrate increased capacity for Cd accumulation in yeasts that remained viable.
An approach to select for enhanced accumulation of toxic heavy metals (Cd, Sr) based on semi-targeted mutagenesis of the yeast metal importer Smf1 is presented. It uses rate-zonal density centrifugation to enrich heavier cells with stronger metal load. The resulting metal uptake properties of two Smf1 variants are described, which catalyze either preferential accumulation of Cd vs Mn, i.e., metal preference opposite to Smf1 native affinity, or reduced Mn uptake but neoaccumulation of Sr. In both cases novel properties resulted from targeted mutations and were accentuated by subsequent random mutagenesis.
Hence a proof of principle is suggested that yeast cells can be engineered to accumulate toxic metals at levels similar to those reported for plant hyperaccumulators.
After significant revision this study should foster interest in developing yeast hyperaccumulator strains needed for bioremediation approaches in industrial settings such as the textile industry.

Methodological criticisms:

Quantitation of metal hyperaccumulation: Growing yeast that were pre-induced to express genes of interest, were exposed to metals for 4h then cell pellets were processed for ICP-AES analyses. Then the relationship established between OD of WT yeast cultures and dry weight of the lyophilized cells was used to calculate metal accumulation (mg per kg of dry weight) for the mutant studied.
This approach raises several questions: For instance, do engineering processes alter the relationship between yeast OD and dry weight? Also, yeast cell wall constitutes a significant portion of dry weight and it is unclear whether external metal adsorption on yeast has been distinguished from metal internalization. Answering such questions would strengthen the claim that engineered yeasts behave as hyperaccumulators.

Fluorescent visualization of over-expressed transporters (Suppl. Figs 1&4) does not allow one to discriminate the site of protein expression (plasma membrane or vacuole).

l. 100: metal exporters, which could release metals out of the vacuole or out of yeast cells are only briefly mentioned (l. 225: antiporters?, and in the discussion). They are not presented in Fig. 1a. The activity of Smf3 in the assays presented (Fig. 2b and Suppl. Fig. 5) should be clarified relatively to the role of vacuole importers; in the case of iron, this could require co-examining the role of Fre6p as well.

Titration of uptake: presentation of the data renders interpretation difficult (Fig. 3b, Fig. 5c); how could possibly maximal uptake occur when adding metal at the lowest dose? Figure legend should mention that x axis represents test metal concentration (Mn or Cd, Cd being used alone or in the presence of 100 uM Mn).

Isolation of metal hyperaccumulating mutants by rate-zonal density centrifugation: yeast density refers to the weight of an individual cell per unit volume; rate zonal approach was deemed more appropriate by the authors than isopycnic centrifugation "as previous studies have shown that yeast maintain a relatively constant density despite external influences" (l. 269-270), so yeast mutants were separated according to their rate of sedimentation. That rationale is unclear to me, given that to maintain density a hyperaccumulating mutant would be expected to vary cell volume or shape; what consequence then on the correspondence between OD and dry weight measurements and accuracy of hyperaccumulation data? In that case wouldn't it be preferable to relate dry weight to colony forming ability?

Functional characterization of the hyperaccumulating mutants: due to possible issues relating to OD variations between mutants it would be informative to compare the expression levels of mutant proteins (Fig. 5b).

Only two mutants, each combining 2 neomutations, are described as a result of 4 rounds of selection pipeline. Are these the only mutants that were obtained? This would mean that less than 1 mutation was obtained per round of selection. If so, what can be deduced regarding the efficiency of the screening procedure? Otherwise it would be useful to indicate the overall number of mutants obtained per round of selection and whether mutation map within TM or intervening loops.

Lastly, in several parts of the text the authors' statement is not clear. A few examples:
l. 67-68: what is the strict definition for Ni hyperaccumulator
Do reported values refer to dry weight of whole plant or specific parts that accumulate metals?
l. 108: text refers to Supp. Fig. 1 that is instead labelled Figure 6; labelling should be corrected for all the supplemental data
l. 300-302 vs l. 766-767: sentences in the text and figure legend have opposite meaning, hence difficult to evaluate the data presented

The quality of presentation should be improved for some figures as well:

-Fig.1b is not easy to read, either present values from which WT levels were subtracted or a presentation like that used in Suppl. Fig. 5.
-Fig. 3c and 3d: legends do not correspond.
-Fig. 5b and 5c: M276 or M267?

Discussion:
Agreed yeast expressing neomutated forms of Smf1p preferentially accumulate Sr or Cd over Mn; yet in absence of sufficient biochemical data or structural explanation care should be taken not to oversell the mutagenesis approach by qualifying the transporter metal selectivity.

For instance, l. 298-308, mCd preference for Cd is relative (Fig. 5d lower left) since it appears more clearly in the 2 first rounds of uptake, implying rather low affinity for Cd; so that it is a decrease in Mn affinity and increased permissiveness for Cd uptake that best describe mCd mutant.

There is no discussion of the possible impact of the mutations obtained on Smf1* structure ; 3D homology modelling should point the location of the mutated residues to show whether they colocalize and/or contribute to delineate some inner cavity used for metal translocation.

Mentioning the use of other compartments for metal accumulation such as Golgi, RE and mitochondria does not seem realistic: metal export from these compartments is key to maintaining their functional integrity (e.g., plant chloroplasts).

More generally the discussion should emphasize that eventually engineered hyperaccumulator yeasts should not be able to survive outside a defined industrial environment.

Thank you for giving us the opportunity to re-submit our manuscript, as we believe the reviewer comments (addressed below) have made our work stronger and clearer to understand. Many of the criticisms among all 4 reviewers were predominantly on the clarity and descriptiveness of our work, so we have made substantial edits to the Results and Methods sections. Major points that were raised and corrected in the manuscript were:

- **Explaining our experimental controls to differentiate metal uptake due to engineered transporters versus from non-specific metal binding onto the cell wall. The controls for non-specific metal binding were negligible and did not impact our results.**
- **Adding controls and a better interpretation of the fluorescent stains of expressed transporters in the Supplemental Figures 1, 4, and 5.**
- **Explaining our rationale for correlating yeast culture density (OD) to dry weight as a means to calculate metal uptake per yeast dry mass (Supplemental Figure 3). This was a fundamental step in order to make 1 to 1 comparisons with current literature hyperaccumulator values (which report values by mass). Our method provided the most practical and feasible means to derive such a relationship.**
- **Figures, figure legends, and corresponding methods have been heavily edited to improve clarity, as pointed out by the reviewers.**

Reviewer #1 (Remarks to the Author):

It is an appealing concept to exploit the unique power of yeast genetics for tackling global metal contamination problems. The authors set out to engineer yeast cells so that they would acquire characteristics of metal hyperaccumulating plants. Some of the concerns I have about the manuscript can maybe illustrated best by directly referring to hyperaccumulating plants:

1. Metal hyperaccumulating plants show true uptake and translocate metals to aboveground tissues. The experiments reported here fall short of demonstrating true accumulation. Reading the methods description I found no mention of a washing procedure for cells (e.g. with 10 mM $CaCl_2$ or with EDTA). Thus, it is not really clear how much of the reported metal content (in my view not appropriate to express it as µM per unit OD, i.e. a concentration per OD) is actually intracellular as opposed to bound to the cell wall.

Thank you for this thoughtful question. From our understanding of this comment, we made it unclear whether or not metal was truly translocated *into* the cell, rather than being non-specifically bound to the cell wall. Our method in calculating metal uptake was to measure the metal content of the supernatant to then calculate the amount of metal removed by the cells. In order to take into consideration non-specific metal uptake, a WT sample (non-expressing strain) was processed under similar conditions and measured for metal uptake. These controls are shown at the end of each bar chart in Figure 1 b-d, Figure 2a, Figure 3a,b and Figure 5b. These WT controls show minimal non-specific absorption of metal on or into the cell. For example, the metal uptake of cadmium for WT was 3.7 $\pm$ 1.3 μM/OD, whereas for our engineered strain S*BCT cadmium uptake was 57.7 $\pm$ 5.4 μM/OD, ten times more (Figure 3A).

As well, in each experiment a sample without any cells was measured to test for non-specific metal binding to the sample tube or from loss during the experimental procedure. These values were negligible and did not affect our experiments. We have edited the Results and Methods section (pages 26-28) to add more detail on this experimental procedure, and how we considered non-specific uptake by using WT controls in all experiments.

With respects to the units used, we report both μM/OD as well as mg metal per gram of yeast dry weight (mg/gDW). For μM/OD, we wanted to use a metric that was easily comparable between samples. We wanted to normalize against cell density, thus OD, and wanted to compare levels of uptake of different metals equally, thus using molarity (rather than ppm or weight). We also convert these values and report in mg/gDW to better compare with current literature values which were cited in the text.

2. Hyperaccumulation is defined as metal accumulation that is two or three orders of magnitude higher than the accumulation shown by “normal plants” grown under the same conditions in the field. The increases shown here are not nearly of the same magnitude (about 10fold higher than WT). Part of the reason is that external metal concentrations were very high, a situation that also in other organisms can lead to considerable accumulation that on should not refer to as hyperaccumulation.

This is a good point which we would like to clarify. We are using definitions of hyperaccumulation that have been used separately by three authors in the past, Prasad, Kramer, and Branquinho which determines the level of hyperaccumulation based on metal accumulated per biomass dry weight (mg/kg). The text in which this was defined (page 3) in the manuscript is highlighted in yellow, and is copied here:

Out of all plants, there are more than 400 species that hyperaccumulate heavy metals; the stricter definition being an accumulation of 100 mg/kg (0.01% dry wt.) of cadmium or arsenic, 1,000 mg/kg of (0.1% dry wt.) of cobalt, copper, chromium, aluminum, nickel and lead, and 10,000 mg/kg (1% dry wt.) of manganese and zinc.

Although our data shows 10-fold increases in metal accumulation at 100 μM metal concentrations, our engineered yeast do reach these hyperaccumulating thresholds. We also show in our WT (non-expressing) controls that even at 100 μM metal concentrations non-specific binding contributes minimal metal uptake in comparison to the engineered strains. For example, WT non-expressing strains had metal uptake of Cu, Zn, Fe, and Mn below 8% (Figure 2A) with the addition of 100 μM metal, whereas expressing strains such as ZRT1 and CTR1 had specific uptake of Zn and Cu, respectively above 40%. More so, the addition of a vacuole transporter in the S*BCT strain increases Cd uptake above 50%. Supplemental Table 1 provides a list of strains that have reached these thresholds.

3. The motivation of this study is possible application for metallic waste removal. While I acknowledge that first the engineering has to be achieved I would still expect that at least a rough idea is presented how yeast cells could be used for this purpose. Just to mention one aspect: after a while cell lysis would release metals again; this is entirely different for plants where the accumulating organs can be harvested.

Thank you for the great suggestion. For actual applications yeast will need to be harvested before major cell death occurs. In our engineered strains cell death begins to occur after 12 hours (Figure 3b,c and Supp. Figure 8). Although not fully developed in the lab, we do plan to create cartridges, or yeast-based filtering units where waste water can be controllably flowed through the yeast and incubated at fixed amounts of times to avoid any cell death or leakage of metal back into the media. This topic has been more thoroughly discussed in the Discussion section (page 17-18) with references appropriately added. The statement is copied below:

Actual application of these yeast strains in real-world settings would require another layer of technological development, such as a container or cartridge to secure yeast in a controllable unit. Fortunately, these technologies exist, such as yeast packaging, freeze-drying, and delivery which are routine technologies found in the consumer market. A potential concept is to grow and store yeast in commercial filter-like cartridges where they can be housed in filtering units with size-exclusion cutoffs to prevent yeast leakage back into the purified waters. An additional layer of safety is to genetically modify these yeast with kill switches, or a metabolic reliance on a controlled nutrient such that removal from these containers will result in cell death[45].

Another major issue: several of the reported results are not novel. This applies to some of the metal uptake effects, to tolerance gain when expressing TaPCS (which is from wheat, not rice!), and also to the Nramp transporter with altered metal specificity.

We are extremely embarrassed to have mislabeled TaPCS1 as a rice protein, when it is clearly from wheat! We may have mistakenly wrote rice because another protein we use, Nrat1, comes from *Oryza sativa*, and we may have incorrectly switched labels.

We have changed the wording to clarify that using TaPCS1 for metal tolerance is not a novel development in this manuscript. Also, attempts at modifying Nramp transporters have been done in the past, most notably in papers cited in this manuscript by Bozzi and Ehrnstorfer et. al. Instead, what we wanted to show is the synthesis of different concepts from plant and microbiology to create yeast strains that can hyperaccumulate toxic metals in a controllable manner. Therefore, we use the TaPCS1 machinery in an additive fashion to augment the capabilities of the engineered membrane (SMF1) and vacuole transporters (CCC1) to better uptake and retain heavy metals such as cadmium. Figure 3a-c show increasing metal uptake reduces cell viability; however, with the expression of TaPCS1 cell viability is rescued. More so, TaPCS1 reduces the amount of metal leaked back out, potentially also due to cell death, even after 12 hours of uptake.

Our statement on this issue is copied below and on page 11 in the results section:

The purpose of this work was to demonstrate that TaPCS1, in conjunction with membrane and vacuole transporters, can enhance metal uptake and retention due to enhanced metal tolerance. The subsequent results which combine SMF1, CCC1, and TaPCS1 show that these modules can act additivity to incrementally improve metal hyperaccumulation.

As for mutagenizing SMF1, we wanted to show a novel screening strategy using rate-zonal density gradient centrifugation. Altering SMF1's metal specificity may have been done traditionally through alanine screening or point mutations in the past, but we demonstrate the creation of unique mutants through density changes due to the amount of metal uptaken. This pipeline is illustrated in Figure 4. We believe that altering SMF1 specificity for cadmium and strontium for the application of heavy metal uptake is a novel pursuit for bioremediation purposes, and this methodology can be tailored for other transporters and metals.

<u>Reviewer #2 (Remarks to the Author):</u>

In this work, a yeast metal hyper accumulator has been engineered based on knowledge from plant hyper accumulators. The yeast hyperaccumulator strain is assembled based on the knowledge of the basis of metal hyperaccumulation in plants. This includes expression of plasma membrane metal transporters, expression of vacuolar metal transporters and expression of metal chelators in the yeast cytosol. In addition, the metal selectivity of the plasma membrane transporter Smf1p is altered from manganese to both cadmium and strontium by site mutagenesis and site semi random imperfect PCR mediated site mutagenesis. Selection of Smf1p proteins with altered specificity are determined by iterative mass based density gradient selection and competition assay rounds. The results are two yeast strains that convincingly hyperaccumulate cadmium and strontium in a plant hyperaccumulator-like manner. The paper is interesting and present results that are very relevant to the fields of both bio-remediation and metal transport.

Unfortunately, there are several issues that need to be addressed before publication of the manuscript can be recommended. A number of the experimental approaches are very sparingly described, several figures are not correctly explained or numbered, a number of text sections are hard to understand, and some basal approaches to data calculations are not viable.

Thank you for pointing out the lack of clarity in our manuscript. We both want to apologize for these mistakes and also thank you for meticulously bringing them to our attention. We agree, in that we can do better to provide more detail and guidance to the reader when describing our experimental protocol, how the data is presented in the figures, and any additional textual or visual annotations to make interpretation clearer.

A non-exhaustive list of examples is:

- Figure 1 B: Figure text is minimal, and the Materials and Methods section is unclear. The Y-axis unit of µM/OD is hard to access. Materials and Methods state that cultures are grown at 100 µM concentrations of all metals, however the text later describes the severe inhibition of WT yeast growth in cadmium concentrations above 10 µM; the presentation of the data is not very transparent.

Items that have changed in the manuscript:

In the Methods section, we wrote a more detailed description of how metal uptake values and units of μM per OD were derived (page 26-28). A description of the x and y axis of the bar charts has been added to Figure 1's legend, copied below.

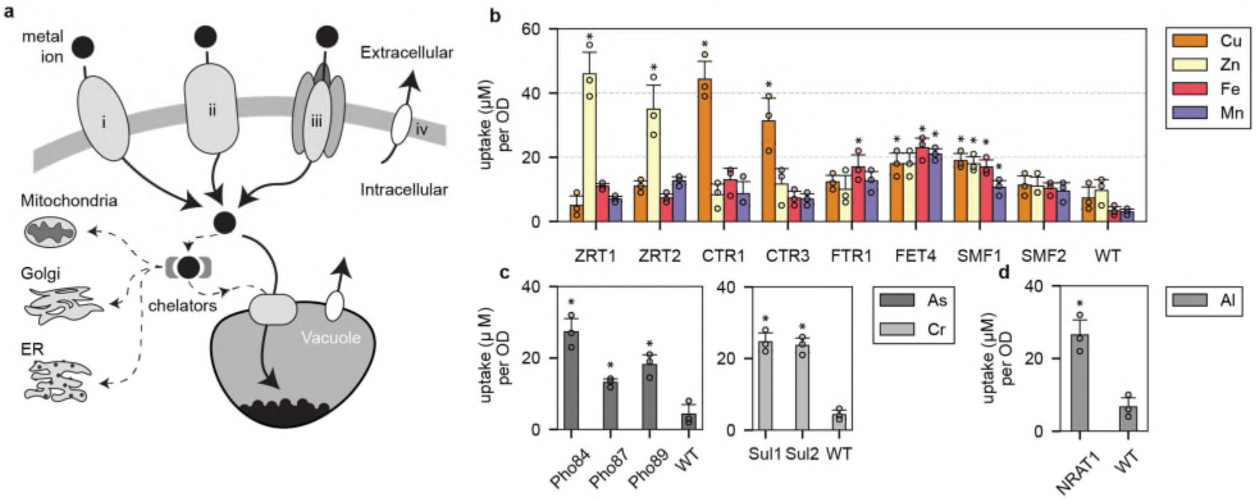

Figure 1 | A variety of metal transporters can be used to selectively internalize heavy metals into yeast. a) A simplified schematic of metal transport in a eukaryotic cell. Membrane transporters can be divalent metal transporters (i), permeases (ii), metal transporters that are modified or found to have auxillary metal transport function (iii), or antiporters which are used to remove excess metals out of the cell (iv). **b**) Bar coloring indicates metal measured, with over-expressed transporter labeled on the x-axis. Values are reported in μM of metal uptake normalized per yeast culture density (μM/OD). Yeast metal transporters for zinc (ZRTs), copper (CTRs), iron (FTRs and FETs), and manganese (SMFs) were overexpressed and studied for metal hyperaccumulation. A WT strain was also tested in parallel for each metal to measure non-specific metal uptake. **c**) The same study was performed for phosphate and sulfate permeases (PHOs, and SULs) which showed transport of arsenate and chromate, respectively. **d**) The Nrat1 transporter, previously shown to uptake trivalent metals in certain strains of rice, was expressed and showed aluminum(III) transport. Asterisk above bar charts represent significance increase in uptake compared to WT ($p < .05$) for strains mentioned in the text. For all data, the mean ± s.d. of three replicates are shown.

Also in the Methods is an explanation for using the units μM per OD (page 27). We wanted to normalize for cell count, hence having OD as a denominator, and compare the level of metal uptake between different metals, hence using molarity (μM). Alternatively, we cited papers presenting data in units of mass, such as gram of metal uptake per gram of cell dry weight (mg/gDW). We also convert to these units; however, we believe using molarity normalized against the quantity of cells will help the reader better compare the level of uptake between strains and the various metals used in this manuscript.

Yes, all metal uptake experiments were performed at 100 μM of metal at 1 OD of cells. This has been more clearly described in the Methods (page 29). Cells were grown and then diluted to 1 OD before spiking in 100 μM metal. Given the relatively high culture density and short duration (4 hours) we could not see significant growth inhibition. We do measure cell death after metal uptake experiments (Supplemental Figure 8). However, when measuring cell growth with or without the presence of metals (Figure 2c, 3c, and Supplemental Figure 7), cells were instead diluted to < 0.1 OD and shaken in a

temperature-controlled plate reader for >24 hours. Growth viability and inhibition were much more apparent during their exponential growth phase than compared to the metal uptake studies at 1 OD_{600}. The distinction between these two protocols (metal uptake versus metal tolerance) have been written to be more clear in the Methods (culture density and viability assay methods are on page 29-30).

- Figure 2a) A description of Figure 2a is minimal, it is not stated that the upper part is flow cytometry, or that the lower part is ICP-AES measurements.

Thank you for pointing this out. A more detailed description of what experiment was performed when reporting data from Figure 2A has been added in the Results section and in the figure legend. In addition, a walkthrough on how this data was generated is now better described in the Methods section (page 28-29). Figure 2 along with its changes is copied below:

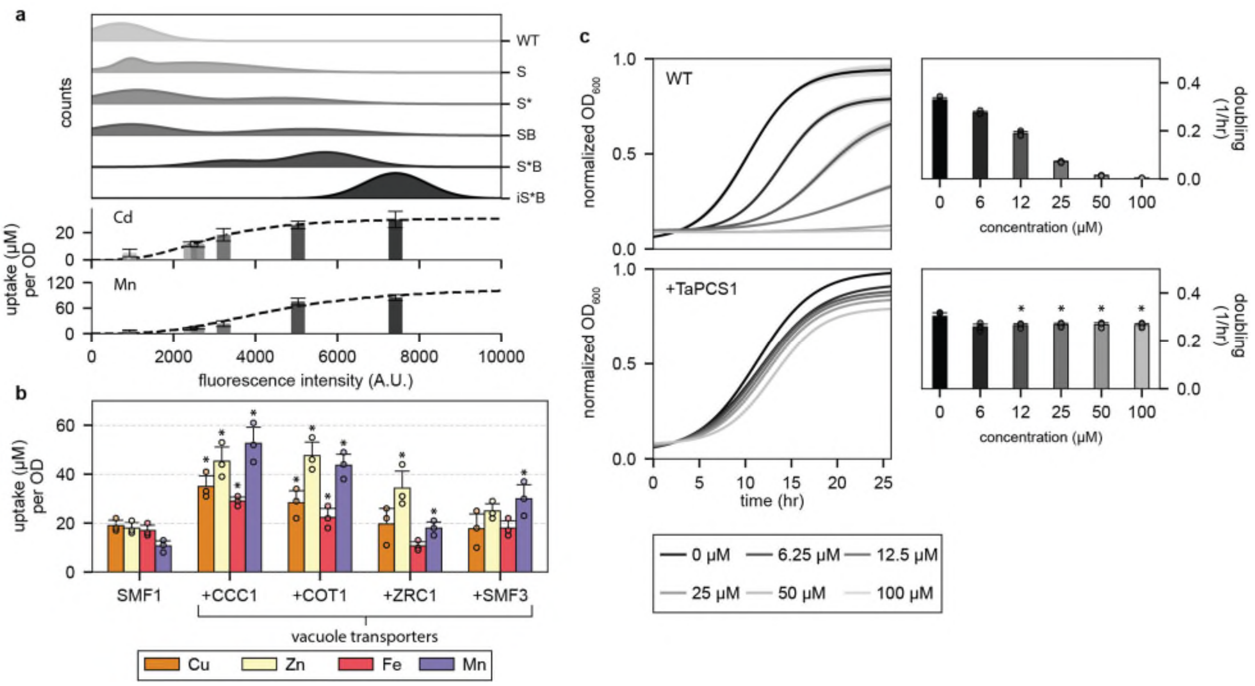

Figure 2 | Modifications in the metal trafficking pathway in yeast show enhanced metal uptake and tolerance for cadmium. a) Top subpanel shows the population distribution of SMF1 variants measured with fluorescently labelled V5-tag using flow cytometry. The weighted average of the fluorescent intensity corresponds to the placement of the lower subpanel bar charts which represent the level of metal uptake for that strain. Increasing expression levels of SMF1 correlate to increased metal uptake of cadmium or manganese; however, up to a certain point indicated by the plateau in uptake. **b**) Expressing vacuole transporters CCC1, COT1, ZRC1, and SMF3 in addition to SMF1 enhanced metal uptake. Asterisk above bar charts represent significant increase in uptake compared to SMF1 ($p < .05$). **c**) Constitutively expressing wheat phytochelatin synthase, TaPCS1, conferred heavy metal tolerance against cadmium. Asterisk above bar charts represent significant changes in growth rates compared to WT ($p < .01$). For all data, the mean ± s.d. of three replicates are shown.

- Figure 3 d: Titration curve of manganese and cadmium competition: The subfigure is mislabeled in the figure legend as Figure 3c. Units are missing from the Y-axis, an explanation of the process is missing from the figure text as well as no description is evident in the Materials and Methods section of how this was performed.

We have fixed the labeling typo in Figure 3. The figure has been modified to make room for the y-axis label. An explanation of what Figure 3d represents, and a brief description of how the data was collected has been added to the figure legend. In addition, a more thorough explanation on how the experiment was performed, data analyzed, and figure representation has been better described in the Methods section (page 27).

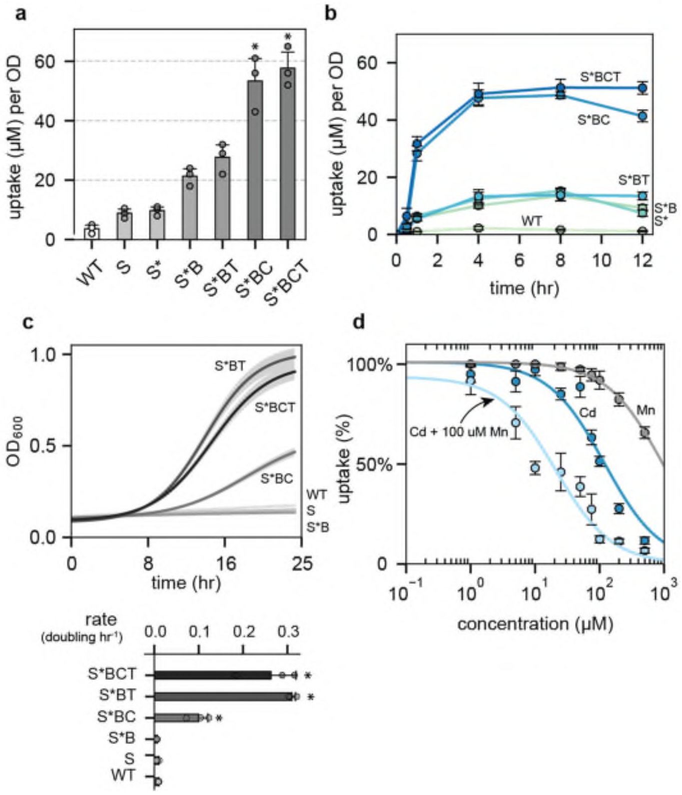

Figure 3 | Combining membrane transporter SMF1 and vacuole transporter CCC1 with TaPCS1 improved uptake capacity and metal tolerance. a) SMF1 (S) and its modifications (S* and ΔBSD2 as B) along with vacuole transporter CCC1 (C) and metal resistance enzyme TaPCS1 (T) incrementally enhanced cadmium uptake. Asterisk above bar charts represent significant increase in cadmium uptake when compared to WT ($p < .01$) . **b**) Combinations of S*, B, C, and T showed changes in uptake rate, capacity, and metal retention over 12 hours of metal incubation. **c**) In the presence of 100 μM cadmium, the growth rate is rescued with the addition of CCC1 and furthermore with TaPCS1. Subfigure below represents the doubling time of each strain. Asterisk to the side of bar charts represent significant increase in growth rate compared to WT ($p < .01$). **d**) S*BCT strain was titrated against cadmium, manganese, or cadmium in the constant presence of 100 μM manganese (x-axis). Metal uptake experiments was performed at varying concentrations from 1 to 100 μM, metal content analyzed using ICP, and values reported

as percent uptake. S*BCT showed a higher preference for manganese than cadmium, with cadmium uptake being dramatically reduced in the background presence of 100 μM manganese (light blue curve). For all data, the mean $\pm$ s.d. of three replicates are shown.

- Figures 2c, 3a+b, 5d: several text segments refers to the significant difference or lack of significant difference between samples, however no indicators of statistical significance are evident in the paper.

A two-tailed student t-test was performed for all samples which were declared significant in the manuscript ($p < .05$; or $p < .01$). Reported p-values have been added in the results section next to statements declaring significance. The description of the statistical test, and rationale for choosing this test, has been added to the Methods section (page 32).

- Line 141-143 and Supplementary Figure 2/Figure 7: In a large part of the work, measurements of metal uptake/optical density is converted into a measure of mg metal /grams of dry weight and this is used among other things to relate to the status of hyperaccumulator. The method used for these measurements is described differently in the figure text and in the Materials and Methods section. It is doubtful whether weight/OD correlations can be transferred between lines overexpressing transporters and, in addition, accumulating heavy metals. In the view of this reviewer this wild type weight/OD correlation seems too weak to use for calculating actual metal concentrations in the transformant yeast lines.

We looked back at our writing and attempted to better align the method description in the Methods section with the figure legend of Supplemental Figure 3 (labels have been reordered). We hope that the description is more consistent.

We focused on reporting units of μM of metal removed per cell culture OD (μM/OD); however we also wanted to compare our results with current plant literature values which report mg of metal removed per plant dry weight (mg/gDW). Unfortunately, there were several difficulties measuring yeast dry weight directly. The first was the volumes we were using which were < 10 mL for experimental metal uptake measurements. In these volumes, the lyophilized yeast were miniscule and difficult to transfer to a scale (they would flake, stick to the tube, or be effected by static). Also, weights were in the milligram to submilligram range making measurements difficult and often effected by noise. We were reluctant to scale experiments to 100 mL to 1 L because we would have to equally scale the amount of toxic metals used and would be responsible for waste disposal. The second difficulty would be to measure dry weight of every sample for every strain. More than 25 different strains were used (membrane transporters ZRT1, ZRT2, …; vacuole transporters SMF3, CCC1, …; and their combinations) which would make measurements extremely long and difficult if all had to be scaled up, lyophilized, and weighed in order to convert to mg/gDW for reporting sake. Overall, this method was intended to increase screening and experimental throughput. As not all strains were hyperaccumulators, we focused on this method to separate the positive strains for metal accumulation.

Overall, given our experimental limitations this was the best we could achieve to convert our measurements of μM/OD to mg/gDW. This effort was to help readers compare our

results with current hyperaccumulating values reported in the plant biology field. Therefore, we used an OD to weight correlation to map our measurements of metal uptake. Without this correlation the relationship between weight of metal uptake per dry weight of yeast would be nearly impossible given our experimental volumes. We also chose WT for the correlation because we wanted to use a single reference point (i.e. the same ratio) for all the engineered strains used in this manuscript.

Also, this method has been used in the past giving OD to gDW values between 0.36 – 0.6 for several yeast strains. (https://aem.asm.org/content/69/4/1990.short, https://bmcsystbiol.biomedcentral.com/articles/10.1186/1752-0509-6-49, https://bionumbers.hms.harvard.edu/bionumber.aspx?id=111182, https://www.researchgate.net/post/How_do_you_correlate_OD_measurements_with_cell_dry_weight)

To convince ourselves and the reader, we performed the experiment again with the fully engineered S*BCT strain and got a similar correlation between OD and dry weight. The figure is copied below.

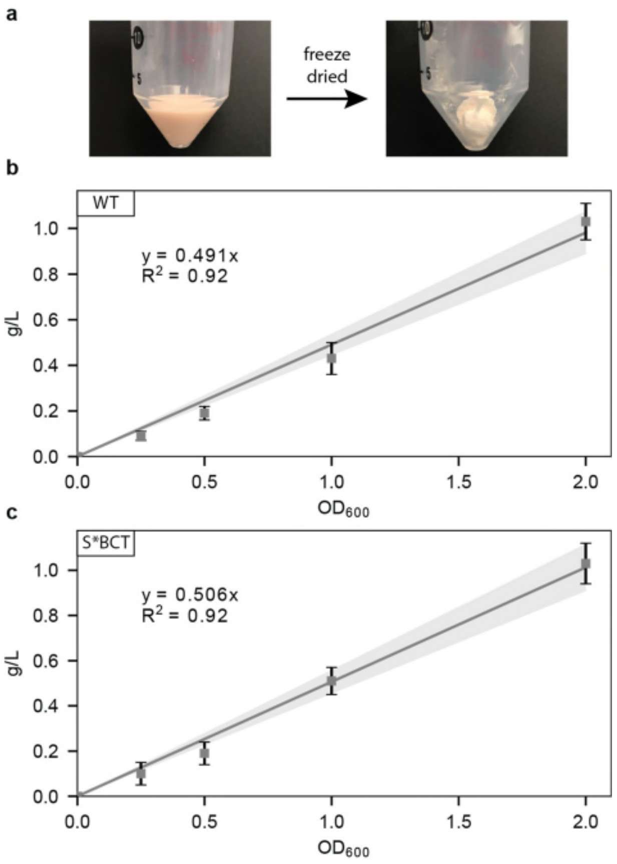

Supplemental Figure 3 | Correlating culture optical density (OD_{600}) to grams of culture dry weight (gDW). a) Cells were grown to the appropriate OD_{600}, washed, pelleted, and freeze-dried to obtain culture dry weight per culture volume. Masses were weighed on a precision scale with

hundredths of milligram resolution. A line of best fit with intercept at 0 was performed to obtain a correlation between OD_{600} and gram of dry weight per culture volume. **b**) Relationship for WT was 1 OD_{600} : 0.491 $\pm$ 0.05 mg of gDW per liter. **c**) Relationship for engineered S*BCT was 1 OD_{600} : 0.506 $\pm$ 0.1 gDW per liter. The difference in correlation between WT and S*BCT was negligible. For all data, the mean $\pm$ s.d. of three replicates are shown.

- Supplementary Figure 1/ Figure 6: the figure shows microscope images of yeast cells labeled with fluorescent antibodies to confirm expression of inserted transporters, however, the seemingly cytosolic localization of the tagged molecules underlines the need for a negative control in order to demonstrate that what is tagged is actually the membrane proteins in question.

Absolutely, this has been done and has been added to Supplemental Figures 1 and 5 (Supplemental Figures have been relabeled). The negative controls show little non-specific binding or auto fluorescence when compared to the experimental samples.

We acknowledge in the text that some over-expressing strains have punctate or non-uniform expression. We have added in the Results section that these observations were also used as another selection criteria for choosing SMF1 versus other transporters. For example, unlike FTR1 which showed punctate and non-uniform expression, SMF1 on the other hand showed good overall expression. These observations were further supported when SMF1 expression increased with subsequent modifications (Figure 2A and Supplemental Figure 4)

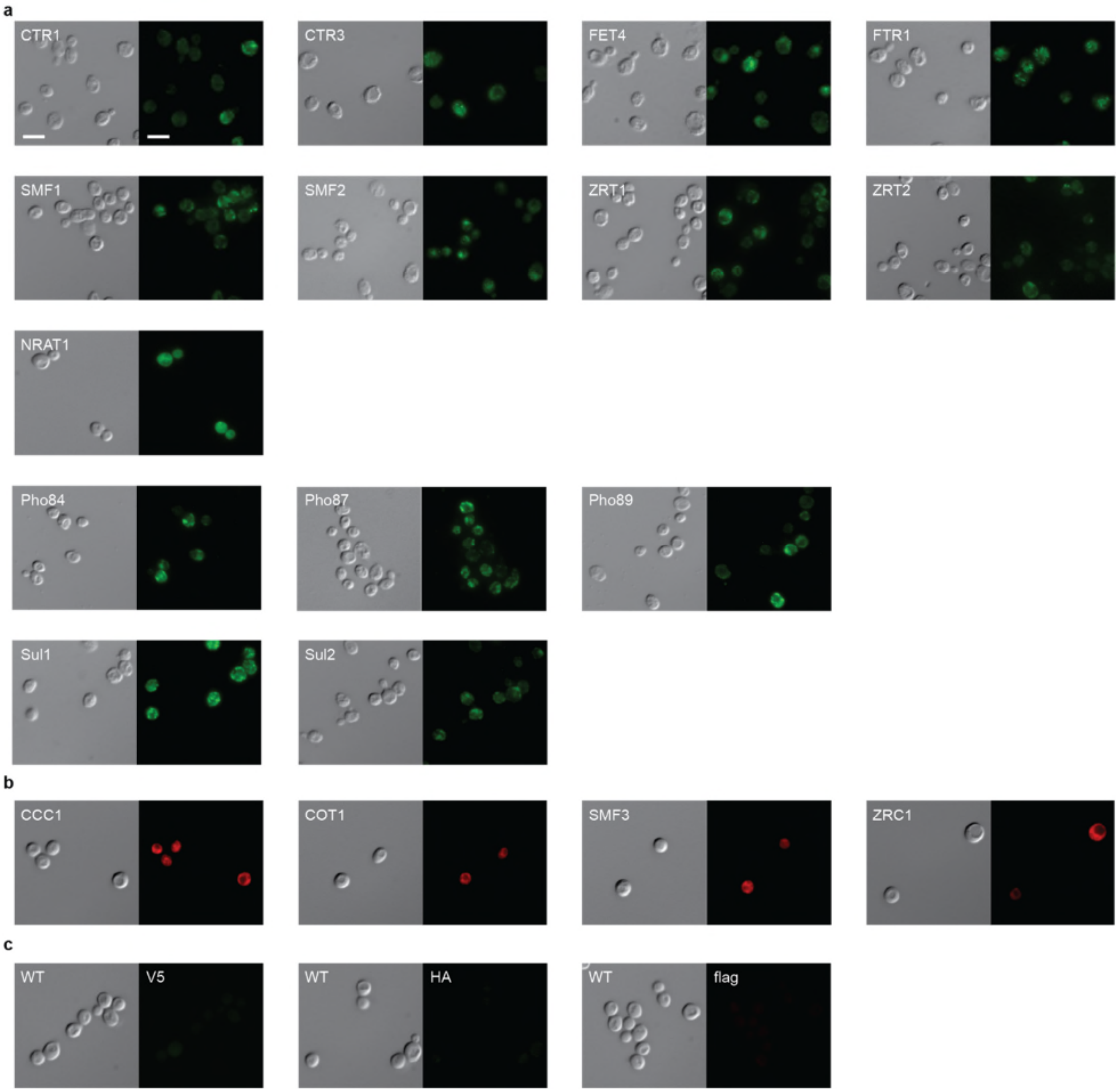

Supplemental Figure 1 | Fluorescently stained membrane and vacuole transporters to visualize overexpression. **a**) Membrane transporters CTR1, CTR3, FET4, FTR1, SMF1, SMF2, ZRT1, ZRT2 and NRAT1 were fused with a C'-terminus V5 tag and stained. Pho84, Pho87, Pho89, and Sul1 and Sul2 were fused with a C'-terminus HA tag and stained. **b**) Vacuole transporters CCC1, COT1, SMF3, and ZRC1 were fused with a C'-terminus flag tag and stained. **c**) Negative controls of WT were stained with identical antibodies targeting V5, HA, and flag tag in parallel with the transporters already described. No noticeable background fluorescence was observed. All tags were labelled with the appropriate primary and secondary antibodies conjugated with either an Alex488 (green; membrane) or Alex647(far red; vacuole) dye. Scale bars represent 5 μm for all images.

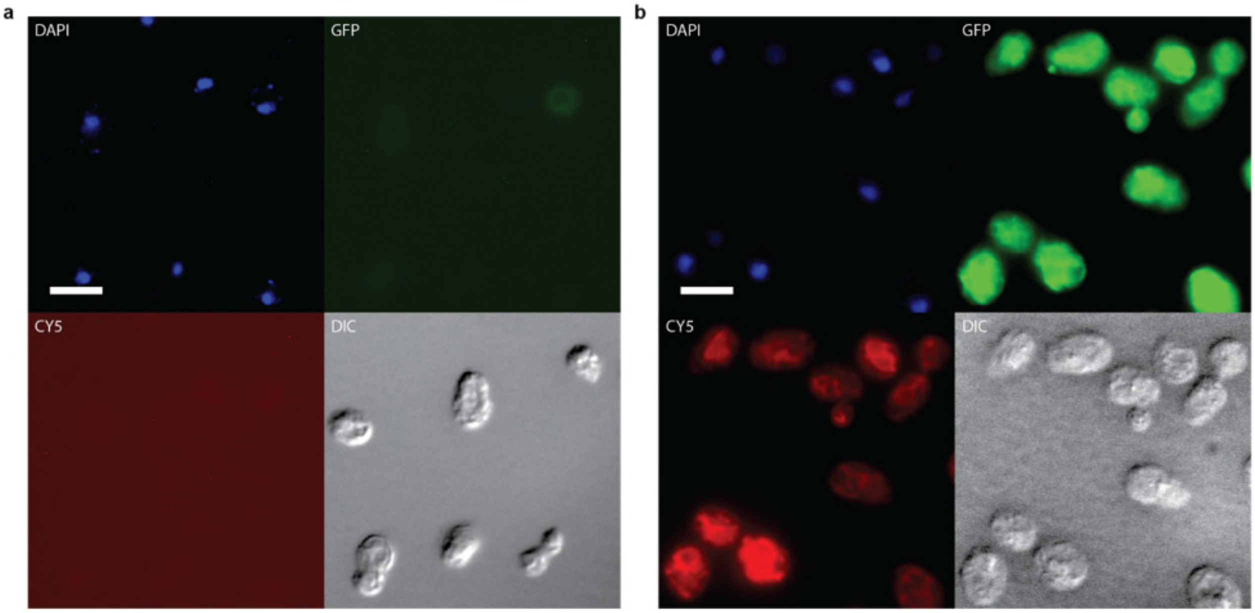

Supplemental Figure 5 | Fluorescently labelled SMF1 and CCC1 co-expressing strains. **a**) Fluorescent measurements of non-expressing WT strain as a control. **b**) SMF1* was fused with a C' terminus V5 tag, whereas CCC1 was fused with a C' terminus flag tag. Tags were stained with the appropriate primary and secondary antibodies conjugated with either an Alex488 (green; SMF1*) or Alex647(far red; CCC1) dye, respectively. Scale bars represent 5 μm for all images.

- Accession numbers: Throughout the paper, accession numbers for the genes implemented are missing. In addition, species denominators are largely missing and in one case where present is wrong (Line 206 states that TaPCS1 originates from rice/Oryza sativa)

We have embarrassingly realized that we mistakenly wrote that TaPCS1 originates from rice/Oryza sativa (actually, our other protein of interest Nrat1 does come from rice, which may have caused this mistake). We corrected that TaPCS1 comes from wheat.

Thank you for clarifying the reporting convention. To clarify, we have written the accession numbers for each gene when first introduced (e.g. SMF1 (#P38925)).

Reviewer #3 (Remarks to the Author):

Contamination of soils and water by toxic metals is widespread and of growing concern. Various bioremediation approaches may be used to clean up polluted soils but the progress of developing plants that hyper-accumulate metals has been slow. In this study, the authors used baker's yeast to create strains that hyper-accumulate metals. The strategy was based on: 1) increased expression of metal importers; 2) increased expression of proteins that transport metals into vacuoles; 3) expression of cytosolic metal-binding proteins. The authors then developed a screen to identify metal hyper-accumulator strains expressing forms of SMF1 with altered substrate specificity.

Overall, this is an interesting study that systematically examines the combined effect of gene overexpression (points 1-3 above) on metal hyper-accumulation (and cell survival). The novelty of this part of the manuscript is limited to the simultaneous co-expression of different genes already known individually (or in some cases combined 2 by 2) to contribute to metal uptake. The most novel aspects of the study includes 1) the density-based screening approach to identify metal hyper-accumulators coupled to colorimetric assays for metal specificity and 2) the generation of SMF1 variants that appear to have altered substrate specificity. However, the claim that these SMF1 variants have altered substrate specificity is not supported by solid biochemical data (e.g. quantification of protein amounts in the plasma membrane and/or reconstitution experiments). Efforts should be made to cement this claim as engineering transporters for altered substrate specificity is a key aim of this work. Thus, the advance from previous studies appears moderate when it comes to novel mechanistic understanding and overall impact.

Specific comments
1. line 131: it is claimed that Nrat1 promotes selective uptake of aluminium. This claim is not supported by data (only Al has been tested as substrate).

Thank you for pointing this out. The data for Nrat1 uptake of Cu, Zn, Fe, and Mn were measured but not shown in the manuscript. This data is now represented and added to the supplemental figures as Supplemental Figure 2 (Figure numbers have been re-labeled).

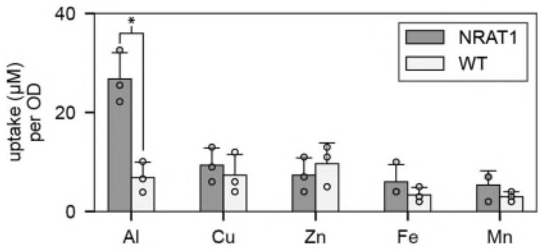

Supplemental Figure 2 | Nrat1 Al uptake compared to other transition metals used in this study. Metal uptake experiments for Nrat1 was performed with Al, Cu, Zn, Fe, and Mn and compared against non-expressing WT strain. Asterisk above bar chart represent significant uptake when compared to WT ($p < .05$).

2. line 136: the statement ‘preferential accumulation of aluminium’ is not supported by experimental data.

From Supplemental Figure 2, copied above, we have added data to support the “preferential accumulation of aluminium” claim by testing Nrat1 on Mn, Zn, Fe, as well as Cu uptake. The results show a significant difference in aluminum uptake for NRAT1 compared to WT ($p < .05$), which is not true for the other transition metals ($p >> .05$).

3. Please add p-values for significance for the metal accumulation assays (e.g. Fig 1b, 2b, 5b etc) and adjust the text accordingly.

We apologize for omitting p-values in our text when claiming significance. p-values for all figures ($p < .05$ or $p < .01$) have been added in the Results section when reporting significance. A description of the statistical test used has been added in the Methods section (page 32).

4. line 199: GSH functions as a metal chelator in yeast on its own (and not only as a constituent of phytochelatin) – please mention this fact.

Thank you for the clarification. This fact has been stated when discussing GSH and phytochelatins, the statement is copied below (on page 10).

Yeast are able to produce glutathione via the GSH pathway, which naturally protects yeast from accumulation of toxic metals.

5. line 206: TaPCS1 – Ta stands for Triticum aestivum (wheat) and not rice. Please correct.

We are extremely embarrassed to have made this mistake! This has been corrected, and thank you for pointing this out.

6. line 2249-251: the sentence ‘Multi-aligning the…. revealed region…’ is unclear. Please clarify what was revealed (e.g. highest homology?).

Apologies for the unclear wording, the intention of the multi-alignment was to locate the precise transmembrane (TM) regions of SMF1 which is still unknown given no definitive crystal structure (although the regions have been hypothesized). However, given that these regions are known for its homologues (e.g. DraNramp, and ScaDMT), a multi-alignment with these two structures and an assorted list of the top 100 homologues curated by Pfam helped pinpoint the TMs of SMF1 (shown in Figure 4A).

We also performed a theoretical prediction of SMF1 TM regions using TMHMM (http://www.cbs.dtu.dk/services/TMHMM-2.0/). This method was only used as an internal reference for us and not reported in the manuscript.

If interested, the figure is copied below; the shaded maroon areas represent the TM regions of SMF1.

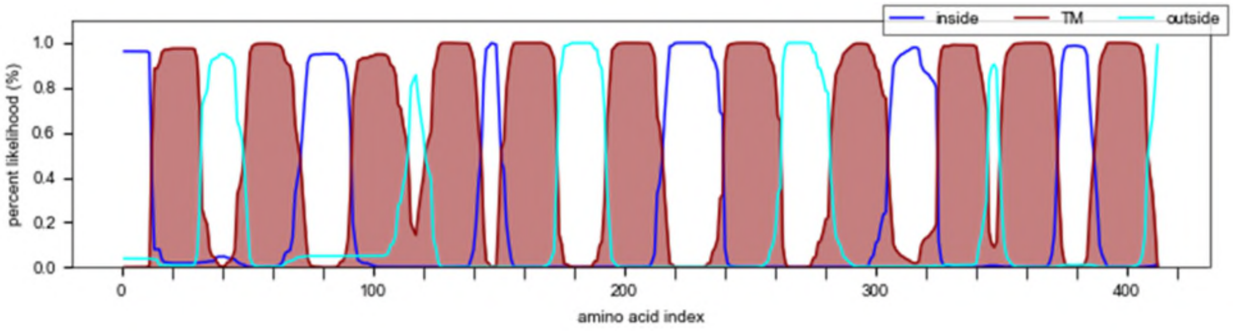

7. line 298: Please explain the experimental set-up: was it iterative rounds of growth to saturation?

We have clarified this experimental set-up in the Methods section (page 27-28). One round consists of a normal metal uptake experiment where 1 OD_{600} of induced cells is incubated with 100 μM of metal (Cd, Sr, or a combination with Mn). After 4 hours, the supernatant is collected and transferred to a fresh new culture of induced cells diluted to 1 OD_{600}, and the metal uptake experiment is performed again. The supernatant is repeatedly collected and transferred to a freshly induced culture, and this procedure is performed up to 4 times. The edited text is copied below:

Iterative metal uptake experiments was performed by taking the supernatant of a previous metal uptake experiment, and transferring the supernatant directly into a freshly induced culture normalized to 1 OD_{600}. Uptake was performed for 4 hours, and supernatant transferred iteratively to a fresh new culture up to 4 times. At each iteration the supernatant was sampled and measured using ICP to calculate the metal uptake per round.

8. line 300: un-engineered S* - S* is a mutated form of SFM1. Please correct.

Thank you for spotting this typo, this has been corrected.

9. Fig 2: It is unclear what the top panel in Fig 2a shows. Fig 2c lacks label of the y-axis.

Apologies for the mislabelings. The top subfigure in Figure 2a shows increasing fluorescence intensity of SMF1 variants tagged with a fluorescence antibody on the C' V5-tag. Measurements were performed using flow cytometry. The weighted-average of these intensity values is mapped to the amount of cadmium uptake (the lower subfigure bar chart in Figure 2a), which shows a trend in metal uptake as a function of SMF1 expression. A more detailed discussion of the figure has been added to the Results section and figure legend. The Methods section has been updated to also include a more thorough outline of how this experiment was performed (page 28-29). The y-axis for Figure 2c has been added. Our mistake for the omission, and thank you for pointing this out. A new Figure 2 and figure legend is copied below.

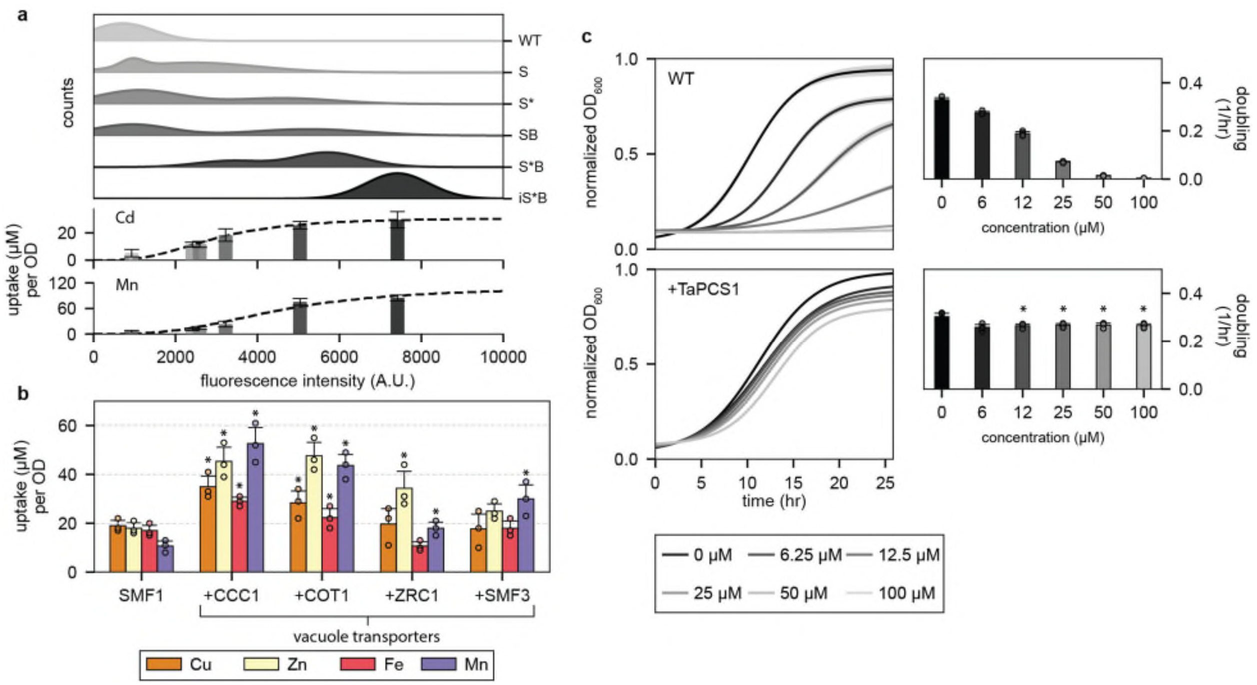

Figure 2 | Modifications in the metal trafficking pathway in yeast show enhanced metal uptake and tolerance for cadmium. a) Top subpanel shows the population distribution of SMF1 variants measured with fluorescently labelled V5-tag using flow cytometry. The weighted average of the fluorescent intensity corresponds to the placement of the lower subpanel bar charts which represent the level of metal uptake for that strain. Increasing expression levels of SMF1 correlate to increased metal uptake of cadmium or manganese; however, up to a certain point indicated by the plateau in uptake. **b**) Expressing vacuole transporters CCC1, COT1, ZRC1, and SMF3 in addition to SMF1 enhanced metal uptake. Asterisk above bar charts represent significant increase in uptake compared to SMF1 ($p < .05$). **c**) Constitutively expressing wheat phytochelatin synthase, TaPCS1, conferred heavy metal tolerance against cadmium. Asterisk above bar charts represent significant changes in growth rates compared to WT ($p < .01$). For all data, the mean ± s.d. of three replicates are shown.

10. Fig 4a: Please explain what black and red letters denote.

The distinction between the black and red coloring has been added to the figure legend. How these colors and alignment were derived is more thoroughly described in the Methods section (page 24), copied below.

The resultant multi-aligned file was visualized using ESPript (http://espript.ibcp.fr/ESPript/ESPript/) with reference sequence taken from PDB entry 5KTE to help indicate regions with secondary structure. Red highlighted amino acids indicate highly conserved regions with similarity scores > 0.7. All other amino acids are colored black.

11. Fig 5b: Please indicate the strain background in the legend.

Good point, this has been added to the figure legend.

12: Fig S1 (labelled Fig 6 in the submitted MS): The fluorescent images do not show clear/uniform plasma membrane localization of the transporters. Instead, the fluorescent signal is often visible as dotted structures inside the cell. Thus, using total fluorescence as a measure of functional protein expression is clearly problematic.

We realized some of the transporters that were over-expressed produced punctate patterns and were not uniformly localized on the cell surface. This seemingly cytosolic localization was compared to a negative control of non-expressing WT strain, which has been added to Supplemental Figure 1. The intention of the fluorescent imaging was to help identity transporter candidates for further engineering. Therefore, the degree of transporter localization was a factor in determining which transporter we would (or could) engineer. For example, FTR1, and SMF2 had punctate patterns as mentioned, so they were not considered as possible candidates for further engineering. Thus we decided on SMF1 as a template transporter to improve on, given its uniform expression, promiscuous metal uptake characteristics, and the vast literature on its function. We have included your concern of the irregular fluorescent patterns of some transporters in the Results and discussed that this characteristic was productively used to select SMF1 as a base transporter. In addition, the subsequent fluorescent images (Supplemental Figure 4) highlights the improve expression uniformity and quality of SMF1 after subsequent modifications. Sentences addressing this concern has been added to the Results (page 8) and copied below.

Another selection criteria was SMF1's relatively consistent membrane-localized expression as observed under fluorescent microscopy. When compared to FTR1 for example, expression was punctate and non-uniform, suggesting poor localization due to over-expression (**Supplemental Figure 1a**). Therefore the inability to over-express and localize transporters eliminated candidates for further engineering.

Reviewer #4 (Remarks to the Author):

The Sun et al. paper is an original study aiming to engineer budding yeast, the laboratory model widely used to characterize plant metal transporters, toward an hyperaccumulator microorganism that could be used for bioremediation purposes.
The main functional adaptations of known plant hyperaccumulators were combined and successfully applied to the yeast model (improved expression of metal importer at the cell surface combined to overexpression of vacuole metal importer and synthesis of intracellular organic metal chelator that can be stored in the vacuole) to demonstrate increased capacity for Cd accumulation in yeasts that remained viable.
An approach to select for enhanced accumulation of toxic heavy metals (Cd, Sr) based on semi-targeted mutagenesis of the yeast metal importer Smf1 is presented. It uses rate-zonal density centrifugation to enrich heavier cells with stronger metal load. The resulting metal uptake properties of two Smf1 variants are described, which catalyze either preferential accumulation of Cd vs Mn, i.e., metal preference opposite to Smf1 native affinity, or reduced Mn uptake but neoaccumulation of Sr. In both cases novel properties resulted from targeted mutations and were accentuated by subsequent random mutagenesis.
Hence a proof of principle is suggested that yeast cells can be engineered to accumulate toxic metals at levels similar to those reported for plant hyperaccumulators.
After significant revision this study should foster interest in developing yeast hyperaccumulator strains needed for bioremediation approaches in industrial settings such as the textile industry.

Methodological criticisms:
Quantitation of metal hyperaccumulation: Growing yeast that were pre-induced to express genes of interest, were exposed to metals for 4h then cell pellets were processed for ICP-AES analyses. Then the relationship established between OD of WT yeast cultures and dry weight of the lyophilized cells was used to calculate metal accumulation (mg per kg of dry weight) for the mutant studied.
This approach raises several questions: For instance, do engineering processes alter the relationship between yeast OD and dry weight? Also, yeast cell wall constitutes a significant portion of dry weight and it is unclear whether external metal adsorption on yeast has been distinguished from metal internalization. Answering such questions would strengthen the claim that engineered yeasts behave as hyperaccumulators.

We focused on reporting units of μM of metal removed per cell culture OD (μM/OD); however we also wanted to compare our results with current plant literature values which report mg of metal removed per plant dry weight (mg/gDW). Unfortunately, there were several difficulties measuring yeast dry weight directly. The first was the volumes we were using which were < 10 mL for experimental metal uptake measurements. In these volumes, the lyophilized yeast were miniscule and difficult to transfer to a scale (they would flake, stick to the tube, or be effected by static). Also, weights were in the milligram to submilligram range making measurements difficult and often effected by noise. We were reluctant to increase the scale of our experiments to 100 mL to 1 L because we would have to equally scale the amount of toxic metals used and be responsible for waste disposal. The second difficulty would be to measure dry weight of every sample for every strain. More than 25 different strains were used (membrane transporters ZRT1, ZRT2, …; vacuole transporters SMF3, CCC1, …; and their

combinations) which would make measurements extremely long and difficult if all had to be scaled up, lyophilized, and weighed in order to convert to mg/gDW for reporting sake. Overall, this method was intended to increase screening and experimental throughput. As not all strains were hyperaccumulators, we focused on this method to separate the positive strains for metal accumulation.

Overall, given our experimental limitations this was the best we could achieve to convert our measurements of μM/OD to mg/gDW. This effort was to help readers compare our results with current hyperaccumulating values reported in the plant biology field. Therefore, we used an OD to weight correlation to map our measurements of metal uptake. Without this correlation the relationship between weight of metal uptake per dry weight of yeast would be nearly impossible given our experimental volumes. We also chose WT for the correlation because we wanted to use a single reference point (i.e. the same ratio) for all the engineered strains used in this manuscript.

In addition, this method of correlating OD to gDW has been used in the past giving OD to gDW relationships between 0.36 – 0.6 for several yeast strains. (https://aem.asm.org/content/69/4/1990.short, https://bmcsystbiol.biomedcentral.com/articles/10.1186/1752-0509-6-49, https://bionumbers.hms.harvard.edu/bionumber.aspx?id=111182, https://www.researchgate.net/post/How_do_you_correlate_OD_measurements_with_cell_dry_weight)

To convince ourselves and the reader, we performed the experiment again with the fully engineered S*BCT strain and got a similar correlation between OD and dry weight. The figure is copied below (supplemental figures have been relabeled. It is now Supplemental Figure 3).

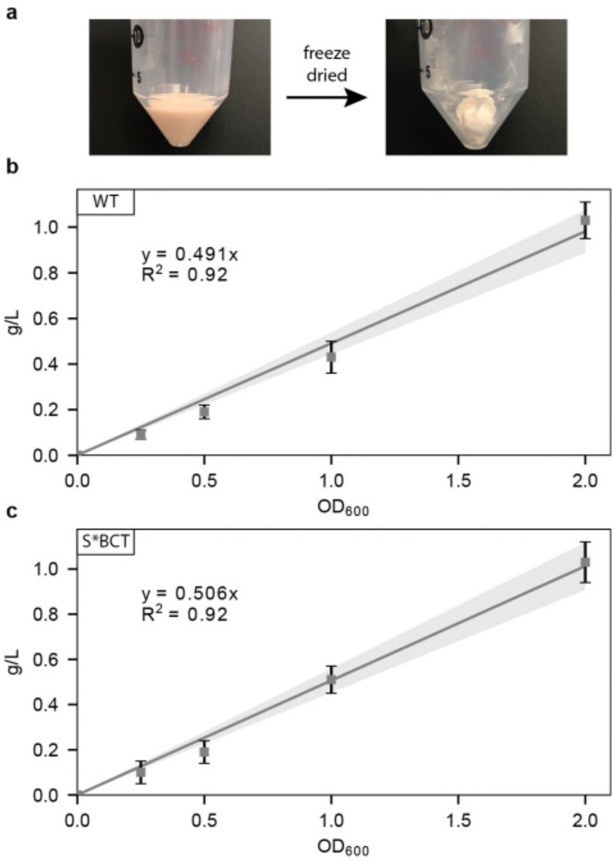

Supplemental Figure 3 | Correlating culture optical density (OD_{600}) to grams of culture dry weight (gDW). **a**) Cells were grown to the appropriate OD_{600}, washed, pelleted, and freeze-dried to obtain culture dry weight per culture volume. Masses were weighed on a precision scale with hundredths of milligram resolution. A line of best fit with intercept at 0 was performed to obtain a correlation between OD_{600} and gram of dry weight per culture volume. **b**) Relationship for WT was 1 OD_{600} : 0.491 $\pm$ 0.05 mg of gDW per liter. **c**) Relationship for engineered S*BCT was 1 OD_{600} : 0.506 $\pm$ 0.1 gDW per liter. The difference in correlation between WT and S*BCT was negligible. For all data, the mean $\pm$ s.d. of three replicates are shown.

As for the second comment, from our understanding we made it unclear whether or not metal was truly translocated *into* the cell, rather than being non-specifically bound to the cell wall. Our method to calculate metal uptake was to measure the metal content of the supernatant to then calculate the amount of metal removed by the cells. In order to take into consideration cellular non-specific metal uptake, a WT sample (non-expressing strain) was processed under similar conditions and measured for metal uptake. These controls are shown at the end of each bar chart in Figure 1 b-d, Figure 2a, Figure 3a,b and Figure 5b. These WT controls show minimal non-specific absorption of metal on or into the cell. For example, the metal uptake of cadmium for WT was 3.7 $\pm$ 1.3 μM/OD, whereas for our engineered strain S*BCT cadmium uptake was 57.7 $\pm$ 5.4 μM/OD, ten times more (Figure 3A).

As well, in each experiment a sample without any cells was measured to test for non-specific metal binding to the sample tube or from loss during the experimental procedure. These values were negligible and did not affect our experiments. We have edited the Results and Methods section to add more detail on this experimental procedure (page 26-28).

Fluorescent visualization of over-expressed transporters (Suppl. Figs 1&4) does not allow one to discriminate the site of protein expression (plasma membrane or vacuole).

We realized some of the transporters that were over-expressed produced punctate patterns and were not solely localized to the cell surface. This seemingly cytosolic localization was compared to a negative control of non-expressing WT strain, which has been added to Supplemental Figure 1. The intention of the fluorescent imaging was to help identity transporter candidates for further engineering. Therefore, the degree of transporter localization was a factor in determining which transporter we would (or could) engineer. For example, FTR1, and SMF2 have punctate structures as mentioned, so they were not considered as possible candidates. Thus we decided on SMF1 as a template transporter to improve on, given its uniform expression, promiscuous metal uptake characteristics, and the vast literature on its function. We have included your concern of the irregular fluorescent patterns of some transporters in the text and discussed that this characteristic was used productively to select SMF1 as a base transporter. In addition, the subsequent fluorescent images (Supplemental Figure 4) highlights the improve expression uniformity and quality of SMF1 after subsequent modifications.

As an example of this correction, sentences addressing this concern has been added to the Results (page 8) and copied below.

Another selection criteria was SMF1's relatively consistent membrane-localized expression as observed under fluorescent microscopy. When compared to FTR1 for example, expression was punctate and non-uniform, suggesting poor localization due to over-expression (Supplemental Figure 1a). Therefore the inability to over-express and localize transporters eliminated candidates for further engineering.

l. 100: metal exporters, which could release metals out of the vacuole or out of yeast cells are only briefly mentioned (l. 225: antiporters?, and in the discussion). They are not presented in Fig. 1a. The activity of Smf3 in the assays presented (Fig. 2b and Suppl. Fig. 5) should be clarified relatively to the role of vacuole importers; in the case of iron, this could require co-examining the role of Fre6p as well.

We have included in the Figure 1A illustration both membrane and vacuole antiporters and mentioned their significance in the text and figure legend. We also clarified the role of SMF3 as a vacuole importer. Modified Figure 1 with edited figure legend is copied below.

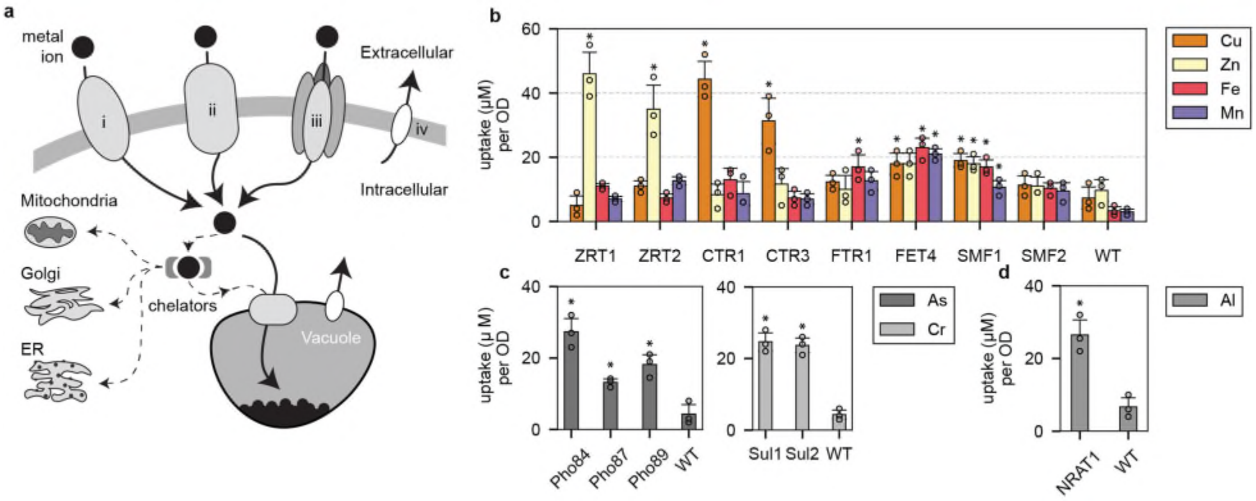

Figure 1 | A variety of metal transporters can be used to selectively internalize heavy metals into yeast. a) A simplified schematic of metal transport in a eukaryotic cell. Membrane transporters can be divalent metal transporters (i), permeases (ii), metal transporters that are modified or found to have auxillary metal transport function (iii), or antiporters which are used to remove excess metals out of the cell (iv). **b**) Bar coloring indicates metal measured, with over-expressed transporter labeled on the x-axis. Values are reported in μM of metal uptake normalized per yeast culture density (μM/OD). Yeast metal transporters for zinc (ZRTs), copper (CTRs), iron (FTRs and FETs), and manganese (SMFs) were overexpressed and studied for metal hyperaccumulation. A WT strain was also tested in parallel for each metal to measure non-specific metal uptake. **c**) The same study was performed for phosphate and sulfate permeases (PHOs, and SULs) which showed transport of arsenate and chromate, respectively. **d**) The Nrat1 transporter, previously shown to uptake trivalent metals in certain strains of rice, was expressed and showed aluminum(III) transport. Asterisk above bar charts represent significance increase in uptake compared to WT ($p < .05$) for strains mentioned in the text. For all data, the mean ± s.d. of three replicates are shown.

II. Titration of uptake: presentation of the data renders interpretation difficult (Fig. 3b, Fig. 5c); how could possibly maximal uptake occur when adding metal at the lowest dose? Figure legend should mention that x axis represents test metal concentration (Mn or Cd, Cd being used alone or in the presence of 100 uM Mn).

Thank you for pointing out this confusion. We have mentioned in the figure legend that the x-axis represents the concentration of metals (Mn, Cd, or both) and provided more white-space to include additional texts to point out the y-axis in Figure 3d. The lowest metal dose added was 1 μM, the lowest sensitivity on the ICP-AES. The y-values represent percent metal uptake, rather than total uptake. So at 1 μM, most of the metals are removed from the culture giving a high percent uptake. Whereas adding an excess of 1 mM of metal did not give a high percent uptake as the excess of metal reduced this percent value (however, uptake was greater than that at 1 μM)

Edited Figure 3 and figure legend is copied below for reference:

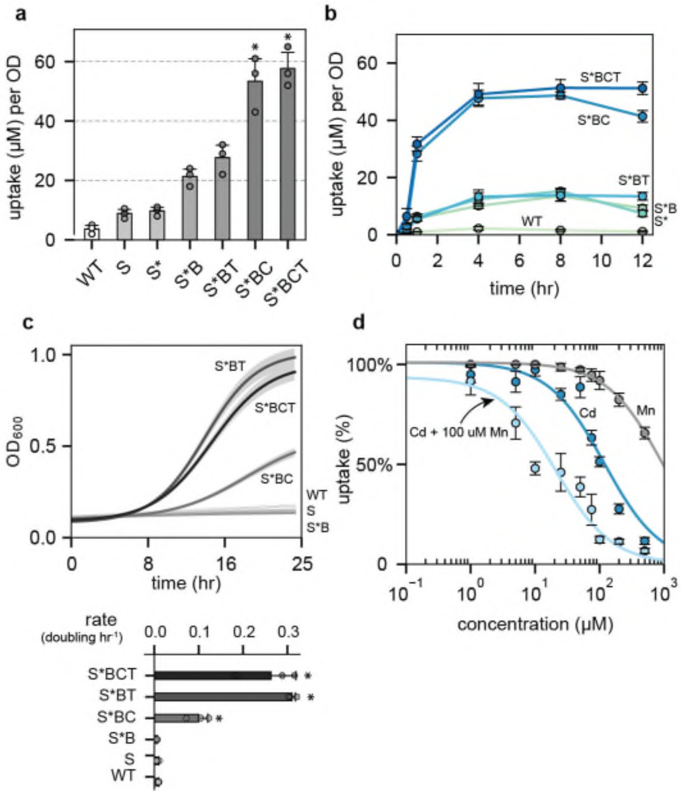

Figure 3 | Combining membrane transporter SMF1 and vacuole transporter CCC1 with TaPCS1 improved uptake capacity and metal tolerance. a) SMF1 (S) and its modifications (S* and ΔBSD2 as B) along with vacuole transporter CCC1 (C) and metal resistance enzyme TaPCS1 (T) incrementally enhanced cadmium uptake. Asterisk above bar charts represent significant increase in cadmium uptake when compared to WT ($p < .01$) . **b**) Combinations of S*, B, C, and T showed changes in uptake rate, capacity, and metal retention over 12 hours of metal incubation. **c**) In the presence of 100 μM cadmium, the growth rate is rescued with the addition of CCC1 and furthermore with TaPCS1. Subfigure below represents the doubling time of each strain. Asterisk to the side of bar charts represent significant increase in growth rate compared to WT ($p < .01$). **d**) S*BCT strain was titrated against cadmium, manganese, or cadmium in the constant presence of 100 μM manganese (x-axis). Metal uptake experiments was performed at varying concentrations from 1 to 100 μM, metal content analyzed using ICP, and values reported as percent uptake. S*BCT showed a higher preference for manganese than cadmium, with cadmium uptake being dramatically reduced in the background presence of 100 μM manganese (light blue curve). For all data, the mean $\pm$ s.d. of three replicates are shown.

III. Isolation of metal hyperaccumulating mutants by rate-zonal density centrifugation: yeast density refers to the weight of an individual cell per unit volume; rate zonal approach was deemed more appropriate by the authors than isopycnic centrifugation "as previous studies have shown that yeast maintain a relatively constant density despite external influences" (l. 269-270), so yeast mutants were separated according to their rate of sedimentation. That rationale is unclear to me, given that to maintain density a hyperaccumulating mutant would be expected to vary cell volume or shape; what consequence then on the correspondence between OD and dry weight measurements and accuracy of hyperaccumulation data? In that case wouldn't it be

preferable to relate dry weight to colony forming ability?

Very good point! In past studies, yeast are shown to have relatively constant densities. Our first screening design was actually based on isopycnic density centrifugation, as we thought the added mass from metal uptake would increase the density of mutated strains. However we did not see visible changes in migration between cells that did or did not uptake metals using isopycnic density centrifugation. But we did see visible changes in migration using rate-zonal density centrifugation. We chose rate-zonal density centrifugation because the rate of sedimentation is affected by particle mass *and* shape. Heavier and larger particles sediment quicker than smaller ones. Our hypothesis is that the added mass from the accumulated metals corresponds to an increase in volume, hence unchanging density, while increasing size and therefore sedimentation rate. The increase in volume may be possibly due to water absorption to maintain isotonicity from the increase in metal ion content. Therefore the added mass (from the uptaken metals) and size (from absorbed water) allow for greater segregation during rate-zonal density gradient centrifugation, as shown in Supplemental Figure 10.

We believe this effect does not change our calculations of dry weight. Also, in previous hyperaccumulator studies dry weight is typically measured before uptake. As in, the unit is used to determine how much dry weight is needed of a particular plant to accumulate a certain area of contaminants. Therefore our dry weight calculations were done on cultures without the addition of metal. A final thought would be that the added volume, possibly due to an increase in water content, the wet water weight is lyophilized away and not included in dry weight calculations.

We also show in Supplemental Figure 3 (copied above in the first response) that the OD to dry weight correlation remains relatively the same for the fully engineered S*BCT, suggesting that over-expression does not significantly impact the relationship between OD and yeast dry weight.

4. Functional characterization of the hyperaccumulating mutants: due to possible issues relating to OD variations between mutants it would be informative to compare the expression levels of mutant proteins (Fig. 5b).

Agreed. We have gone back to our mutant strains and performed a similar experiment as in Figure 2A and measured percent of expression populations using flow cytometry. The change in expression was not majorly effected by mutating the transporter. A new Supplemental 12 with figure legend is copied below. We would also like to note that all strains were normalized to 1 OD in our experiments to reduce the effect on OD variations when measuring metal uptake.

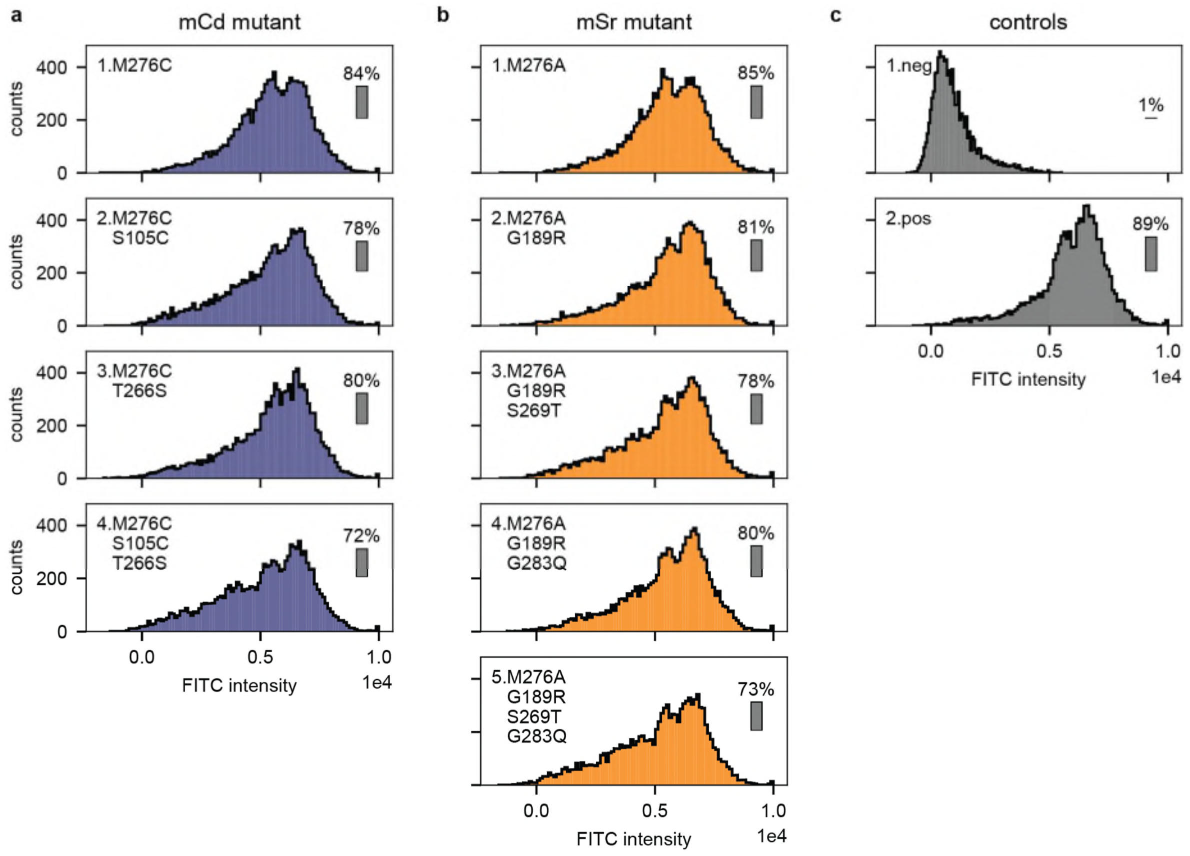

Supplemental Figure 12 | Effect of mCd and mSr mutations on SMF1 expression measured with flow cytometry. a) Progressive mutations (1-4; corresponding to numberings in **Figure** 5**b**) leading to mCd caused little change in transporter expression. **b**) Likewise, a slight reduction in mSr transporter expression was observed, but not significant. **c**) Negative (WT) and positive controls (S*BCT) were also measured to compare expression changes with non-engineered strains.

Only two mutants, each combining 2 neomutations, are described as a result of 4 rounds of selection pipeline. Are these the only mutants that were obtained? This would mean that less than 1 mutation was obtained per round of selection. If so, what can be deduced regarding the efficiency of the screening procedure? Otherwise it would be useful to indicate the overall number of mutants obtained per round of selection and whether mutation map within TM or intervening loops.

The number of mutations per round of selection seems low because of our stringent filtering process, and we also only show the best candidate per round (Figure 5a,b). The first filtering step was the density gradient centrifugation, which reduced millions of possible mutants into several hundred colonies which were plated. From there, tens of colonies were selected in a 96-well plate for the manganese competition assay. Finally, 4-6 of the best performers were examined with ICP and then sequenced. We will better clarify this process, and the number of mutants obtained per round in the Methods section (copied below, page 31).

… Collected [mutant] cells were plated onto 2-3 plates giving approximately 10-100 colonies each. After platting roughly 10-50 colonies, depending on the number of colonies formed, were picked in 100 μL SD-R cultures in a 96-well format and induced following the same protocol as before. Cells were diluted to 1 OD_{600} and spiked with 100 μM cadmium or strontium with the addition of 100 μM manganese and shaken for 4 hours. Plates were spun down at 900xg for 3 min and the supernatant was diluted 1:10 in ddH_2O and assayed using the modified manganese Hach detection kit described above. The top 4-6 Wells with the highest readings (most manganese remaining) were selected and plated again. Selected colonies were then subjected to a more thorough metal uptake ICP experiment and sequenced before re-introducing them into the screening pipeline.

Lastly, in several parts of the text the authors' statement is not clear. A few examples:
l. 67-68: what is the strict definition for Ni hyperaccumulator
Do reported values refer to dry weight of whole plant or specific parts that accumulate metals?

The strict definition for Ni has been added in that sentence (it is 1,000 mg/kg or 1% dry weight). We may have missed that element in the list, our apologies. The literature is quite diverse in quantifying dry weight in plants. Several protocols measure plant dry weight before metal uptake experiments, and use the dried biomass to soak up heavy metals. This is typically done when plants are harvested and biomass transported to a contaminated site. Others measure dry weight of the entire plant after several days to weeks in spiked soils. Others have specifically reported dry weight of just the leaves, shoots, or roots.

An example of whole-plant measured dry weight:
http://www.plantphysiol.org/content/134/2/716.short

An example of individual plant parts measured by dry weight:
https://link.springer.com/article/10.1023/B:PLSO.0000020956.24027.f2

l. 108: text refers to Supp. Fig. 1 that is instead labelled Figure 6; labelling should be corrected for all the supplemental data

Our apologies. All figure and table labels are now corrected and changes should also be reflected in the text as well. Also, all Figure numbers cited in this response have been updated to reflect these changes.

l. 300-302 vs l. 766-767: sentences in the text and figure legend have opposite meaning, hence difficult to evaluate the data presented

Figure 5 legend and corresponding text have been corrected. Thank you for pointing out such a detailed error.

The quality of presentation should be improved for some figures as well:
-Fig.1b is not easy to read, either present values from which WT levels were subtracted or a presentation like that used in Suppl. Fig. 5.

Here is our attempt

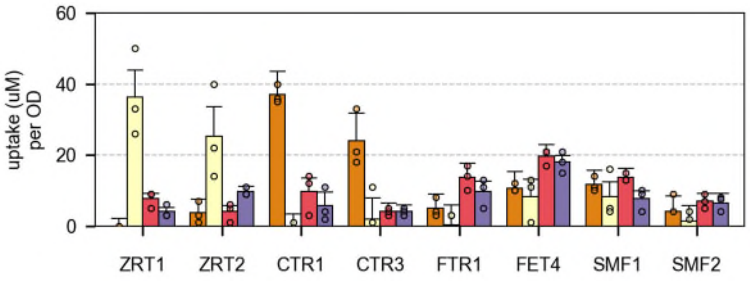

Our concern is that we lose the explicit information of the WT reference, which is used to show the baseline non-specific uptake of metals for non-expressing strains. Also, our significance test are done against the WT values, which would be good to show in the figures. We would also need to change the remaining figures to subtract the WT baseline values (Figures 1C-D).

-Fig. 3c and 3d: legends do not correspond.

This has been fixed. In addition, more information for figure legend 3D has been added to better describe the data presented. These changes are reflected on the Figure 3 copied above in a previous response.

-Fig. 5b and 5c: M276 or M267?

Thank you for pointing this out. This has been fixed, and the entire text has been double-checked to make sure M276 is used. We believe the figure legend had the only error.

Figure 5 legend is copied below to show the correction:

Figure 5 | Cadmium and strontium metal transport mutants were generated after 4 rounds of screening. a) Weblogos of SMF1 TM1,6 from Nramp multi-alignment are displayed, with cadmium and strontium mutations highlighted. Cadmium mutants had S105C, T266S, and M276C. Strontium mutants had G189R, S269T, M276A, and G283Q. **b**) Total metal uptake of 100 μM cadmium and manganese were measured to assess manganese interference. Cadmium mutant labeling corresponds to 1=M276C, 2=M276C+S105C, 3=M276C+T266S, and 4=M276C+S105C+T266S. Strontium mutant labeling corresponds to 1=M276A, 2=M276A+G189R, 3=M276A+G189R+S269T, 4=M276A+G189R+G283Q, and 5=M276A+G189R+S269T+G283Q. Strain background for all mutants were BCT. Asterisk above bar charts represent significant changes in both Cd and Mn uptake compared to un-mutated S*BCT ($p < .05$). **c**) Titration curves of fully mutated cadmium and strontium transporters in strain BCT were performed for Cd or Sr, respectively, with or without 100 μM Mn; x-axis represents the concentration of either Cd, Sr, or Cd, Sr with Mn. **d**) Sequential uptake experiments, up to 4 rounds, were performed to measure the amount of cycles required for complete elimination of 100 μM cadmium or strontium in a mixture of 100 μM manganese. Images on the right are colorimetric detection of cadmium and manganese (there are no available

colorimetric assays for strontium at this concentration) showing selective preference for cadmium (no coloration) against native metal manganese (darkened well). For all data, the mean ± s.d. of three replicates are shown.

Discussion:
Agreed yeast expressing neomutated forms of Smf1p preferentially accumulate Sr or Cd over Mn; yet in absence of sufficient biochemical data or structural explanation care should be taken not to oversell the mutagenesis approach by qualifying the transporter metal selectivity.

For instance, l. 298-308, mCd preference for Cd is relative (Fig. 5d lower left) since it appears more clearly in the 2 first rounds of uptake, implying rather low affinity for Cd; so that it is a decrease in Mn affinity and increased permissiveness for Cd uptake that best describe mCd mutant.

There is no discussion of the possible impact of the mutations obtained on Smf1* structure ; 3D homology modelling should point the location of the mutated residues to show whether they colocalize and/or contribute to delineate some inner cavity used for metal translocation.

Mentioning the use of other compartments for metal accumulation such as Golgi, RE and mitochondria does not seem realistic: metal export from these compartments is key to maintaining their functional integrity (e.g., plant chloroplasts).

More generally the discussion should emphasize that eventually engineered hyperaccumulator yeasts should not be able to survive outside a defined industrial environment.

All points are valid, thank you for such a thoughtful response. We agree Cd or Sr preference is relative to the amount of Mn uptake. That is, the decrease in Mn affinity with respects to increased Cd and Sr uptake can also appropriately explain these results. This has been better emphasized in the Results (page 15-16). The statement is copied below:

These modifications, and each subsequent change, reduces Mn uptake while increasing uptake of Cd or Sr for mCd and mSr, respectively (Figure 5b). It should be noted that these mutations could impede Mn uptake allowing increased permissiveness of Cd and Sr transport, rather than strictly increasing sensitivity for Cd or Sr; a subtle yet important distinction.

Also in the results section is a discussion of the impact of the mutations on SMF1 structure. Based on the crystal structure 5KTE by Bozzi, mutations exist either in the inner cavity or extracellular outer facing regions. However, the crystal structure is lacking segments in TM1, 4 and 6, so much of these regions cannot be adequately highlighted and only estimated. We have copied a new Supplemental Figure 11 showing the mutated regions below:

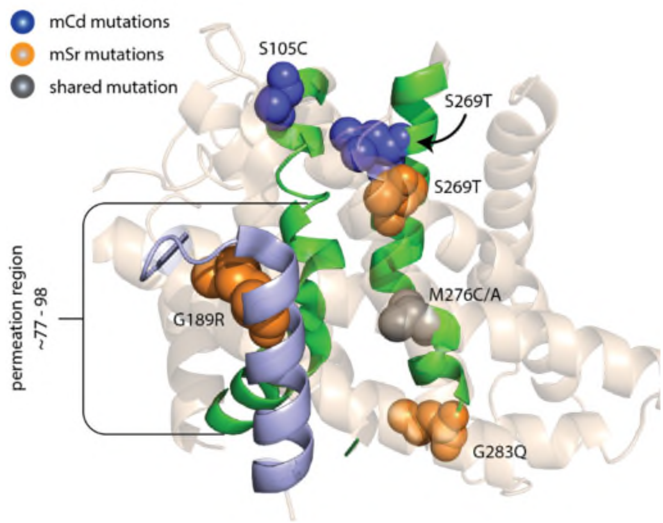

Supplemental Figure 11 | Approximate mutation locations for mCd and mSr are highlighted on homologue DraNramp (PDB 5KTE). Many of the mutations for both mCd and mSr reside on TM6, or at the entry of TM1. This could suggest that the region 77-98 (41-61 for DraNramp) in the first alpha-helix sequence of TM1 is highly sensitive to mutations, as observed in previous works[33,35,36]. This region, referred to as the permeation region[33], has a highly conserved DPGN sequence which may act as an actuator to transport metals through the inner cavity. Whereas, TM6 and 4 may provide the spacing and environment to select for certain metals.

Supplemental 12 (copied in a previous response) shows the expression levels of each mutant for mCd and mSr. A statement about these new results can be found in the Results, and copied below:

To test the contributions of each mutation, SMF1* was systematically mutated at each of the changed residues to reveal their significance and effect on SMF1 expression and function. Many of the mutations on mCd and mSr were located on TM6 rather than TM1, which supports past observations of the highly sensitive permeation region in the first alpha-helix segment of TM1 (Supplemental Figure 11). In addition, rounds of mutations leading to mCd and mSr did not significantly change expression levels (Supplemental Figure 12).

As for discussions around antiporters, we agree that these proteins are the next frontier to compartmentalize metals for enhanced metal uptake. We make a note of anti-porters in Figure 1a, figure legend, and accompanying text, and have dedicated a section in the Discussion (page 18) to highlight this, copied below:

A complementary approach, which is currently being investigated, is whether the deletion of metal antiporters can improve metal retention in organelles and enhance overall metal accumulation. It may be a promising strategy to delete antiporters from organelles, such as the Golgi, ER, mitochondria, etc. to gradually build up metal compartmentalization. This is

particularly interesting, and also straightforward, if there is no good metal transporter candidate from these organelles, or if they are difficult to rationally engineer.

Finally, in the discussion we have mentioned our vision for how these yeast can be used industrially, and have added a discussion of why, and how these yeast, will be contained in their industrial settings. We note the possibility of incorporation kill switches, or nutrient reliance to safeguard escape, and added the appropriate reference in the text (page 17-18).

Actual application of these yeast strains in real-world settings would require another layer of technological development, such as a container or cartridge to secure yeast in a controllable unit. Fortunately, these technologies exist, such as yeast packaging, freeze-drying, and delivery which are routine technologies found in the consumer market. A potential concept is to grow and store yeast in commercial filter-like cartridges where they can be housed in filtering units with size-exclusion cutoffs to prevent yeast leakage back into the purified waters. An additional layer of safety is to genetically modify these yeast with kill switches, or a metabolic reliance on a controlled nutrient such that removal from these containers will result in cell death[45].

Reviewers' comments:

Reviewer #1 (Remarks to the Author):
Unavailable.

Reviewer #2 (Remarks to the Author):

All the questions we raised have been addressed and most to a satisfactory degree. However, an issue remains that has to be addressed experimentally before this work can be published:

1) The authors should determine experimentally the dry weight of all strains compared to plant hyperaccumulators. The authors use a definition of a hyperaccumulator as an organism accumulates more than a certain amount of a specific metal/kg dry weight. Yeast growth of each strain is measured as optical density (OD) and then converted to dry weight by using a correlation factor. The calculated dry weight is then further used for calculating the whether hyperaccumulation takes place. It is obvious that the correlation factor must be determined carefully before this calculation can become convincing. However, correlation factors have still only been determined for the WT and a single strain (S*BCT) and are claimed to be identical for which reason a single correlation factor is used for all strains. However, not only do the strains differ in this work, but also the media in which the strains have been grown. How do the different strains and media affect dry weight? The authors provide references that they argue support their view that a single correlation factor can be used for multiple yeast strains and that media are not important. This is, however, not what the references provided by the authors state (see e.g.
https://www.researchgate.net/post/How_do_you_correlate_OD_measurements_with_cell_dry_weight and https://aem.asm.org/content/69/4/1990.short).

It is argued that it would be too difficult to measure the dry weight directly following growth of all media and for all those strains that have been compared with plant hyperaccumulators. This logic is hard to understand. The authors argues that it is difficult to measure the dry weight due to the yeast becoming flaky/static and sticking to the tube walls, making it difficult to transfer the material to a scale, and having a milligram to submilligram mass. Why should it be necessary at all to move the dry yeast cells from the tube in which they were dried and why should it be a challenge to measure milligram to submiligram masses within these tubes themselves? In fact, in the Materials and Method section, the authors clearly describe a procedure for weighing dried yeast inside the tube in which they were dried: Line 554- 560.

Minor issue:
1) Figure 3.d: It is stated that the titration experiments are performed from 1-100 µM, however it appears from the figure that measurements above 100 µM have been made.

<i>Editor</i>-following is this reviewer's comment on your responses to Reviewer #1, which was stated in the Remark to Editor section of his/her review report:

The authors have satisfactorily addressed most concerns. However, there is one remaining issue that should be clarified before this work can be recommended for publication. The authors were asked to differentiate between uptake/internalization in the accumulating yeast strains as opposed to binding to cell walls and the plasma membrane. This could have been demonstrated by performing e.g. cell wash experiments as was suggested or labeling experiments/staining for metals taken up into internal stores, which could be visualized by bioimaging methods. The author’s reply that the WT was included as a control does not address this issue. Likewise the examination of unspecific binding to the test tubes does not address the issue raised.

Reviewer #3 (Remarks to the Author):

The revised version of the manuscript addresses some of my original concerns. However, two of my concerns remain. Firstly, the novelty of the manuscript is (in part) limited to the simultaneous co-expression of different genes already known individually (or in some cases combined 2 by 2) to contribute to metal uptake. Secondly, the claim that the mutant versions of SMF1 have altered substrate specificities is not supported by solid biochemical data (e.g. quantification of protein amounts in the plasma membrane and/or reconstitution experiments). On the other hand, I think the authors did a lot of work, the experiments were done carefully, and the (revised) manuscript was clearly improved. Importantly, the density-based screening approach to identify metal hyper-accumulators coupled to colorimetric assays for metal specificity is a clever and rewarding strategy to engineer yeast for metal bioremediation. Despite the shortcomings above, I am leaning towards recommending acceptance of this manuscript.

Reviewer #4 (Remarks to the Author):

Several points have not been settled:
p.19
"units of uM of metal removed per cell culture OD (uM/OD)"
Getting a similar correlation between OD and dry weight with WT and the fully engineered S*BCT strain is satisfactory.
However, it is disturbing that "metal removal" measurement is used to claim "metal hyperaccumulation". Accumulation implies metals are internalized and it has not been demonstrated it is actually the case.
A quick filtration assay (using filtering units with size exclusion cutoffs) would allow collecting yeast samples easily, washing extracellular metal ions adsorbed to yeast cell wall, and thus measuring internalized metal with confidence.

p.21, second comment.
"a WT sample (non-expressing strain) was processed under similar conditions and measured for metal uptake"
The above control is not a suitable because it does not rule out the possibility that yeast overexpressing transporters may adsorb significant amount of metals at their surface. Rigorous testing of uptake should measure as well the difference between binding (measured at 4C) and active uptake.

p.22
The criticism has not been answered: how was the actual site of protein expression verified for the membrane transporters and the vacuole transporters presented in Figure S1? Due to over expression it is possible that the actual transporter location was affected.
Higher magnification and/or use of native reporters of membrane compartments, or membrane preparations tested by western blot, could convince that say a "vacuole transporter" was indeed overexpressed in the vacuole membrane.

l. 100: metal exporters...
SMF3 is NOT a vacuole importer; it is an exporter that transports Fe and Mn out of the vacuole into the cytoplasm. Are there any functional evidence demonstrating that SMF3 functions as antiporter?

p.23, II. Titration of uptake:
Data presentation makes it difficult to appreciate an "hyperaccumulator" activity.

p.24, III. Isolation of metal hyperaccumulating mutants

OK

p.25, 4.
OK

p.26, Only two mutants,...
OK

p.27, statement clarifications
l. 67-68
In the previous version of the Ms, nickel was listed twice (1% dry weight and 0.1% dry weight). The authors' answer does not settle the case: 1,000 mg/kg is NOT equivalent to 1% dry weight. The values cited should be clarified regarding what was actually measured, whole plant or specific part, so as to allow better comparison with the present yeast study.

l. 108
OK

l. 300-302 vs l. 766-767
OK

-Fig.1b
a presentation like that used in Suppl. Fig. 5 would preserve data of the WT reference

p.28,
-Fig. 3c and 3d
OK

-Fig. 5b and 5c: M276 or M267?
OK

Discussion:
OK, except
"the crystal structure 5KTE by Bozzi... is lacking segments in TM1, 4 and 6,... so much of these regions cannot be adequately highlighted"
Aren't there additional structures of SMF homologs that could be used instead; authors mentioned "The crystal structures of SMF1 homologues D. radiodurans (DraNramp) and S. capitis (ScaDMT)" for instance.

"As for discussions around antiporters, ..."
Exporters are not necessarily antiporters, eg, SMF3 exports metals from the vacuole and is expected to function similarly to SMF1, ie both H+ and Me2+ being carried in the same direction.

Reviewers' comments:

Reviewer #1 (Remarks to the Author):
Unavailable.

Reviewer #2 (Remarks to the Author):

All the questions we raised have been addressed and most to a satisfactory degree. However, an issue remains that has to be addressed experimentally before this work can be published:

1) The authors should determine experimentally the dry weight of all strains compared to plant hyperaccumulators. The authors use a definition of a hyperaccumulator as an organism accumulates more than a certain amount of a specific metal/kg dry weight. Yeast growth of each strain is measured as optical density (OD) and then converted to dry weight by using a correlation factor. The calculated dry weight is then further used for calculating the whether hyperaccumulation takes place. It is obvious that the correlation factor must be determined carefully before this calculation can become convincing. However, correlation factors have still only been determined for the WT and a single strain (S*BCT) and are claimed to be identical for which reason a single correlation factor is used for all strains. However, not only do the strains differ in this work, but also the media in which the strains have been grown. How do the different strains and media affect dry weight? The authors provide references that they argue support their view that a single correlation factor can be used for multiple yeast strains and that media are not important. This is, however, not what the references provided by the authors state (see e.g.
https://www.researchgate.net/post/How_do_you_correlate_OD_measurements_with_cell_dry_weight and https://aem.asm.org/content/69/4/1990.short).

It is argued that it would be too difficult to measure the dry weight directly following growth of all media and for all those strains that have been compared with plant hyperaccumulators. This logic is hard to understand. The authors argues that it is difficult to measure the dry weight due to the yeast becoming flaky/static and sticking to the tube walls, making it difficult to transfer the material to a scale, and having a milligram to submilligram mass. Why should it be necessary at all to move the dry yeast cells from the tube in which they were dried and why should it be a challenge to measure milligram to submiligram masses within these tubes themselves? In fact, in the Materials and Method section, the authors clearly describe a procedure for weighing dried yeast inside the tube in which they were dried: Line 554- 560.

Thank you for motivating us to perform a more meticulous OD to dry weight calculation. We have experimentally determined the correlation factor for all strains which we report in the manuscript. Supplemental Figure 4 (previously 3) has been heavily edited to contain the correlation plot for all strains (which was done for WT and SBC*T in the last-revised manuscript). On average, the OD to dry weight ratio was slightly greater than 0.5 g/L per OD_{600}.

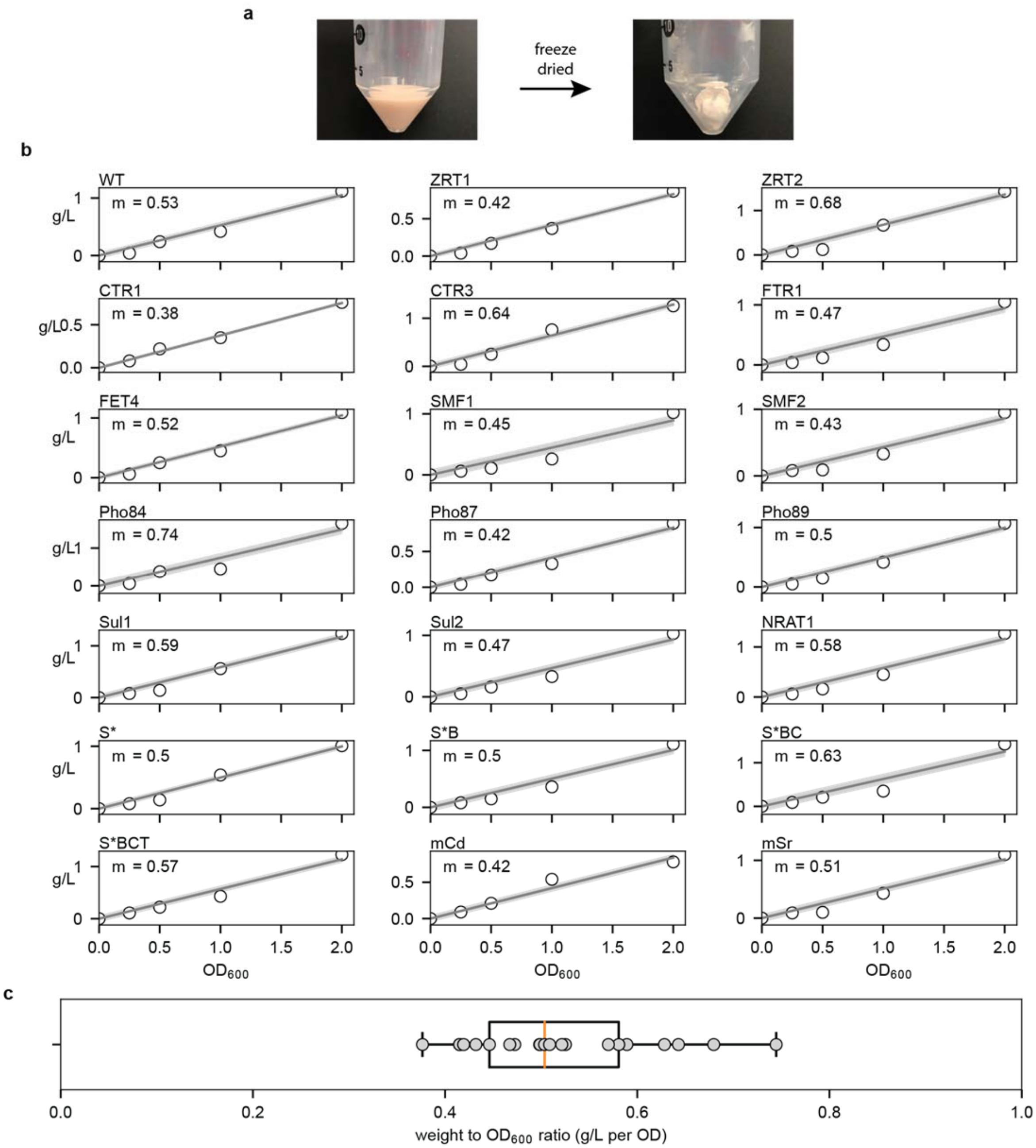

Supplemental Figure 4 | Correlating culture optical density (OD_{600}) to grams of culture dry weight (gDW). a) Cells were grown, washed, pelleted, and freeze-dried to obtain culture dry weight per culture volume. Masses were weighed on a precision scale with hundredths of milligram resolution. **b**) A line of best fit with intercept at 0 was performed to obtain a correlation factor between OD_{600} and gram of dry weight per culture volume for each strain. **c**) A

box plot of all OD_{600} to gram dry weight correlation factors. On average the correlation factor was approximately 0.52 g/L per OD_{600}.

What changed in the manuscript are re-calculations of the hyperaccumulation values stated on Pages 8-10, and a re-calculated Supplemental Table 1 which accounts for the different OD to dry weight correlation factors per strain. These new values were used to convert uptake values of μM to mg/g to compare against hyperaccumulation thresholds.

strain	metal	uptake (μM)	uptake (mg/g)	hyper threshold (mg/g)
ZRT1	Zn	46 ± 6.7	7.3 ± 1.1	10
ZRT2	Zn	35 ± 7.5	3.4 ± 0.7	10
CTR1	Cu	44 ± 5.6	7.5 ± 0.9	1.0
CTR3	Cu	31 ± 7.0	3.1 ± 0.7	1.0
FTR1	Fe	17 ± 3.7	2.0 ± 0.4	10
FET4	Fe	23 ± 2.9	2.5 ± 0.3	10
SMF1	Mn	11 ± 2.1	1.3 ± 0.3	10
SMF2	Mn	10 ± 2.6	1.2 ± 0.3	10
Pho84	As	28 ± 3.8	2.8 ± 0.4	0.1
Pho87	As	13 ± 1.0	2.4 ± 0.2	0.1
Pho89	As	18 ± 3.0	2.8 ± 0.5	0.1
Sul1	Cr	25 ± 2.4	2.2 ± 0.2	1.0
Sul2	Cr	24 ± 1.9	2.7 ± 0.2	1.0
NRAT1	Al	27 ± 4.3	1.2 ± 0.2	1.0
S*	Cd	10 ± 1.3	2.2 ± 0.3	0.1
S*B	Cd	21 ± 2.5	4.8 ± 0.6	0.1
S*BC	Cd	53 ± 7.6	9.5 ± 1.4	0.1
S*BCT	Cd	58 ± 5.4	11.4 ± 1.1	0.1
S*BCT	Mn	328 ± 14.8	31.7 ± 1.4	10
mCd	Cd	43 ± 4.6	11.5 ± 1.2	0.1
mSr	Sr	30 ± 3.9	5.1 ± 0.7	N/A

Supplemental Table 1 | Transporters and strains developed in this work that are within or have exceeded the hyperaccumulating thresholds[12,13] for their respective metal.

We would like to provide a sufficient answer as to why we calculated the OD to dry weight correlation factor in a separate experiment than during the metal uptake experiments. In our metal uptake experiments we used 5 mL of yeast culture in 14 mL inoculation tubes. When we attempted to lyophilize these samples the yeast mass were so small that it was barely detectable, only reaching one significant digit on our scales. In addition, the mass size would be affected by static and minor breezes that sometimes flaked off or would escape in the lyophilization chamber. Therefore, what we describe in the Material and Methods section was to grow a larger 500 mL culture volume and transfer the pelleted mass into a 50 mL conical tube. With the larger volume, the mass of the culture could be more accurately determined. Therefore, we performed separate experiments at larger volumes to determine the OD to dry weight correlation factor for each strain.

Minor issue:
1) Figure 3.d: It is stated that the titration experiments are performed from 1-100 μM, however it appears from the figure that measurements above 100 μM have been made.

Thank you for pointing this out. You are right, we incorrectly used the wrong upper bound and corrected it to 1 mM. The titration experiments were 10-fold higher than our typical metal uptake experiments. The final concentration was 1 mM which has been changed in Figure 3d and edited in the Material and Methods section as well.

<i>Editor</i>-following is this reviewer's comment on your responses to Reviewer #1, which was stated in the Remark to Editor section of his/her review report:

The authors have satisfactorily addressed most concerns. However, there is one remaining issue that should be clarified before this work can be recommended for publication. The authors were asked to differentiate between uptake/internalization in the accumulating yeast strains as opposed to binding to cell walls and the plasma membrane. This could have been demonstrated by performing e.g. cell wash experiments as was suggested or labeling experiments/staining for metals taken up into internal stores, which could be visualized by bioimaging methods. The author's reply that the WT was included as a control does not address this issue. Likewise the examination of unspecific binding to the test tubes does not address the issue raised.

We have performed a more rigorous control to consider the possibility of non-specific metal binding onto the cell wall. As the reviewer suggested, we performed several wash steps with both ddH$_2$O and EDTA buffer to assess the amount of metal freed per wash step; signifying the amount of metal absorbed on to the cell wall. The protocol followed:

Cells were spun down and supernatant removed for ICP analysis (following our metal uptake experiment protocol on Page 27), cells were washed once with ddH$_2$O to remove any residual liquid, as not all the liquid was removed for ICP analysis. Afterwards, the cells were washed once more with ddH$_2$O to the original volume (e.g. 5 mL), cells spun down, and supernatant removed and measured for metal content. Finally, cells were washed a final time in EDTA buffer (10 mM Tris with 1 mM EDTA, pH 7.4) to the original volume of 5 mL, pelleted, supernatant removed and measured for metal content. Overall, the cells were washed several times, with each wash step measured by ICP for freed metal content.

This additional protocol has been added to our Materials and Methods section on Page 27.

Data from this experiment could not be concisely summarized in a subfigure in the main text, therefore it was made as Supplemental Figure 2, and all numberings have been readjusted.

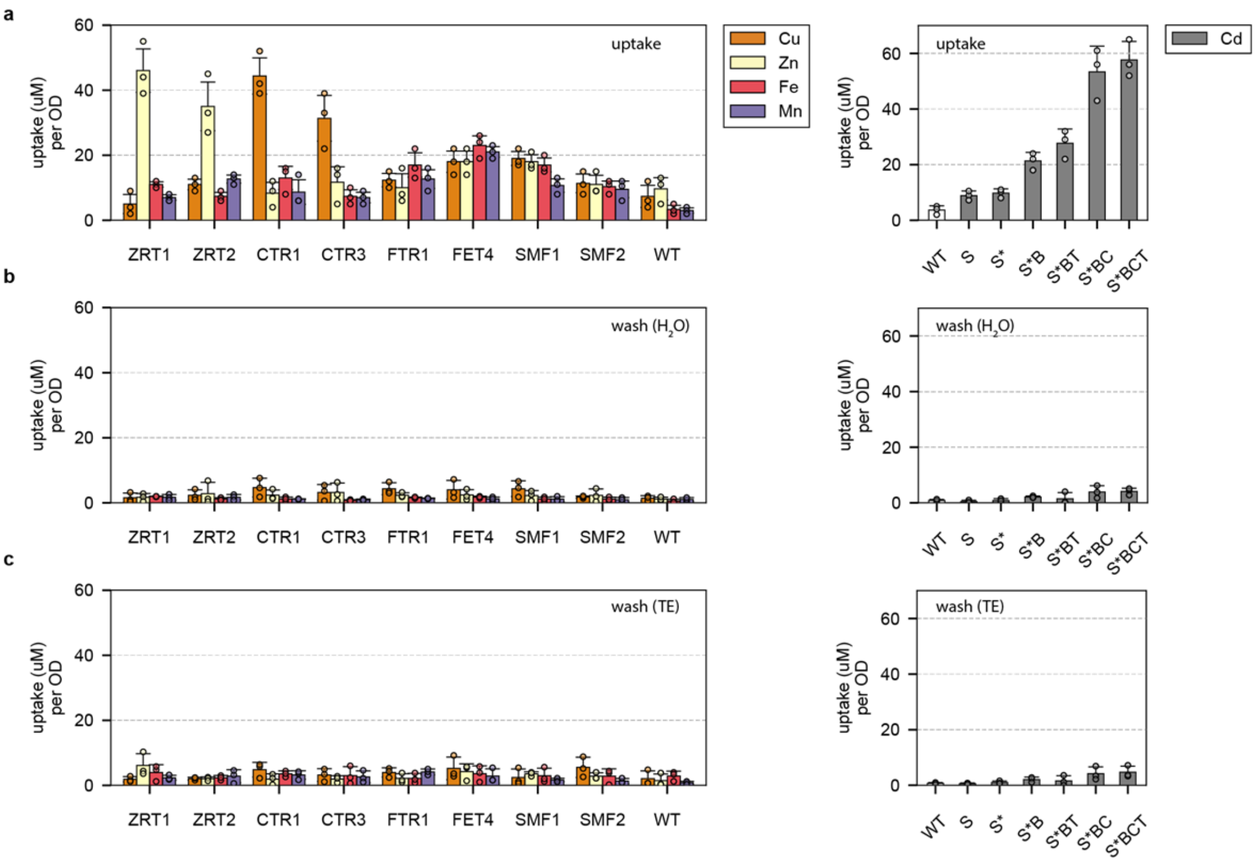

Supplemental Figure 1 | Impact of non-specific metal binding during metal uptake experiments. a) Measurement of metal removed from a 100 μM metal uptake experiment (refer to Figure 1b and 3a). **b**) After a metal uptake experiment, cells were washed with ddH_2O and supernatant measured for freed metal. **c**) After the ddH_2O wash step, another wash step in a 1 mM EDTA buffer was similarly measured for freed metal.

The data suggest that non-specific metal binding was not a major contributor to our metal hyperaccumulation results. More so, one concern about the wash method is that during the wash steps, it may be possible that cells could lyse in the un-buffered ddH_2O wash step, and possibly in the EDTA buffer, thereby releasing internalized metals and overestimating results.

In addition, we performed a theoretical calculation on the possible amount of non-specific binding that can occur on the cell wall. Considering our experimental parameters: 5 mL of 1 OD_{600} cell, or approximately 10 million cells per mL per metal uptake experiment; an estimated expression level of 1 million transporters per cell (a generous estimate considering yeast display generates only thousands of displayed copies per cell); and each transporter having non-specific binding to 1 metal; multiplying these values together gives approximately 2 picomolar of non-specific metal capture. Compared to 10-100 μM of metal uptake we see in our experiments, almost 3 orders of magnitude more. This demonstrates

that the surface-to-volume ratio of yeast allows more metal to be internalized in the intracellular volume, rather than the cell surface. We hope these additional experiments also validate this hypothesis.

Reviewer #3 (Remarks to the Author):

The revised version of the manuscript addresses some of my original concerns. However, two of my concerns remain. Firstly, the novelty of the manuscript is (in part) limited to the simultaneous co-expression of different genes already known individually (or in some cases combined 2 by 2) to contribute to metal uptake. Secondly, the claim that the mutant versions of SMF1 have altered substrate specificities is not supported by solid biochemical data (e.g. quantification of protein amounts in the plasma membrane and/or reconstitution experiments). On the other hand, I think the authors did a lot of work, the experiments were done carefully, and the (revised) manuscript was clearly improved. Importantly, the density-based screening approach to identify metal hyper-accumulators coupled to colorimetric assays for metal specificity is a clever and rewarding strategy to engineer yeast for metal bioremediation. Despite the shortcomings above, I am leaning towards recommending acceptance of this manuscript.

We realize that our manuscript did not provide a comprehensive analysis on the biochemical nature of metal transporters and their specificity, but the hope of this manuscript was to inform bioengineers that metal removal for water remediation could be effectively engineered in yeast using metal transporters.

Therefore, in our manuscript we aimed to highlight the engineering constraints and design criteria needed to make yeast hyperaccumulators; this included engineering high expression of a metal transporter, co-expression of a vacuole transporter, introduction of a metal tolerant mechanism, and finally designing a metal transporter screening assay using a coupled density-gradient and colorimetric assay to select for desired mutants. These same considerations could be used for other hosts of interest; however, if the interest is still yeast there are more discoveries to be made, such as precious metal transport of dissolved noble metals (i.e. gold, silver), or conversion of metals once internalized. These research questions, discussed in the Discussion section, are subsequent research goals which we are continuing to investigate as a product of this work.

Reviewer #4 (Remarks to the Author):

Several points have not been settled:
p.19
"units of uM of metal removed per cell culture OD (uM/OD)"
Getting a similar correlation between OD and dry weight with WT and the fully engineered S*BCT strain is satisfactory.
However, it is disturbing that "metal removal" measurement is used to claim "metal hyperaccumulation". Accumulation implies metals are internalized and it has not been demonstrated it is actually the case.
A quick filtration assay (using filtering units with size exclusion cutoffs) would allow collecting yeast samples easily, washing extracellular metal ions adsorbed to yeast cell wall, and thus measuring internalized metal with confidence.

p.21, second comment.
"a WT sample (non-expressing strain) was processed under similar conditions and measured for metal uptake"
The above control is not a suitable because it does not rule out the possibility that yeast overexpressing transporters may adsorb significant amount of metals at their surface. Rigorous testing of uptake should measure as well the difference between binding (measured at 4C) and active uptake.

Given the reviews, we were motivated to perform a more meticulous OD to dry weight calculation. We have experimentally determined the correlation factor for all strains which we report in the manuscript. Supplemental Figure 4 (previously 3) has been heavily edited to contain the correlation plot for all strains (which was done for WT and SBC*T in the last-revised manuscript). On average, the OD to dry weight ratio was slightly greater than 0.5 g/L per OD_{600}.

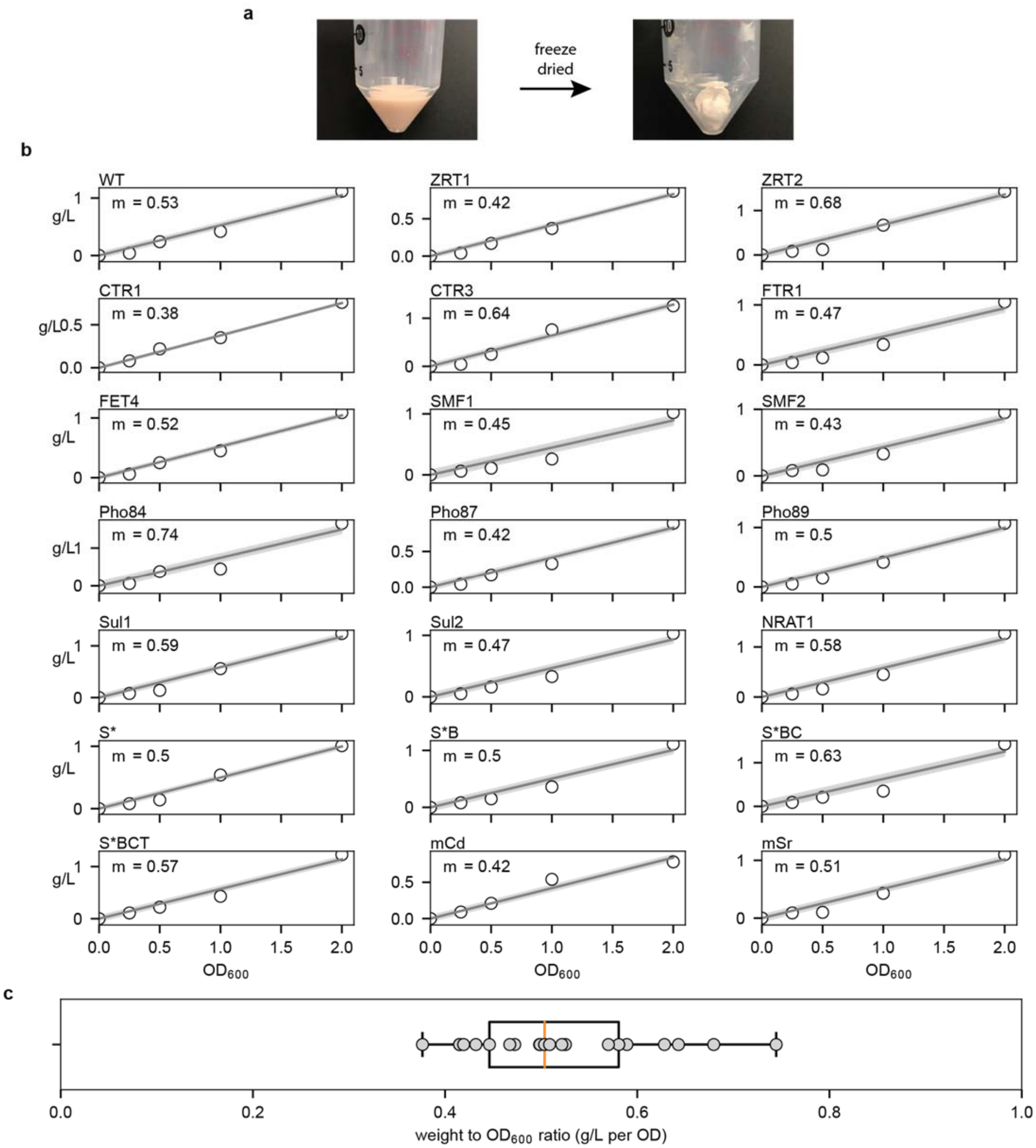

Supplemental Figure 4 | Correlating culture optical density (OD_{600}) to grams of culture dry weight (gDW). a) Cells were grown, washed, pelleted, and freeze-dried to obtain culture dry weight per culture volume. Masses were weighed on a precision scale with hundredths of milligram resolution. **b**) A line of best fit with intercept at 0 was performed to obtain a correlation factor between OD_{600} and gram of dry weight per culture volume for each strain. **c**) A

box plot of all OD_{600} to gram dry weight correlation factors. On average the correlation factor was approximately 0.52 g/L per OD_{600}.

What changed in the manuscript are re-calculations of the hyperaccumulation values stated on Pages 8-10, and a re-calculated Supplemental Table 1 which accounts for the different OD to dry weight correlation factors per strain. These new values were used to convert uptake values of μM to mg/g to compare against hyperaccumulation thresholds.

strain	metal	uptake (μM)	uptake (mg/g)	hyper threshold (mg/g)
ZRT1	Zn	46 ± 6.7	7.3 ± 1.1	10
ZRT2	Zn	35 ± 7.5	3.4 ± 0.7	10
CTR1	Cu	44 ± 5.6	7.5 ± 0.9	1.0
CTR3	Cu	31 ± 7.0	3.1 ± 0.7	1.0
FTR1	Fe	17 ± 3.7	2.0 ± 0.4	10
FET4	Fe	23 ± 2.9	2.5 ± 0.3	10
SMF1	Mn	11 ± 2.1	1.3 ± 0.3	10
SMF2	Mn	10 ± 2.6	1.2 ± 0.3	10
Pho84	As	28 ± 3.8	2.8 ± 0.4	0.1
Pho87	As	13 ± 1.0	2.4 ± 0.2	0.1
Pho89	As	18 ± 3.0	2.8 ± 0.5	0.1
Sul1	Cr	25 ± 2.4	2.2 ± 0.2	1.0
Sul2	Cr	24 ± 1.9	2.7 ± 0.2	1.0
NRAT1	Al	27 ± 4.3	1.2 ± 0.2	1.0
S*	Cd	10 ± 1.3	2.2 ± 0.3	0.1
S*B	Cd	21 ± 2.5	4.8 ± 0.6	0.1
S*BC	Cd	53 ± 7.6	9.5 ± 1.4	0.1
S*BCT	Cd	58 ± 5.4	11.4 ± 1.1	0.1
S*BCT	Mn	328 ± 14.8	31.7 ± 1.4	10
mCd	Cd	43 ± 4.6	11.5 ± 1.2	0.1
mSr	Sr	30 ± 3.9	5.1 ± 0.7	N/A

Supplemental Table 1 | Transporters and strains developed in this work that are within or have exceeded the hyperaccumulating thresholds[12,13] for their respective metal.

To address your second concern, we have performed a more rigorous control to consider the possibility of non-specific metal binding on to the cell wall. As the reviewer suggested, we performed several wash steps with both ddH_2O and EDTA buffer to assess the amount of metal freed per wash step; signifying the amount of metal absorbed onto the cell wall. The protocol followed:

Cells were spun down and supernatant removed for ICP analysis (following our metal uptake experiment protocol on Page 27), cells were washed once with ddH_2O to remove any residual liquid, as not all the liquid was removed for ICP analysis. Afterwards, the cells were washed once more with ddH_2O to the original volume (e.g. 5 mL), cells spun down, and supernatant removed and measured for metal content. Finally, cells were washed a final time in EDTA buffer (10 mM Tris with 1 mM EDTA, pH 7.4) to the original volume of 5 mL, pelleted, supernatant removed and measured for metal content. Overall, the cells were washed several times, with each wash step measured by ICP for freed metal content.

This additional protocol has been added to our Materials and Methods section on Page 27.

Data from this experiment could not be concisely summarized in a subfigure in the main text, therefore it was made as Supplemental Figure 2, and all numberings have been readjusted.

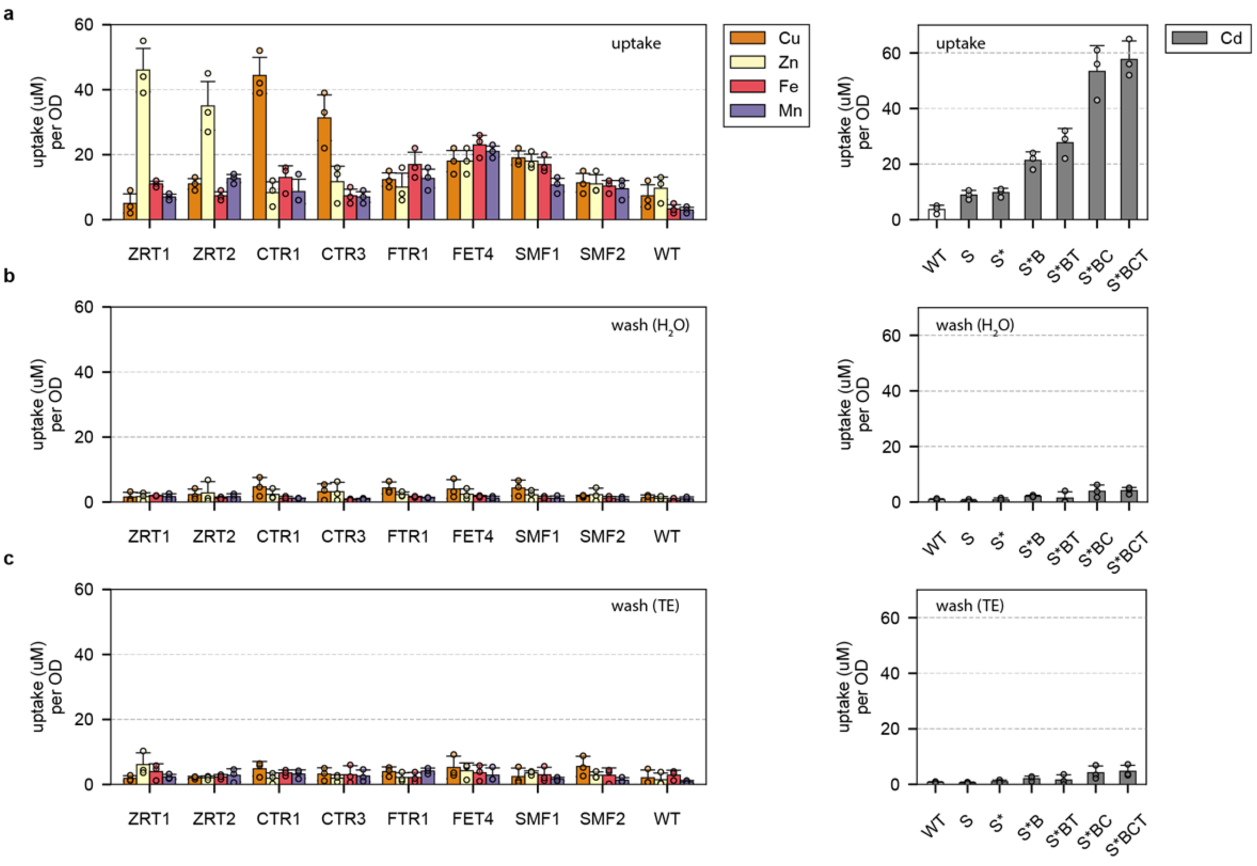

Supplemental Figure 2 | Impact of non-specific metal binding during metal uptake experiments. a) Measurement of metal removed from a 100 μM metal uptake experiment (refer to Figure 1b and 3a). **b**) After a metal uptake experiment, cells were washed with ddH_2O and supernatant measured for freed metal. **c**) After the ddH_2O wash step, another wash step in a 1 mM EDTA buffer was similarly measured for freed metal.

The data suggest that non-specific metal binding was not a major contributor to our metal hyperaccumulation results. More so, one concern about the wash method is that during the wash steps, it may be possible that cells could lyse in the un-buffered ddH_2O wash step, and possibly in the EDTA buffer, thereby releasing internalized metals and overestimating results.

In addition, we performed a theoretical calculation on the possible amount of non-specific binding that can occur on the cell wall. Considering our experimental parameters: 5 mL of 1 OD_{600} cell, or approximately 10 million cells per mL per metal uptake experiment; an

estimated expression level of 1 million transporters per cell (a generous estimate considering yeast display generates only thousands of displayed copies per cell); and each transporter having non-specific binding to 1 metal; multiplying these values together gives approximately 2 picomolar of non-specific metal capture. Compared to 10-100 μM of metal uptake we see in our experiments, almost 3 orders of magnitude more. This demonstrates that the surface-to-volume ratio of yeast allows more metal to be internalized in the intracellular volume, rather than the cell surface. We hope these additional experiments also validate this hypothesis.

p.22
The criticism has not been answered: how was the actual site of protein expression verified for the membrane transporters and the vacuole transporters presented in Figure S1? Due to over expression it is possible that the actual transporter location was affected.
Higher magnification and/or use of native reporters of membrane compartments, or membrane preparations tested by western blot, could convince that say a "vacuole transporter" was indeed overexpressed in the vacuole membrane.

Protein expression was verified by fluorescence imaging, this approach was qualitatively measured to discern which transporters gave the more uniform expression and did not form punctate or cytoplasmic patterns. This helped us narrow our transporter selection to SMF1, as it showed the best expression intensity and uniformity. The same was true for the vacuole transporters when selecting CCC1 as our candidate vacuole transporter.

When we were setting up our initial fluorescence imaging experiments, we used membrane-specific dyes as references to determine the location of the cell membrane and vacuole membrane regions. These initial experiments helped us compare our fluorescently stained images to what an expected membrane and vacuole regions looked like.

The fluorescent microscope we had access to had 100X magnification. We used a DIC filter to achieve better contrast of the vacuole outline. Larger magnifications and higher resolution of the fluorescent images for the vacuole transporters from Supp. Fig. 1 is provided below to better visualize the vacuole expression and morphology.

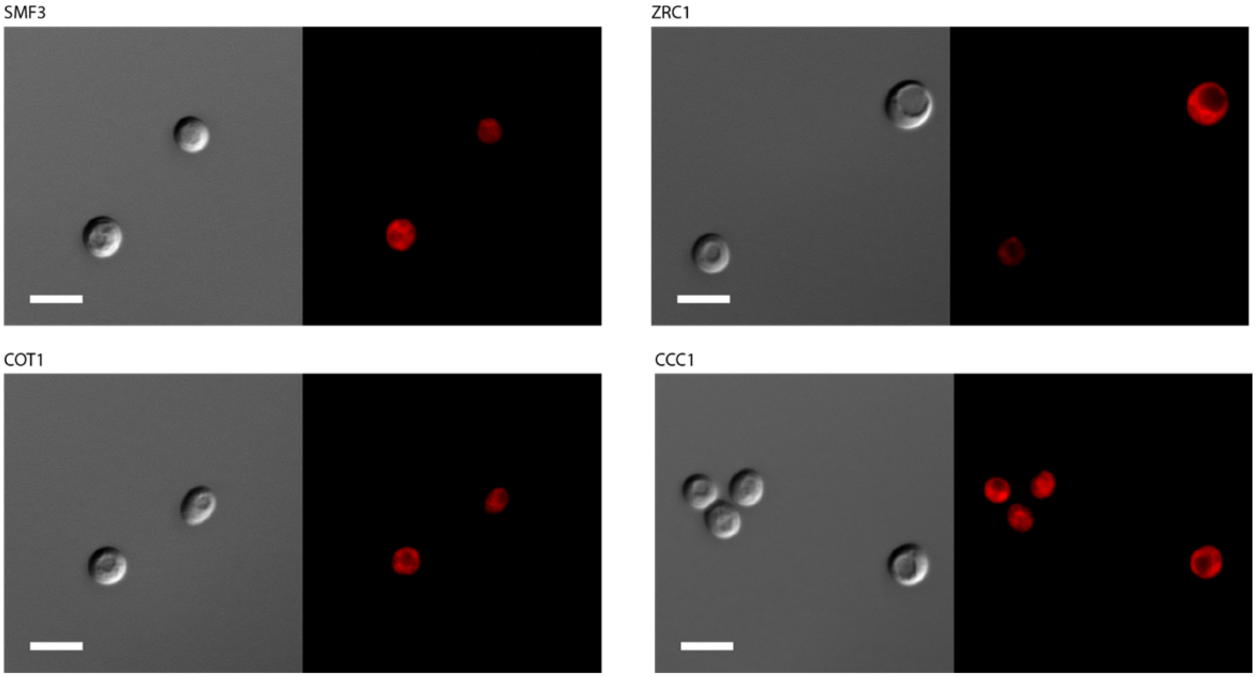

l. 100: metal exporters...
SMF3 is NOT a vacuole importer; it is an exporter that transports Fe and Mn out of the vacuole into the cytoplasm. Are there any functional evidence demonstrating that SMF3 functions as antiporter?

We may have mislabeled SMF3 as a vacuole importer in our results and discussion section, and apologize for the confusion. SMF3 is an exporter, and this language has been changed throughout the text. Thank you for clearing this up for us.

We have read several past research studies into the SMF1, SMF2, and SMF3 family of metal transporters and thought co-expression of SMF1 and SMF3 may lead to greater metal uptake or survivability. As seen in Fig. 2b, the change in metal uptake with SMF1, and SMF1 with SMF3 was not significant compared to SMF1 expression alone, suggesting that SMF3 may not help in metal sequestration into the vacuole, and not demonstrating antiporter behavior.

p.23, II. Titration of uptake:
Data presentation makes it difficult to appreciate an "hyperaccumulator" activity.

p.24, III. Isolation of metal hyperaccumulating mutants
OK

p.25, 4.
OK

p.26, Only two mutants,...
OK

p.27, statement clarifications
l. 67-68
In the previous version of the Ms, nickel was listed twice (1% dry weight and 0.1% dry weight).
The authors' answer does not settle the case: 1,000 mg/kg is NOT equivalent to 1% dry weight.
The values cited should be clarified regarding what was actually measured, whole plant or specific part, so as to allow better comparison with the present yeast study.

Apologies for the confusion, hyperaccumulation threshold of nickel is defined to be 1,000 mg/kg, which is 0.1% dry weight. This distinction has been highlighted in the manuscript on Page 3, and we hope this clarifies this issue.

l. 108
OK

l. 300-302 vs l. 766-767
OK

-Fig.1b
a presentation like that used in Suppl. Fig. 5 would preserve data of the WT reference

p.28,
-Fig. 3c and 3d
OK

-Fig. 5b and 5c: M276 or M267?
OK

Discussion:
OK, except
"the crystal structure 5KTE by Bozzi... is lacking segments in TM1, 4 and 6,... so much of these regions cannot be adequately highlighted"
Aren't there additional structures of SMF homologs that could be used instead; authors mentioned "The crystal structures of SMF1 homologues D. radiodurans (DraNramp) and S. capitis (ScaDMT)" for instance.

Unfortunately, the crystal structure of ScaDMT (4WGW) from Ehrnstorfer et al.'s work is not complete and missing TM1a, an essential part of the metal trafficking mechanism in TM1 and TM6. Other than the crystal structure of DraNramp (5KTE) from Bozzi's work, there is no other similar transporter to SMF1 that could be referenced.

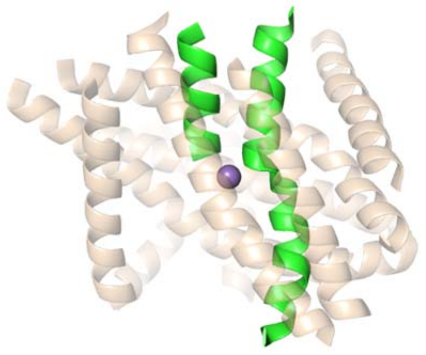

Image of ScaDMT (4WGW) with highlighted TM1 and TM6 regions, the absent helix on the bottom left shows the missing TM1a section.

"As for discussions around antiporters, ..."
Exporters are not necessarily antiporters, eg, SMF3 exports metals from the vacuole and is expected to function similarly to SMF1, ie both H+ and Me2+ being carried in the same direction.

Apologies for the confusing usage of exporters and antiporters. As a general category, metal exporters (not just antiporters) are an interesting class of metal transporters, SMF3

being one of them, which we discussed in the Discussion section as proteins to knockout for enhanced metal retention (Page 18). What we wanted to convey was that transporters that pump metals back into the cytoplasm or out of the cell could be knocked-out to improve metal retention, and thereby hyperaccumulation. This effect may be more pronounced in conjunction with an active metal transporter that pumps metal into the cell, such as SMF1.

Thank you for the clarification. The manuscript has been edited to refer to exporters rather than just anti-porters.

REVIEWERS' COMMENTS:

Reviewer #1 unavailable.

Reviewer #2 (Remarks to the Author):

All previous concerns have now been addressed, and the manuscript can be recommend for publication.

Reviewer #3 (Remarks to the Author):

I have no further requests and recommend acceptance.

Markus Tamas

Reviewer #4 (Remarks to the Author):

All the most significant concerns have been fairly addressed by the authors.
The amended manuscript has been appreciably improved.
I therefore wish to recommend acceptance of the current manuscript for publication in Nature Communications.
